# Creative tendency of Chinese students: a meta-analysis, 1999–2025

**DOI:** 10.3389/fpsyg.2026.1957163

**Published:** 2026-10-20

**Authors:** Yi Wang, Huiyong Fan

**Affiliations:** 1 College of Educational Science, Bohai University, Jinzhou, China; 2 Center of Psychological Research and Education, Bohai University, Jinzhou, China

**Keywords:** Chinese students, creative tendency, creativity disposition, grade effect, meta-analysis, Williams' Creativity Assessment Packet

## Abstract

**Introduction:**

Over the past three decades, student creativity in mainland China has drawn considerable scholarly attention, accompanied by a surge in empirical studies on creative tendencies. Yet findings remain strikingly inconsistent: some report high levels of creativity, while others suggest marked declines. This discrepancy raises two central questions: what is the overall level of creative tendency, and how do scores differ across grade levels among mainland Chinese students? And what accounts for the variability across existing studies?

**Methods:**

To address these questions, we conducted a meta-analysis of 211 primary studies published between 1999 and 2025, all of which used Williams' Creativity Assessment Packet and collectively included 211 independent samples (*N* = 130,642).

**Results:**

Results indicated an overall mean creative tendency score of 2.158 on a 3-point Likert scale (95% CI [2.124, 2.192]). Grade level was a significant moderator: mean scores decreased successively from the elementary-school group to the high-school group, while the mean score in the university group was close to that in the middle-school group. A significant grade-level difference was also observed for the Imaginative dimension. In contrast, gender, hometown location, publication type, and study quality showed no significant moderating effects.

**Discussion:**

We also discussed potential drivers of these grade-related patterns, explore their theoretical and educational implications, and highlighted key directions for future research.

## 1 Introduction

### 1.1 Background

Creativity is regarded as a key competence for navigating complex, globalized, and increasingly digitalized economies and societies (Herlinawati et al., 2024; Kaufman and Sternberg, 2010; Vincent-Lancrin et al., 2019; Park et al., 2021). Creativity is the function of Person, Task, Situation, and their interactions (Sternberg, 2025; Sternberg and Lubart, 1995; Tromp and Sternberg, 2022). As a personal attribute, creative tendency - sometimes referred to as creativity disposition (see Akpur, 2024; Barron and Harrington, 1981, for comparison with creativity personality)—reflects the affective dimension of creativity (Feist, 1998; Lin and Wang, 1994; Williams, 1980) and is defined as an individual's willingness to engage in creative expression, often manifested through related personality traits (Wu, 2024).

Regarding the internal structure of creative tendency, scholars have proposed various frameworks (Dong, 1993; Gu and Zhou, 2008; Guilford, 1967; Tardif and Sternberg, 1988; Shen et al., 2005; Sternberg, 2025). Among these, the four-dimensional structural model comprising risk-taking, curiosity, imagination, and challenge has gained widespread recognition within the community of international researchers (Lin and Wang, 1994; Shen et al., 2005; Williams, 1980; Wu, 2024; Xia et al., 2017).

Creative tendency determine the performance of personal creativity. On the one hand, creative tendency can drive the application of domain relevant knowledge and skills, creative knowledge and methods, thereby stimulating creative behavior (Amabile, 1983, 1996; Feist, 1998, 2010; Lopez-Persem et al., 2024; Wu, 2024). On the other hand, creative tendency also interacts with tasks and situations (Sternberg, 2025; Sternberg and Lubart, 1995; Tromp and Sternberg, 2022). Specifically, an individual's creative tendency shapes their preference for selecting creative tasks, while task characteristics can either elicit or suppress the expression of that tendency. Situational contexts, operating through multiple levels of the ecological system, modulate the fit between the individual and the task. Together, these three elements—creative tendency, task, and situation—interact dynamically to influence the manifestation of individual creativity.

Over the past three decades, the creative tendency of students in mainland China has remained a focal topic for researchers. A literature search returned more than 800 academic publications examining creative tendency of Chinese students, which address questions including: what is the actual level of creative tendency among Chinese students? How does it change as students progress through grade levels? How can the level of creative tendency among Chinese students be enhanced, particularly while preparing for high-stakes examinations? However, those previous studies have either failed to provide scientifically sound and useful evidence or have reported contradictory findings.

### 1.2 Inconsistency in previous studies

Regarding the actual level of creative tendency among Chinese students, two contrasting viewpoints exist. The first viewpoint suggests that Chinese students score high on creative tendency. Shen and Shi (2010) conducted a survey of middle school students from China (*N* = 670), Japan (*N* = 208), the United Kingdom (*N* = 215), and Germany (*N* = 316). The results indicated that Chinese students scored higher than students from the other three countries on the curiosity dimension. Shen (2016) used the Williams Creativity Assessment Packet (WCAP) to compare students from Civil Aviation University of China (*N* = 68) with students from Florida Institute of Technology (*N* = 42). The findings revealed that Chinese students majoring in mechanical engineering exhibited higher total creative tendency scores than their American counterparts in the same major. Gu and Zhang (2014) also found in their comparison of creative tendency (including risk-taking, curiosity, and challenge dimensions) between Chinese students (*N* = 136) and American students (*N* = 100) that Chinese students scored higher overall than their American peers.

The second viewpoint proposes that Chinese students demonstrate relatively low creative tendency. Shen and Shi (2010) surveyed middle school students from China (*N* = 670), Japan (*N* = 208), the United Kingdom (*N* = 215), and Germany *(N* = 316). The findings indicated that Chinese students scored lower than Japanese and British students on the risk-taking dimension. Shen (2016) used the WCAP to compare students from the Civil Aviation University of China (*N* = 68) with students from the Florida Institute of Technology (*N* = 42). Across the overall samples comprising students from all majors, American students exhibited higher total creative tendency scores than Chinese students.

Despite the substantial number of studies, the overall level of creative tendency among mainland Chinese students remains unclear. Individual studies draw on different samples and educational stages, and their contrasting findings cannot establish whether the observed differences reflect a broader pattern or sample-specific variation. A meta-analysis of studies using a common measure can provide a pooled estimate and examine potential sources of variation across studies.

### 1.3 The potential moderators

#### 1.3.1 Locations of hometown

Hometown location may serve as an important moderating variable affecting creative tendency scores among Chinese students. Some studies support the view that significant differences exist between urban and rural students. Liu and Zhang (2017) employed the WCAP revised by Lin and Wang to survey over 2,000 students from multiple secondary schools in Tianjin, Chongqing, Sichuan, Yunnan, and other regions. Their findings revealed that urban adolescents scored significantly higher than rural adolescents across all dimensions of creative tendency (*p* < 0.05). Li and Hu (2016) conducted research on 8,257 undergraduate students at an engineering university in Beijing and similarly found that urban students exhibited significantly higher total creative tendency scores than rural students (*p* < 0.05).

Other studies suggest that no significant differences exist in creative tendency between urban and rural students. Jiao (2023) administered the WCAP to 192 students and found no significant differences between rural and urban students on the risk-taking and curiosity dimensions (*p* > 0.05). Dai and Xu (2010) surveyed 200 university students using the Williams Creativity Assessment Packet and found no significant differences in total creative tendency scores between rural and urban students.

#### 1.3.2 Grade level

Grade level may moderate creative tendency scores among Chinese students. Some studies suggest that higher-grade students score higher than lower-grade students. Cai and Zhu (2007) surveyed 524 students from fifth grade, eighth grade, and eleventh grade. Their findings revealed that higher-grade students generally scored higher on creative tendency than lower-grade students. Guo et al. (2018) employed the Williams Creativity Assessment Packet revised by Lin and Wang to survey 1,146 undergraduate students from freshman through junior year. The results showed significant differences in creative tendency across grade levels, with total creative tendency scores increasing as grade level advanced.

Other studies indicate that higher-grade students score lower than lower-grade students. Li and Hu (2016) used the Williams Creativity Assessment Packet revised by Lin and Wang to survey all 8,257 undergraduate students at an engineering university in Beijing. They found significant differences in creative tendency scores across grade levels (*p* < 0.05), with creativity tendency scores gradually decreasing as grade level increased. Li et al. (2005) administered the Williams Creativity Assessment Packet revised by Lin and Wang to 595 middle school students. The results revealed significant differences between seventh grade and both eleventh and twelfth grades (*p* < 0.05), with creative tendency scores showing a declining trend as grade level increased.

#### 1.3.3 Gender

Gender may moderate creative tendency scores among Chinese students. Some studies suggest that male students score higher than female students on creativity. Liu (2016) employed the WCAP revised by Lin and Wang to survey 150 high school students at a secondary school. The findings revealed significant gender differences in creative tendency scores, with male students scoring significantly higher than female students on the risk-taking dimension (*p* < 0.05). Zheng et al. (2013) used the WCAP revised by Lin and Wang to survey 478 students from freshman through senior year at Beijing University of Chemical Technology. The results showed that male students‘ mean scores exceeded female students' scores across all creative tendency measures, with particularly significant differences on the curiosity subdimension (*p* < 0.04).

Other studies indicate that male students score lower than female students on creativity. Du and Niu (2013) administered the Williams Creativity Assessment Packet revised by Lin and Wang to 230 elementary school students from fourth through sixth grade. The results showed that female students scored significantly higher than male students on the imagination dimension. Li and Hu (2016) used the Williams Creativity Assessment Packet revised by Lin and Wang to survey 8,257 undergraduate students at an engineering university in Beijing. They found significant gender differences in overall creativity tendency scores, with female students' mean scores significantly higher than male students' scores (*p* < 0.001).

#### 1.3.4 Type of publication

Publication type may moderate the level of creative tendency among Chinese students. Methodological research has found that peer-reviewed journals tend to publish studies with statistically significant results. This selection bias may lead to studies with null findings being shelved, resulting in systematic differences in effect sizes reported between journal articles and dissertations. Preliminary observations indicate that the mean scores reported in dissertations included in this study are generally lower than those reported in journal articles. This suggests that the moderating effect of publication type may also exist (Borenstein et al., 2009; Card, 2012).

### 1.4 The purpose and research questions of the current paper

This meta-analysis aimed to estimate the overall level of creative tendency and its four dimensions among mainland Chinese students and to examine whether the level of creative tendency varies as a function of hometown location, grade level, gender, publication type, and study quality. Accordingly, the following two research questions were addressed:

Q1: what is the overall level of creative tendency and its four dimensions among mainland Chinese students?

Q2: does the level of creative tendency vary as a function of hometown location, grade level, gender, publication type, and study quality?

## 2 Methods

### 2.1 Williams Creativity Assessment Packet

#### 2.1.1 Overview

The Williams Creativity Assessment Packet (WCAP) is an instrument designed to assess individual creative tendency, developed by American psychologist F.E. Williams (Plucker et al., 2010; Williams, 1980) and subsequently revised by Lin and Wang (1994). The scale aims to comprehensively evaluate an individual's level of creativity by measuring their performance across four dimensions: risk-taking, curiosity, imagination, and challenge, through a series of questions and tasks.

The scale comprises four primary dimensions:

*Risk-taking*. This dimension reflects the courage to face failure or criticism and the willingness to function in unstructured environments (Williams, 1980). For example, it assesses whether individuals are willing to try new games or challenge conventions.

*Curiosity*. This dimension captures the desire to explore, the enjoyment of pondering ideas, and openness to puzzling situations (Williams, 1980). For example, it examines whether individuals enjoy observing new things or investigating answers to questions.

*Imagination*. This dimension measures the ability to construct mental images and to intuitively perceive beyond sensory or reality boundaries (Williams, 1980). For example, it evaluates whether individuals enjoy imagining themselves as artists or creating new things.

*Challenge*. This dimension represents the desire to accept challenges, seek multiple alternatives, perceive gaps between current and possible states, and derive order from chaos (Williams, 1980). For example, it assesses whether individuals enjoy solving difficult problems or proposing new ideas.

The WCAP contains 50 items rated on a 3-point scale: “does not apply at all” (1 point), “partially applies” (2 points), and “completely applies” (3 points). For positively worded items, higher scores indicate stronger creative tendency; for negatively worded items, lower scores indicate stronger creative tendency. The final score is calculated by summing scores across the four dimensions, with higher total scores indicating higher levels of creativity.

#### 2.1.2 Reliability and validity

The WCAP demonstrates acceptable reliability and validity when applied to Chinese student populations.

Existing research examined the reliability of the WCAP among Chinese students. Based on a large sample Taiwan students, Lin and Wang (1994) reported the internal consistency reliability for the total scale ranged from 0.765 to 0.877, with test-retest reliability ranging from 0.489 to 0.810. In mainland China, Liu et al. (2016) reported Cronbach's α coefficients of 0.525 to 0.884 for the total scale and its dimensions, with test-retest reliability ranging from 0.593 to 0.702. Fan et al. (2022) further found that both Cronbach's α and McDonald's ω for the total scale were 0.90, with dimension-level α coefficients ranging from 0.62 to 0.78 and ω coefficients from 0.61 to 0.77. Overall, the WCAP's reliability is acceptable.

Prior research also tested the WCAP's validity from a structural perspective. Liu et al. (2016) found that correlations between each dimension and the total scale exceeded 0.70, supporting the structural soundness of the scale. Fan et al. (2022) further reported that inter-dimensional correlations ranged from 0.47 to 0.65, indicating that dimensions are related yet relatively independent. Based on those data, the WCAP's validity is also acceptable.

### 2.2 Search for primary studies

To identify potential primary studies, we conducted literature searches across Chinese and English databases, including China National Knowledge Infrastructure (CNKI), Wanfang, Chongqing VIP, Taylor and Francis, SAGE, Elsevier, John Wiley, Springer, PsycINFO, Education Resources Information Center(ERIC), JSTOR, and ProQuest Dissertations and Theses Global.

Search terms included: “威廉斯创造性倾向量表” (or “Williams' Creativity Assessment Packet”); “创造性” (or “creativity”); “学生” (or “student”); and “Williams.” The complete database- and platform-specific search strategies and search fields are presented in Table 1.

**Table 1:** Search strategies for each database and platform.

Database or platform	Search strategies	Search fields
China National Knowledge Infrastructure (CNKI)	(“威廉斯创造性倾向量表” OR “威廉斯创造力倾向量表” OR “Williams 创造性倾向量表” OR “Williams 创造力倾向量表”) AND (“创造性” OR “创造力” OR “创造倾向”) AND (“学生” OR “小学生” OR “中学生” OR “大学生”)	Subject/topic (title, abstract, and keywords)
Wanfang Data	(“威廉斯创造性倾向量表” OR “威廉斯创造力倾向量表” OR “Williams 创造性倾向量表” OR “Williams 创造力倾向量表”) AND (“创造性” OR “创造力” OR “创造倾向”) AND (“学生” OR “小学生” OR “中学生” OR “大学生”)	Subject/topic (title, abstract, and keywords)
Chongqing VIP Database	(“威廉斯创造性倾向量表” OR “威廉斯创造力倾向量表” OR “Williams 创造性倾向量表” OR “Williams 创造力倾向量表”) AND (“创造性” OR “创造力” OR “创造倾向”) AND (“学生” OR “小学生” OR “中学生” OR “大学生”)	Subject/topic (title, abstract, and keywords)
Taylor & Francis Online	(“Williams Creativity Assessment Packet” OR “Williams Creative Tendency Scale” OR “Williams creativity test” OR WCAP) AND (creativity OR “creative tendency” OR “creative disposition”) AND (student^*^ OR pupil^*^ OR undergraduate^*^) AND (China OR Chinese)	Title, abstract, and keywords
SAGE Journals	(“Williams Creativity Assessment Packet” OR “Williams Creative Tendency Scale” OR “Williams creativity test” OR WCAP) AND (creativity OR “creative tendency” OR “creative disposition”) AND (student^*^ OR pupil^*^ OR undergraduate^*^) AND (China OR Chinese)	Title, abstract, and keywords
ScienceDirect (Elsevier)	(“Williams Creativity Assessment Packet” OR “Williams Creative Tendency Scale” OR “Williams creativity test” OR WCAP) AND (creativity OR “creative tendency” OR “creative disposition”) AND (student^*^ OR pupil^*^ OR undergraduate^*^) AND (China OR Chinese)	Title, abstract, and author-specified keywords
Wiley Online Library	(“Williams Creativity Assessment Packet” OR “Williams Creative Tendency Scale” OR “Williams creativity test” OR WCAP) AND (creativity OR “creative tendency” OR “creative disposition”) AND (student^*^ OR pupil^*^ OR undergraduate^*^) AND (China OR Chinese)	Title, abstract, and keywords
SpringerLink	(“Williams Creativity Assessment Packet” OR “Williams Creative Tendency Scale” OR “Williams creativity test” OR WCAP) AND (creativity OR “creative tendency” OR “creative disposition”) AND (student^*^ OR pupil^*^ OR undergraduate^*^) AND (China OR Chinese)	Title, abstract, and keywords
PsycINFO	(“Williams Creativity Assessment Packet” OR “Williams Creative Tendency Scale” OR “Williams creativity test” OR WCAP) AND (creativity OR “creative tendency” OR “creative disposition”) AND (student^*^ OR pupil^*^ OR undergraduate^*^) AND (China OR Chinese)	Title, abstract, keywords, and subject terms
Education Resources Information Center (ERIC)	(“Williams Creativity Assessment Packet” OR “Williams Creative Tendency Scale” OR “Williams creativity test” OR WCAP) AND (creativity OR “creative tendency” OR “creative disposition”) AND (student^*^ OR pupil^*^ OR undergraduate^*^) AND (China OR Chinese)	Title, abstract, keywords, and subject terms
JSTOR	(“Williams Creativity Assessment Packet” OR “Williams Creative Tendency Scale” OR “Williams creativity test” OR WCAP) AND (creativity OR “creative tendency” OR “creative disposition”) AND (student^*^ OR pupil^*^ OR undergraduate^*^) AND (China OR Chinese)	Title, abstract, and full text
ProQuest Dissertations & Theses Global	(“Williams Creativity Assessment Packet” OR “Williams Creative Tendency Scale” OR “Williams creativity test” OR WCAP) AND (creativity OR “creative tendency” OR “creative disposition”) AND (student^*^ OR pupil^*^ OR undergraduate^*^) AND (China OR Chinese)	Title, abstract, and subject terms

The English-language search, conducted through February 12, 2025, yielded 65 primary studies, while the Chinese-language search, conducted through February 16, 2025, yielded 808 primary studies. By reviewing titles, abstracts, and keywords, we identified 252 primary studies as potentially eligible for inclusion. After full-text screening, 211 studies comprising 211 independent samples were ultimately included in the meta-analysis. Figure 1 provides a visual representation of our search process (Page et al., 2021).

**Figure 1:**
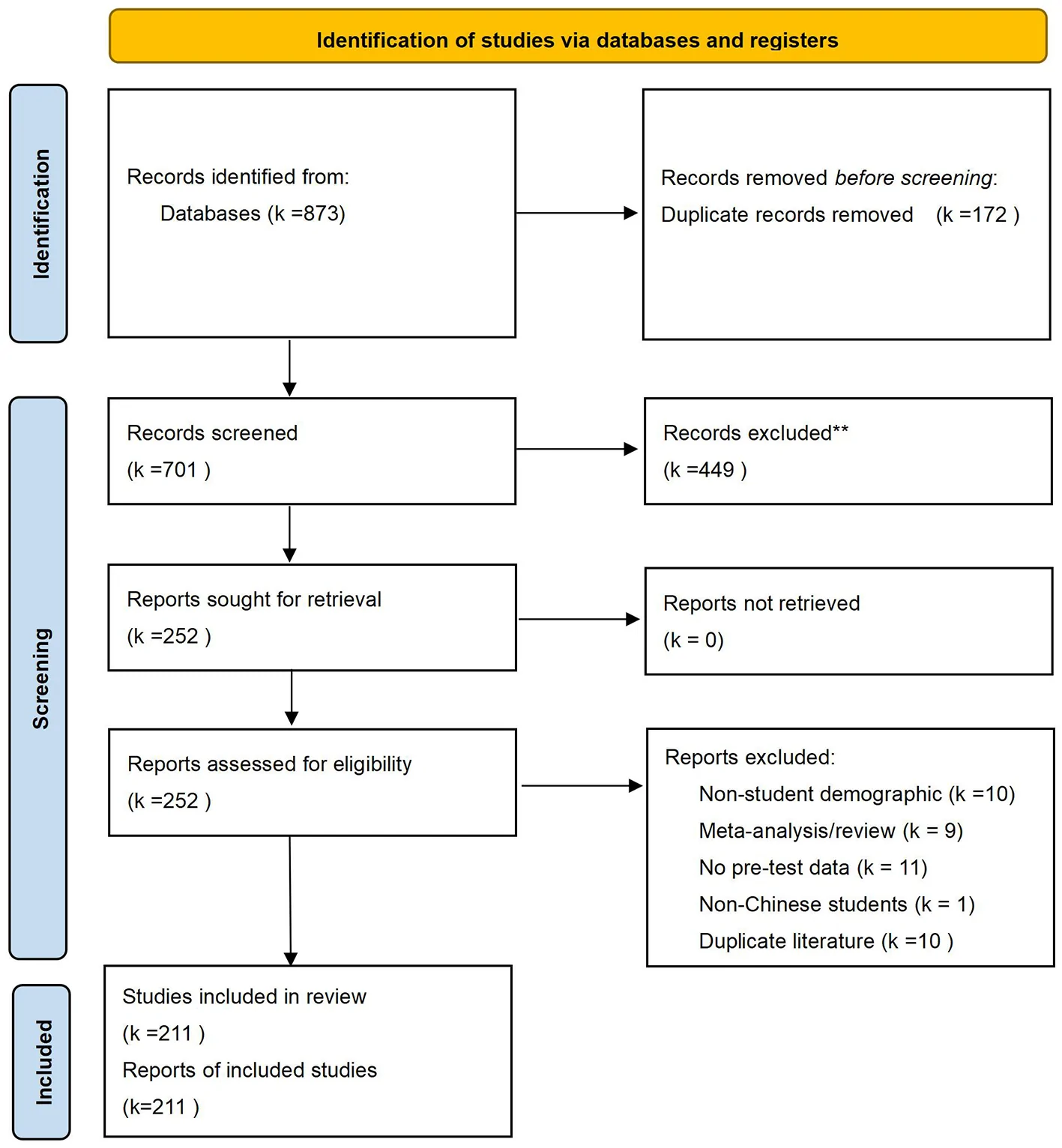
The process of literature searching. Figure 1 was adapted from the original PRISMA 2020 flow diagram template. Licensed under Creative Commons Attribution 4.0 International (CC BY 4.0). Source: https://www.prisma-statement.org/prisma-2020-flow-diagram (Page et al., 2021). ^**^All 449 records were excluded manually during title and abstract screening; no automation tools were used.

### 2.3 Inclusion and exclusion criteria

The inclusion criteria were as follows: (1) Participants were mainland Chinese students who were physically healthy, with no major physical or psychological disorders. (2) The measurement instrument used was the WCAP, which includes four dimensions. (3) Primary studies reported sample size, mean, and standard deviation. (4) When multiple publications by the same author(s) drew from the same dataset, only one article was selected for inclusion in the meta-analysis (Figure 1).

### 2.4 Coding process and coding quality

Coding was conducted by two authors of the article. The development of a coding plan marked the beginning of the coding process, during which we identified the necessary information for further computations. The study features (i.e. moderators) and other information extracted were listed in Table 2 and Supplementary Table S1. The next step was to extract information from each primary study according to the coding plan. The coder WY initially coded five primary studies and reviewed the coding plan. Then a formal coding table was created, and all primary studies were coded accordingly (see Supplementary Table S1 for details). FHY subsequently verified all entries in the coding table against the corresponding primary studies. The initial verification agreement was 98%, calculated as the number of entries confirmed without correction divided by the total number of entries checked and multiplied by 100. Any discrepancies were resolved through discussion between the two coders and, when necessary, consultation with a meta-analysis methodological expert. Following this process, consensus was reached on all final coding decisions.

**Table 2:** Study features extracted in the primary studies.

Moderators	Primary studies		Independent samples	
	*k*	%	*k_1_*	%
Urban areas	18	50.00%	15	51.72%
Rural areas	18	50.00%	14	48.28%
Elementary school	17	21.52%	25	15.15%
Junior middle school	17	21.52%	29	17.58%
Senior high school	18	22.78%	33	20.00%
Undergraduate school	27	34.17%	78	47.27%
Male	83	50.00%	62	50.00%
Female	83	50.00%	62	50.00%
General journals	51	24.40%	30	18.52%
Core Journals	28	13.40%	14	8.64%
Dissertations	130	62.20%	118	72.84%

*k* denotes the number of primary studies reporting each category; *k*_1_ denotes the number of independent samples reported for that category.

This study complies with the Transparency and Openness Promotion (TOP) Guidelines adopted by PSPR. The meta-analytic dataset, R analysis code and related materials are publicly available via the Open Science Framework (OSF) at https://osf.io/gyjm4?view_only=5fca3473bc1845e9b8d4a410d56d1887.

### 2.5 Effect size indicator

The present study employed the mean *(M)* as the effect size indicator (Borenstein et al., 2009; Card, 2012). When the mean is used as the outcome measure, study means and their sampling variances can be synthesized using the general inverse-variance approach applied to other outcome measures, such as *r* and *d* (Borenstein et al., 2009). To facilitate comparison, the mean for each dimension was defined as the total score for that dimension divided by the number of items in that dimension. The mean for the total scale was calculated by dividing the total scale score by the total number of items.

For example, in Shi and Xu (2009) study, to enable standardized comparison, the researcher transformed scores for dimensions such as risk-taking. Specifically, the risk-taking dimension contained 11 items, and the total score across all participants for this dimension was 23.92. The researcher divided the total score by the number of items (23.92 ÷ 11 = 2.17), yielding a mean score of 2.17 per item, thereby obtaining a standardized score for comparison. The corresponding standard deviation was likewise divided by 11, so that both the mean and standard deviation were expressed on the same per-item scale.

### 2.6 Model selection

This study employed a random-effects model. The primary distinction between random-effects and fixed-effect models lies in the sources of error. The fixed-effect model assumes that each study included in the meta-analysis shares the same true effect size, with differences in effect sizes attributable solely to random error. In contrast, the random-effects model assumes that different studies possess different true effect values, with differences in effect sizes resulting from both random error and systematic error (Borenstein et al., 2009; Card, 2012; Harrer et al., 2021).

The choice between random-effects and fixed-effect models depends primarily on the researcher's objectives (Borenstein et al., 2009), specifically whether the researcher intends to generalize the findings and draw broad conclusions. The fixed-effect model does not permit generalization and can only reflect the characteristics of the included primary studies. The random-effects model, however, treats the included primary studies as a sample and attempts to generalize findings to broader contexts (Borenstein et al., 2009; Hedges and Vevea, 1998; Riley et al., 2011). We used inverse-variance weighting, estimated between-study variance by REML, and calculated 95% confidence intervals for pooled means using the Hartung–Knapp adjustment (Knapp and Hartung, 2003).

### 2.7 Homogeneity test

Heterogeneity testing primarily involves the *Q*-test and *I*^2^statistic. The *Q* statistic represents the weighted sum of squared deviations of effect sizes and describes the ratio of observed variation to within-study error (Borenstein et al., 2009; Card, 2012; Harrer et al., 2021). *I*^2^reflects the proportion of total variation in effect sizes attributable to heterogeneity. For the *Q*-test, *p* < 0.05 indicates the presence of heterogeneity. *I*^2^values of 25%, 50%, and 75% indicate low, moderate, and high levels of heterogeneity, respectively (Borenstein et al., 2009; Card, 2012; Harrer et al., 2021).

### 2.8 Detection of publication bias

Publication bias refers to the phenomenon where studies with statistically significant results are more likely to be publication, while those with nonsignificant results are less likely to appear in the published literature (Borenstein et al., 2009; Card, 2012; Harrer et al., 2021). To examine potential publication bias in this meta-analysis, we employed four methods: Fail-safe *N* (Nfs; Rosenthal, 1979), funnel plot inspection (Egger et al., 1997; Sterne and Egger, 2001), trim-and-fill method (Duval and Tweedie, 2000), and PET-PEESE analysis (Stanley and Doucouliagos, 2014).

### 2.9 Assessment of quality

The quality of the primary studies included in this meta-analysis was assessed using the Basic Quality Assessment of Primary Study (BQAPS) (Xu et al., 2024), which was developed based on the Cochrane Risk of Bias framework (Higgins et al., 2011). The BQAPS evaluates core methodological aspects of primary studies, including research design, measurement reliability and validity, statistical methods, and implementation procedures.

The BQAPS consists of 12 items, each rated on a three-point ordinal scale: 0 (insufficient information for judgment), 1 (does not meet methodological requirements), and 2 (adequate information and meets methodological requirements). Higher scores indicate lower risk of bias. The total BQAPS score ranges from 0 to 24 and was used both for categorical classification and as a continuous indicator of study quality.

Based on the total scores, studies were categorized as low quality (0–6), low-medium quality (7–12), medium-high quality (13–18), and high quality (19–24). To enhance transparency and reproducibility, a detailed description of the BQAPS criteria and an example of item-level scoring are provided in the Supplementary Materials (Xu et al., 2024).

The BQAPS total score was treated as a quantitative moderator because it provides an ordered composite measure of overall methodological quality. Treating the total score as a quantitative variable preserves the information contained in the full score range, whereas the quality categories were used only to describe the distribution of study quality.

### 2.10 Statistical calculation tools

This study employed the Q-test statistic for analysis. Pairwise grade-level differences were tested using independent-samples *t* tests, and Cohen's *d* was calculated using the pooled standard deviation of the two grade groups (Cohen, 1988). Because several prespecified moderators were examined across multiple outcomes, the omnibus tests were treated as the primary results, and the individual comparisons were interpreted cautiously in view of potential multiplicity. All statistical analyses were conducted using R software (R Core Team, 2023). Meta-regression analyses were performed using the meta package (Schwarzer, 2025), random-effects models were calculated using the metafor package (Viechtbauer, 2010), and funnel plots were generated using the meta package (Balduzzi et al., 2019).

### 2.11 Sensitivity analyses

Sensitivity analyses were conducted separately for the total creative tendency score and the four dimensions of risk-taking, curiosity, imagination, and challenge-seeking. First, leave-one-out analyses were performed by sequentially omitting each study and recalculating the pooled mean to determine whether the overall estimate was disproportionately affected by any individual study. Second, externally studentized residuals and influence diagnostics were examined (Harrer et al., 2021; Viechtbauer and Cheung, 2010). Studies with absolute studentized residuals greater than 1.96 were classified as potential outliers, and Cook's distance greater than 4/*k* was used to identify potentially influential studies. The random-effects models were subsequently re-estimated after excluding potential outliers. Finally, the robustness of the results to alternative statistical specifications was examined by comparing the restricted maximum likelihood (REML), DerSimonian–Laird (DL), Paule–Mandel (PM), and Sidik–Jonkman (SJ) estimators of between-study variance. The Hartung–Knapp adjustment was used for the confidence intervals in the primary random-effects analyses (Knapp and Hartung, 2003).

## 3 Results

### 3.1 Characteristics of primary studies included

Among the 252 primary studies, 211 met the inclusion criteria for this study. These 211 primary studies reported 211 independent samples with a cumulative total sample size of 130,642. The primary studies were published between 1999 and 2025.

The included studies covered students from different hometown locations and educational stages. For hometown location, 15 independent samples comprised students from urban areas (*N* = 22,583), and 14 comprised students from rural areas (*N* = 21,881). For grade level, the available data included 25 independent samples of elementary school students (*N* = 3,849), 29 of junior middle school students (*N* = 4,409), 33 of senior high school students (*N* = 4,793), and 78 of university students (*N* = 19,762). Table 2 presents the numbers of primary studies and independent samples for each category. The detailed characteristics of all primary studies included in the meta-analysis are presented in Table 3.

**Table 3:** Characteristics of the primary studies included in the meta-analysis.

Study ID	Publication year	Valid N	Publication type
An (2023)	2023	105	Dissertation
An (2011)	2011	108	Dissertation
Cai (2018)	2018	236	Journal article
Cai et al. (2001)	2001	121	Journal article
Cai (2010)	2010	400	Dissertation
Cai (2017)	2017	200	Dissertation
Cai and Zhu (2007)	2007	524	Journal article
Cao (2021)	2021	967	Dissertation
Chai and Han (2008)	2008	200	Journal article
Chen and Zhang (2021)	2021	196	Journal article
Chen et al. (2020)	2020	420	Journal article
Chen (2018)	2018	139	Journal article
Chen (2019)	2019	293	Dissertation
Chen and Zhu (2010)	2010	469	Journal article
Chen and Wang (2021)	2021	2,238	Journal article
Chen N. (2020)	2020	530	Dissertation
Chen (2013)	2013	360	Dissertation
Chen et al. (2011)	2011	250	Journal article
Chen Y. H. (2020)	2020	247	Dissertation
Chen (2014)	2014	48	Journal article
Chen (2022)	2022	100	Dissertation
Chen Z. F. (2020)	2020	995	Dissertation
Cheng (2023)	2023	811	Dissertation
Chu (2024)	2024	60	Dissertation
Dai and Xu (2010)	2010	200	Journal article
Dai et al. (2024)	2024	1,047	Journal article
Deng (2013)	2013	34	Dissertation
Du (2009)	2009	262	Dissertation
Du and Niu (2013)	2013	230	Journal article
Du (2022)	2022	40	Dissertation
Fang (2013)	2013	490	Dissertation
Feng (2021)	2021	46	Dissertation
Fu (2013)	2013	120	Dissertation
Fu (2023)	2023	98	Dissertation
Gai (2021)	2021	236	Dissertation
Gao (2023)	2023	700	Dissertation
Gao and Zeng (2012)	2012	1,139	Journal article
Gou (2021)	2021	60	Dissertation
Group Research (1999)	1999	100	Journal article
Guo et al. (2018)	2018	1,146	Journal article
Guo (2017)	2017	24,446	Journal article
Guo (2018)	2018	294	Dissertation
Han (2012)	2012	374	Dissertation
He (2019)	2019	2,428	Dissertation
Hu et al. (2017)	2017	30	Journal article
Huang (2024)	2024	107	Dissertation
Huang (2018)	2018	320	Dissertation
Huang (2023)	2023	83	Dissertation
Huang (2010)	2010	85	Dissertation
Huang (2022)	2022	42	Dissertation
Ji (2019)	2019	969	Dissertation
Ji et al. (2019)	2019	414	Journal article
Ji (2007)	2007	818	Dissertation
Jia (2009)	2009	92	Dissertation
Jia (2017)	2017	97	Dissertation
Jiang (2016)	2016	40	Journal article
Jiang (2021)	2021	20	Dissertation
Jiao (2023)	2023	192	Journal article
Kaifeng Yishi Fuxiao Research Group (1999)	1999	212	Journal article
Kong (2016)	2016	178	Dissertation
Lai (2019)	2019	55	Dissertation
Lan et al. (2019)	2019	105	Journal article
Li and He (2023)	2023	300	Journal article
Li D. Y. (2021)	2021	72	Dissertation
Li F. G. (2009)	2009	98	Dissertation
Li (2015)	2015	2,985	Dissertation
Li and Hu (2016)	2016	8,257	Journal article
Li W. (2009)	2009	431	Journal article
Li et al. (2005)	2005	595	Journal article
Li Y. F. (2021)	2021	186	Dissertation
Li et al. (2012)	2012	465	Journal article
Liang (2024)	2024	128	Dissertation
Liang and Shen (2021)	2021	5,248	Journal article
Liang (2014)	2014	123	Dissertation
Liang (2021)	2021	64	Dissertation
Liang (2019)	2019	41	Dissertation
Lin (2006)	2006	96	Dissertation
Liu et al. (2013)	2013	306	Journal article
Liu et al. (2014)	2014	126	Journal article
Liu (2013)	2013	193	Dissertation
Liu (2014)	2014	435	Dissertation
Liu F. C. (2020)	2020	43	Dissertation
Liu H. (2023)	2023	569	Dissertation
Liu (2007)	2007	100	Dissertation
Liu (2010)	2010	216	Dissertation
Liu M. H. (2020)	2020	1,216	Dissertation
Liu and Zhang (2017)	2017	1,650	Journal article
Liu (2008)	2008	500	Dissertation
Liu S. Q. (2020)	2020	160	Dissertation
Liu W. S. (2023)	2023	41	Dissertation
Liu X. (2011)	2011	276	Dissertation
Liu and Shan (2013)	2013	186	Journal article
Liu (2016)	2016	150	Journal article
Liu et al. (2016)	2016	120	Journal article
Liu Y. (2020)	2020	41	Dissertation
Liu Y. X. (2023)	2023	87	Dissertation
Liu et al. (2011)	2011	187	Journal article
Liu Z. C. (2020)	2020	541	Dissertation
Lou et al. (2017)	2017	60	Journal article
Lu et al. (2022)	2022	21	Journal article
Luo (2020)	2020	142	Dissertation
Luo (2010)	2010	1,008	Journal article
Lv J. (2016)	2016	823	Dissertation
Lv (2024)	2024	108	Dissertation
Lv Y. (2016)	2016	759	Journal article
Ma (2024)	2024	948	Dissertation
Meng L. (2021)	2021	12	Dissertation
Meng Z. W. (2021)	2021	96	Dissertation
Mu (2016)	2016	70	Dissertation
Ni (2022)	2022	39	Dissertation
Nie and Zheng (2005)	2005	3,727	Journal article
Niu (2019)	2019	325	Dissertation
Niu (2021)	2021	30	Dissertation
Niu (2022)	2022	420	Dissertation
Ouyang (2016)	2016	648	Dissertation
Pan et al. (2016)	2016	400	Journal article
Qian (2013)	2013	686	Dissertation
Qian (2007)	2007	187	Journal article
Qian (2009)	2009	89	Dissertation
Qin (2019)	2019	794	Dissertation
Qiu (2020)	2020	24	Dissertation
Ren et al. (2020)	2020	70	Journal article
Shen (2024)	2024	288	Journal article
Sheng (2018)	2018	197	Dissertation
Shi and Xu (2009)	2009	346	Journal article
Shi (2006)	2006	334	Dissertation
Song et al. (2005)	2005	322	Journal article
Su (2024)	2024	92	Dissertation
Su (2010)	2010	223	Dissertation
Sun F. (2021)	2021	257	Dissertation
Sun S. J. (2020)	2020	324	Dissertation
Tan (2024)	2024	390	Dissertation
Tang (2024)	2024	398	Dissertation
Teng (2014)	2014	785	Dissertation
Tian (2022)	2022	946	Dissertation
Tong (2023)	2023	29	Dissertation
Tong (2019)	2019	273	Dissertation
Wang and Shu (2013)	2013	873	Journal article
Wang and Wang (2014)	2014	443	Journal article
Wang et al. (2005)	2005	407	Journal article
Wang (2021)	2021	1,109	Dissertation
Wang et al. (2012)	2012	240	Journal article
Wang et al. (2006)	2006	222	Journal article
Wang P. (2020)	2020	500	Journal article
Wang (2008)	2008	324	Dissertation
Wang and Pang (2023)	2023	82	Journal article
Wang et al. (2009)	2009	473	Dissertation
Wang (2010)	2010	52	Dissertation
Wang (2012)	2012	92	Dissertation
Wang and Zhu (2017)	2017	1,032	Journal article
Wang (2001)	2001	160	Journal article
Wang Y. (2009)	2009	341	Dissertation
Wang (2023)	2023	85	Dissertation
Wang (2024)	2024	44	Dissertation
Wang Y. Y. (2020)	2020	642	Dissertation
Wang Z. Y. (2009)	2009	950	Dissertation
Wei (2022)	2022	84	Dissertation
Wu et al. (2022)	2022	68	Journal article
Wu (2018)	2018	737	Dissertation
Wu (2009)	2009	355	Journal article
Wu and Zhang (2006)	2006	125	Journal article
Wu (2023)	2023	29	Dissertation
Xiao (2017)	2017	385	Dissertation
Xie (2021)	2021	66	Dissertation
Xiong (2021)	2021	35	Dissertation
Xu (2014)	2014	261	Journal article
Xu et al. (2019)	2019	364	Journal article
Xu (2017)	2017	208	Dissertation
Xu (2023)	2023	340	Dissertation
Xue (2020)	2020	127	Dissertation
Yan (2024)	2024	65	Dissertation
Yang (2020)	2020	373	Dissertation
Yang (2022)	2022	440	Dissertation
Yang (2016)	2016	24	Dissertation
Yang et al. (2013)	2013	1,203	Journal article
Yang et al. (2011)	2011	239	Journal article
Ye (2015)	2015	83	Dissertation
Yi et al. (2014)	2014	246	Journal article
Yin (2020)	2020	401	Dissertation
You (2007)	2007	658	Dissertation
Yu (2016)	2016	522	Dissertation
Yu (2009)	2009	476	Conference proceeding
Yu and Qian (2010)	2010	311	Conference proceeding
Zeng (2015)	2015	286	Dissertation
Zhai (2019)	2019	93	Dissertation
Zhang et al. (2008)	2008	84	Journal article
Zhang et al. (2018)	2018	315	Journal article
Zhang et al. (2014)	2014	145	Journal article
Zhang (2004)	2004	469	Dissertation
Zhang et al. (2012)	2012	550	Journal article
Zhang (2017)	2017	200	Dissertation
Zhang (2020)	2020	16,635	Dissertation
Zhang et al. (2022)	2022	523	Journal article
Zhang (2021)	2021	200	Dissertation
Zhang et al. (1999)	1999	100	Journal article
Zhang and Xu (2011)	2011	237	Journal article
Zhang et al. (2023)	2023	152	Journal article
Zhao (2020)	2020	160	Dissertation
Zhao X. M. (2016)	2016	59	Dissertation
Zhao Z. Q. (2016)	2016	34	Dissertation
Zheng (2014)	2014	94	Dissertation
Zheng and Zhang (2022)	2022	424	Dissertation
Zheng et al. (2013)	2013	454	Journal article
Zhong (2020)	2020	94	Dissertation
Zhou (2021)	2021	63	Journal article
Zhu (2021)	2021	56	Dissertation
Zhu and Song (2016)	2016	139	Journal article
Zhu and Wang (2017)	2017	1,027	Journal article
Zhu (2001)	2001	368	Journal article
Zhu and Wang (2017)	2017	1,032	Journal article
Zou et al. (2019)	2019	65	Dissertation

### 3.2 Results of heterogeneity tests

The *Q*-test value for the total creative tendency score was 326,057.76, *p* < 0.001, while the *Q*-test values for the four dimensions ranged from 196,163.90 to 325,464.85, all with *p* < 0.001. The *I*^2^ value for the total creative tendency score was 99.95%, and the *I*^2^ values for the four dimensions ranged from 99.90% to 99.94%. These results indicate considerable between-study heterogeneity (Table 4). Table 4 also reports τ^2^, τ, and 95% prediction intervals for the total score and each dimension. The wide prediction intervals indicate substantial variation among the true means of the included student samples. The differences in means may reflect substantive variation among student populations, methodological differences among studies, and random sampling variation. We therefore used random-effects meta-regression to examine whether the prespecified study characteristics accounted for part of the between-study variation.

**Table 4:** Heterogeneity results and main effects.

Scale	**k*_1_*	*M*	*SE*	*95%* CI	**I* ^2^*	*Q*	*τ^2^*	τ	*95%* PI
Total score	162	2.158	0.017	2.124, 2.192	99.95%	326057.76^***^	0.047	0.218	1.727, 2.589
Risky	193	2.135	0.018	2.101, 2.170	99.93%	270276.00^***^	0.058	0.241	1.658, 2.613
Curiosity	194	2.150	0.021	2.108, 2.191	99.93%	294524.17^***^	0.084	0.290	1.577, 2.723
Imaginative	193	2.060	0.016	2.029, 2.091	99.90%	196163.90^***^	0.046	0.215	1.634, 2.486
Challenging	194	2.187	0.019	2.149, 2.225	99.94%	325464.85^***^	0.071	0.267	1.660, 2.714

*k*_1_ denotes the number of independent samples contributing data to the pooled estimate for each scale; *M*, pooled mean; *SE*, standard error; CI, confidence interval; *I*^2^, heterogeneity statistic; *Q*, Cochran's heterogeneity statistic; τ^2^, estimated between-study variance; τ, estimated between-study standard deviation; PI, prediction interval. ^***^p < 0.001.

### 3.3 Results of publication tests

Funnel-plot analysis revealed an asymmetric distribution of the study mean estimates, with potentially missing estimates primarily on the left side. Using the trim-and-fill method, 25 potentially missing estimates were imputed. The pooled mean was 2.158 (95% CI [2.124, 2.192]) before adjustment and 2.125 (95% CI [2.093, 2.157]) after adjustment, a difference of 0.033.

Rosenthal's fail-safe *N* was 346,937,909, far exceeding the conventional threshold of 820 (5*k*_1_ + 10 = 5 × 162 + 10). Conventionally, a fail-safe *N* above this threshold indicates that a large number of unreported null findings would be required to make the pooled result statistically non-significant. However, because the WCAP is scored on a 1–3 scale, a hypothetical mean of zero is not a meaningful reference value. Therefore, the fail-safe *N* was retained as a supplementary statistic but was not used alone to evaluate the robustness of the pooled mean or the absence of publication bias.

In the absence of selection and other small-study effects, less precise study means would be expected to show greater dispersion but remain approximately symmetrical around the pooled mean. Under this assumption, the potentially missing estimates on the left side suggest that small studies reporting lower WCAP means may be underrepresented in the available literature. However, the observed asymmetry may also reflect differences in student populations, grade levels, educational contexts, or study methods. Therefore, the funnel-plot and trim-and-fill results were interpreted cautiously as sensitivity analyses rather than definitive evidence of publication bias (Figure 2).

**Figure 2:**
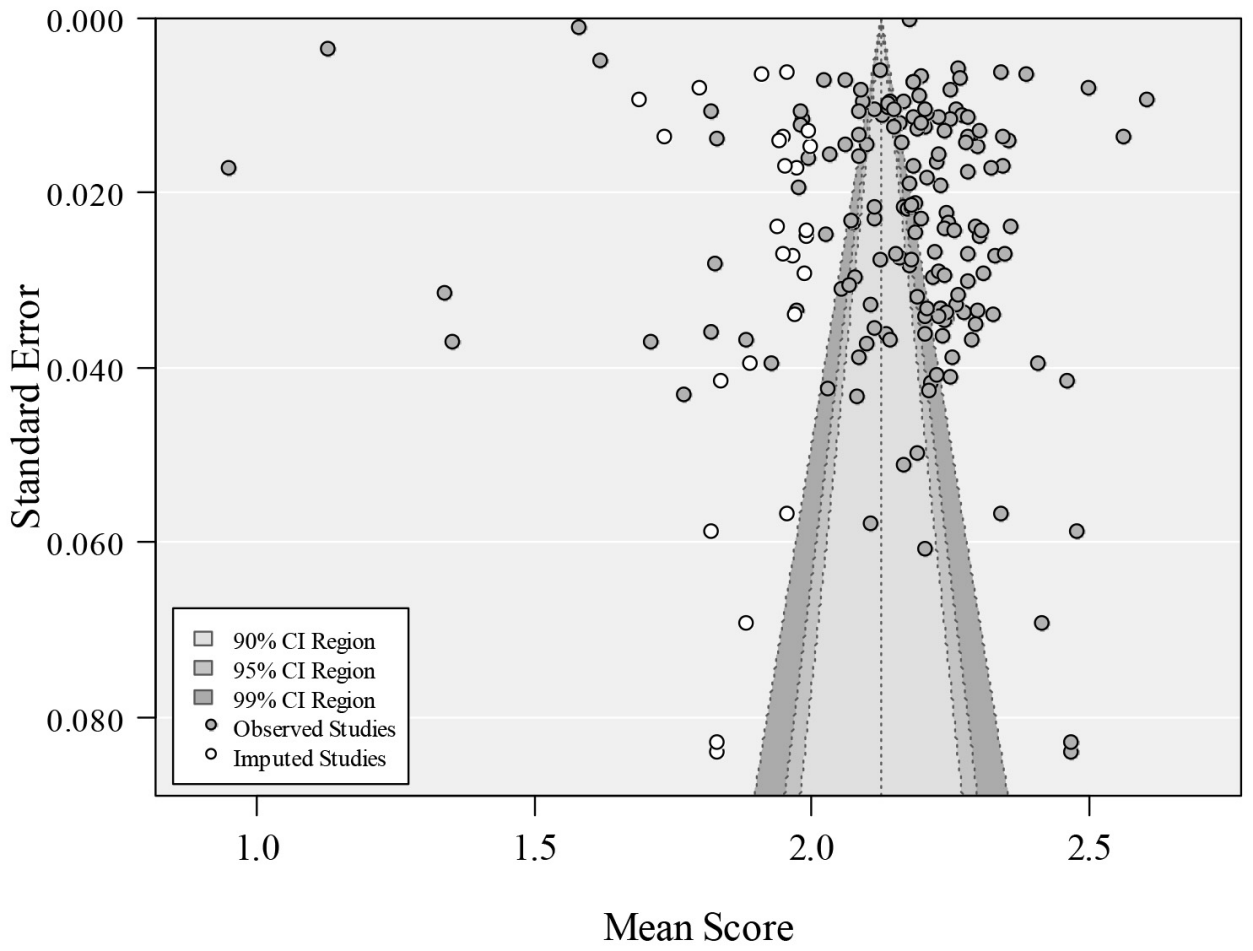
The trimmed funnel plot of the current meta analysis.

PET–PEESE analysis was also conducted to examine possible small-study effects (Carter et al., 2019). The PET regression analysis produced a positive intercept (*b* = 2.109, *p* < 0.001), and the PEESE-adjusted mean was 2.131 (95% CI [2.089, 2.174]), representing a reduction of approximately 1.24% relative to the original estimate. Because zero is not a meaningful reference value for the WCAP raw mean, the significant PET intercept was not interpreted as evidence of a genuine effect. The small changes in the pooled mean following the trim-and-fill and PET–PEESE analyses suggest that the pooled estimate was relatively stable under the assumptions of these sensitivity analyses. Thus, potential publication bias appeared to have a limited influence on the pooled estimate.

### 3.4 The impact of quality of primary studies

Among the 211 primary studies, no studies fell into the low-to-moderate quality category (quality assessment score: 7–12). A total of 60 studies (37.04%) were classified as moderate-to-high quality (score: 13–18), while 102 studies (62.96%) were classified as high quality (score: 19–24).

The random-effects meta-regression showed that the BQAPS total score was not significantly associated with the total WCAP creative tendency score, *b* = 0.001, *SE* = 0.008, 95% CI [−0.014, 0.016], *t*(160) = 0.185, *p* = 0.854. The omnibus model test was also nonsignificant, *F*(1, 160) = 0.034, *p* = 0.854. These results indicate that study quality did not significantly moderate the total WCAP score.

### 3.5 Main effect

#### 3.5.1 The pooled effect size

As shown in Table 4, the mean effect size (*ES*) for the total creativity disposition score among mainland Chinese students was 2.158 (*k*_1_ = 162, 95% CI [2.124, 2.192]). The mean effect size for risk-taking was 2.135 (*k*_1_ = 193, 95% CI [2.101, 2.170]). The mean effect size for curiosity was 2.150 (*k*_1_ = 194, 95% CI [2.108, 2.191]). The mean effect size for imagination was 2.060 (*k*_1_ = 193, 95% CI [2.029, 2.091]). The mean effect size for challenge-seeking was 2.187 (*k*_1_ = 194, 95% CI [2.149, 2.225]).

Compared with the scores of students from Taiwan district (Lin and Wang, 1994), mainland Chinese students had lower scores for overall creative tendency (*d* = – 0.401), risk-taking (*d* = – 0.309), curiosity (*d* = – 0.321), imagination (*d* = – 0.144), and challenge (*d* = – 0.801).

#### 3.5.2 Results of sensitivity analyses

Sensitivity analyses indicated that the pooled estimates were generally robust. In the leave-one-out analyses, the pooled means ranged from 2.155 to 2.165 for the total score, 2.132 to 2.141 for risk-taking, 2.146 to 2.156 for curiosity, 2.056 to 2.065 for imagination, and 2.183 to 2.192 for challenge-seeking. The maximum absolute change ranged from 0.005 to 0.007, indicating that no individual study materially altered any of the pooled estimates.

Residual diagnostics identified 8, 12, 13, 15, and 14 potential outliers for the total score, risk-taking, curiosity, imagination, and challenge-seeking, respectively. After excluding these studies, the corresponding pooled means were 2.190 (95% CI [2.169, 2.210]), 2.167 (95% CI [2.141, 2.193]), 2.199 (95% CI [2.170, 2.228]), 2.085 (95% CI [2.066, 2.104]), and 2.229 (95% CI [2.202, 2.255]). These values represented relative changes of 1.47%, 1.48%, 2.29%, 1.20%, and 1.93%, respectively.

The point estimates obtained using the REML, DL, PM, and SJ estimators were highly consistent. Moreover, the Hartung–Knapp confidence intervals were nearly identical to the confidence intervals obtained in the primary analyses. These findings indicate that the pooled estimates for the total score and all four dimensions were robust to the omission of individual studies, exclusion of potential outliers, and alternative model specifications.

### 3.6 Results of moderator analyses

#### 3.6.1 Effect of hometown location

Based on economic development level, the studies were divided into two subgroups: urban group (*k*_1_= 15) and rural group (*k*_1_ = 14). The results (*Q*_*between*_ = 0.07, *p* > 0.05) indicated no significant differences between these subgroups (Table 5).

**Table 5:** Results of moderator analyses.

Moderators	*k_1_*	*N*	*M*	*SE*	*95% CI*.	*Q_*b*_*
Hometown locations						
Urban areas	15	22583	2.035	0.085	1.853, 2.218	0.07
Rural areas	14	21881	2.002	0.089	1.809, 2.195	
Grades						
Elementary school	25	3849	2.261	0.024	2.211, 2.311	23.25^***^
Junior middle school	29	4409	2.141	0.038	2.064, 2.219	
Senior high school	33	4793	2.115	0.029	2.056, 2.174	
Undergraduate	78	19762	2.142	0.010	2.123, 2.161	
Gender						
Male	62	29138	2.112	0.034	2.044, 2.181	0.02
Female	62	33075	2.105	0.034	2.037, 2.173	
Type of publication						
General journals	30	8375	2.171	0.027	2.115, 2.227	2.20
Core journals	14	6944	2.222	0.046	2.122, 2.322	
Dissertation	118	40335	2.147	0.022	2.104, 2.191	

*k1 denotes the number of independent samples or non-overlapping subgroup samples contributing data to each moderator category*. ^***^p < 0.001.

#### 3.6.2 Effect of grades

Based on the reported grade information, the primary studies were divided into four groups: elementary school (*k*_1_ = 25), middle school (*k*_1_ = 29), high school (*k*_1_ = 33), and university (*k*_1_ = 78) (see Figure 3). The between-group heterogeneity was significant, *Q*_*between*_ = 23.25, *p* < 0.001. As shown in Figure 3, the mean scores were 2.26 for the elementary-school group, 2.14 for the middle-school group, 2.11 for the high-school group, and 2.14 for the university group. For the Imaginative dimension, the between-group heterogeneity was also significant, *Q*_*between*_= 8.45, *p* < 0.05.

**Figure 3:**
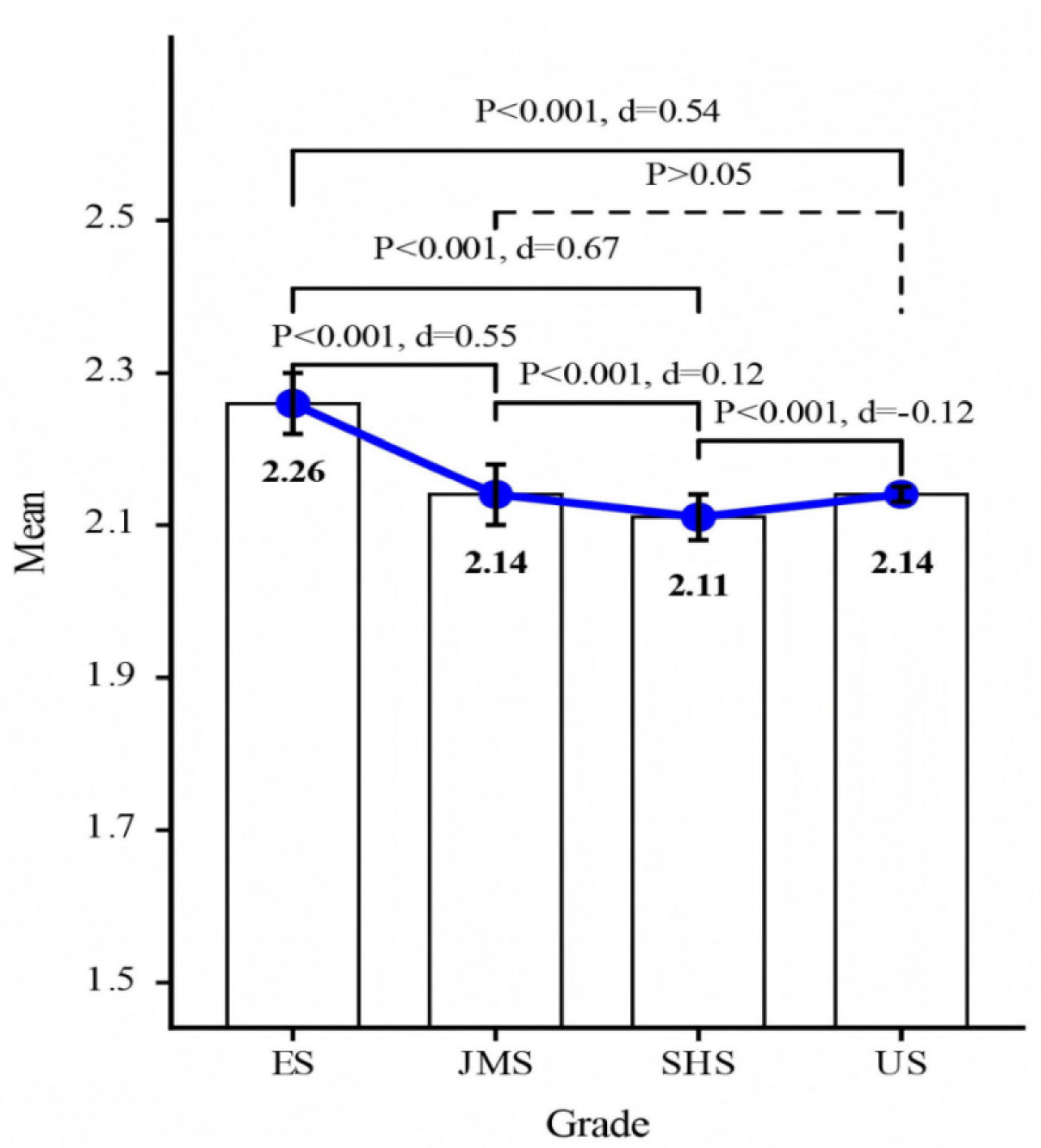
The grade effect on the total creative tendency. ES, Elementary school; JMS, Junior middle school; SHS, Senior high school; US, Undergraduate school.

As shown in Figure 4, imagination scores across grade groups showed a pattern similar to that of the total score. Descriptively, the high-school group had the lowest mean scores for risk-taking, curiosity, and challenge, whereas the university group had means comparable to those of the elementary- and middle-school groups. In contrast, the university group's mean imagination score remained lower than those of the earlier grade groups.

**Figure 4:**
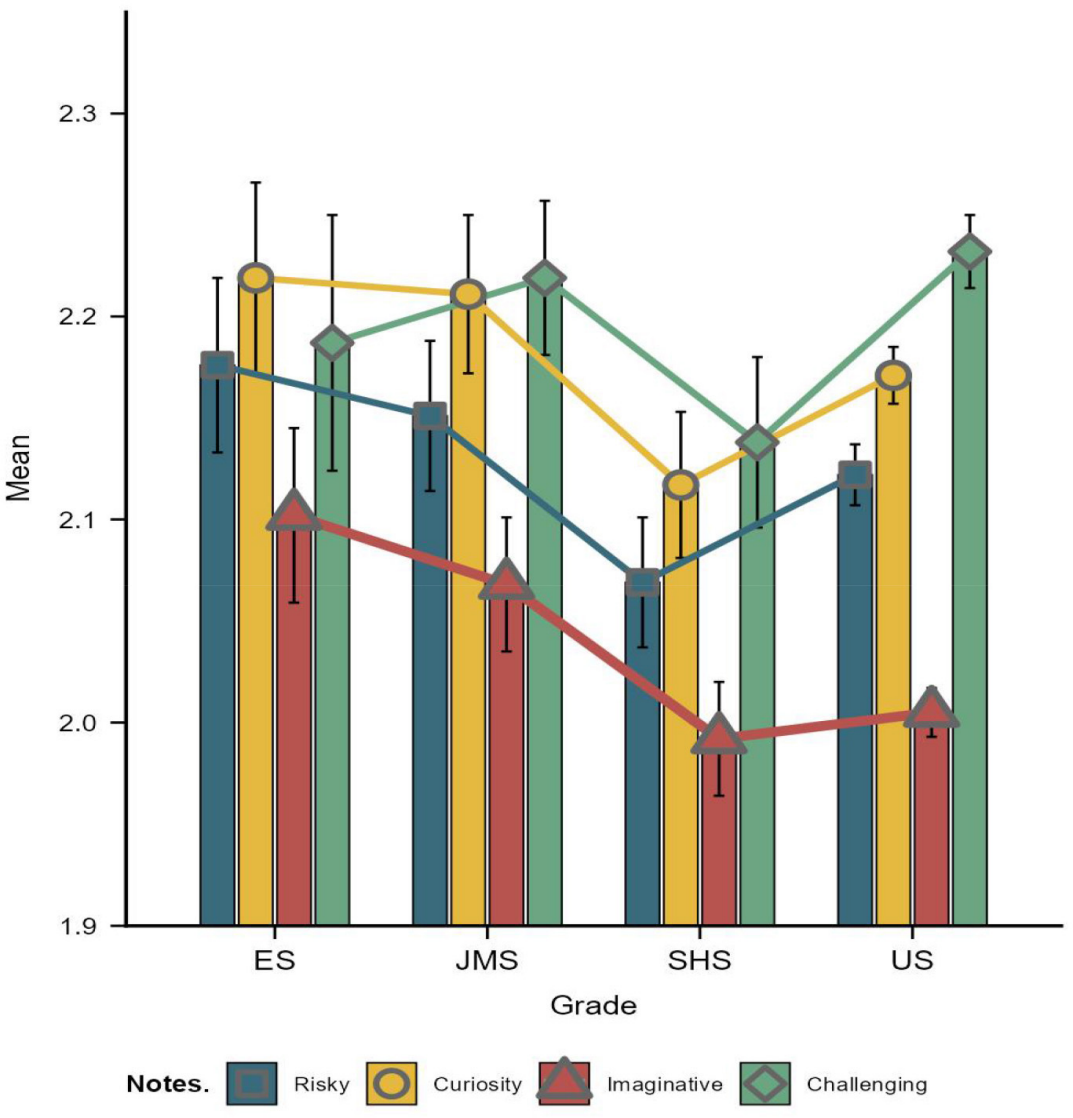
Grade effect on the dimensions of creative tendency. ES, Elementary school; JMS, Junior middle school; SHS, Senior high school; US, Undergraduate school.

#### 3.6.3 Effect of publication type

Based on publication type, the samples were divided into three subgroups: general journals (*k*_1_ = 30), core journals (*k*_1_ = 14), and dissertations (*k*_1_ = 118). The between-group difference was not statistically significant (*Q*_*between*_ = 2.20, *p* = 0.333; Table 5).

#### 3.6.4 Effect of gender

The primary studies were divided into two subgroups: male student group (*k*_1_ = 62) and female student group (*k*_1_ = 62). The results (*Q*_*between*_ = 0.02, *p* > 0.05) indicated no significant differences between these subgroups.

## 4 Discussions

### 4.1 Pooled scores on the WCAP Creative Tendency Scale among mainland Chinese students

This study found a pooled WCAP creative tendency score of 2.158 among mainland Chinese students (*k*_1_ = 162, 95% CI [2.124, 2.192]). This score was numerically lower than the mean of 2.269 reported for students in the Taiwan district (*d* = – 0.401; Lin and Wang, 1994). These results are consistent with Shen (2016) and Zhang (2023), but differ from Shen and Shi's (2010) finding that Chinese secondary-school students showed higher curiosity than several international comparison groups.

Several factors may explain the relatively lower creative tendency scores among Chinese students. First, creativity was given limited emphasis in educational goals, which may have led schools to pay less attention to it in practice. For an extended period, creativity cultivation was not established as a clear core objective within the mainland Chinese education system. In K-12 stage, The 2011 National Curriculum Standards merely called for fostering students' innovative spirit, without establishing creativity development as a key objective of curriculum instruction (Ministry of Education of the People's Republic of China, 2012). It was not until 2022 that the new curriculum standards formally integrated creativity into the core competency framework, explicitly recognizing its critical role in students' holistic development (Ministry of Education of the People's Republic of China, 2022). In higher education, the Ministry of Education required cultivating students' higher-order abilities in 2018 (Ministry of Education of the People's Republic of China, 2018), but failed to declare specific components of these abilities or provide systematic cultivation pathways. This limited emphasis on creativity may be one reason why creative tendency has received less attention in educational practice.

Second, time spent on repetitive learning tasks may leave students with fewer opportunities to explore new ideas. Mainland Chinese students generally face heavy academic burdens, with substantial time devoted to repetitive practice (Pong and Chow, 2002; Gentry, 2006; Zheng, 2013). A meta-analysis by Zhai and Fan (2021) of 187 studies encompassing 188,004 K-12 students revealed that homework time for Chinese students has shown a sustained upward trend: daily homework time increased from 32.36 min to 38.66 min for elementary school students, from 42.18 min to 48.54 min for middle school students, and from 46.79 min to 63.63 min for high school students. Students who spend more time on repetitive practice may have less time for open-ended and creative activities. Upon entering higher education, although the government has introduced policies and promoted the widespread establishment of innovation and entrepreneurship institutes across universities (Pang and Plucker, 2012; General Office of the State Council of the People's Republic of China, 2015), existing innovation and entrepreneurship education still faces significant challenges (Shao et al., 2024; General Office of the State Council of the People's Republic of China, 2015) and may not fully address the limited opportunities for creativity training at earlier educational stages.

Third, cultural expectations may influence whether students feel comfortable expressing new ideas (Ho and Ho, 2008; Schenck and Zhang, 2025; Yao et al., 2010). (i) some traditional Chinese cultural norms may discourage individuals from standing out or proposing novel ideas (as exemplified by the saying “A tree that stands out in the forest will be destroyed by the wind”), which may limit the expression of students' creative tendency (Niu, 2012; Niu and Sternberg, 2002; Yao et al., 2010). An experimental study by Goncalo and Staw (2006) found that collectivism-oriented teams performed significantly worse on creative tasks than individualism-oriented teams. (ii) the Chinese traditional culture emphasis on respecting teachers and valuing their teachings may similarly constrain creativity development. This cultural norm positions teachers as unquestionable authority figures (Kim, 2005, 2007; Lin and Shen, 2024). Yuan and Zhou (2015) suggested that lower-status individuals may be less willing to express ideas that challenge authority, a pattern that may also be relevant in educational settings.

### 4.2 Significant effect of grade

#### 4.2.1 Grade-related differences in creative tendency scores

This study found that grade level demonstrates a significant moderating effect on creative tendency among mainland Chinese students. Mean creative tendency scores were lower in each successive grade group from elementary school to high school and were somewhat higher in the university group. The high-school group had the lowest mean score (Figures 3, 4). This cross-sectional pattern is partially consistent with existing research (Kim, 2011; Said-Metwaly et al., 2021). Said-Metwaly et al. (2021) conducted a meta analysis of 41 studies involving 40,918 participants and found that creativity exhibited significant declines at two critical stages: 7th grade and 10th−11th grade. Kim (2011) conducted a analysis of six large samples with a total of 272,599 participants, spanning from kindergarten through 12th grade and adult populations, and revealed that creative thinking began to decline from 6th grade onward.

Three factors may help explain the differences in scores across grade groups:

First, repetitive learning activities may leave students with fewer opportunities to explore new ideas. To achieve higher scores in the highly competitive high school and university entrance examinations, Chinese students engage in extensive repetitive practice (Zheng, 2013). Behavioral experimental results by Main et al. (2020) showed that repetitive activities reduced cognitive diversity, which may affect creativity development. Additionally, Martindale et al. (1996) assessed creativity in 24 male students using the Remote Associates Test and the Alternate Uses Test, suggesting that repetitive practice may be associated with cognitive habituation and lower creative performance. Repetitive practice may therefore be one factor relevant to the lower scores observed in higher-grade groups.

Second, increasing academic pressure may be associated with physiological stress responses and changes in creative performance. As students advance through school, academic pressure may increase; stress-related responses may involve the noradrenergic system and the hypothalamic–pituitary–adrenal (HPA) axis (Beversdorf, 2018). Entrance examination–related stress may therefore be one possible factor associated with the lower creative tendency scores observed in higher-grade groups (Beversdorf, 2018).

Thirdly, as the college entrance examination (Gaokao) approaches, learning activities may become more predictable and structured, which may limit opportunities for creative exploration. Such increased predictability may be relevant to activity in brain regions associated with curiosity and, in turn, to the expression of creative tendency (Jepma et al., 2012).

The somewhat higher creative tendency scores in the university group may be related to several factors. First, increased opportunities for creativity training may support creativity development. Since the government launched the “Mass Entrepreneurship and Innovation” policy in 2015 (General Office of the State Council of the People's Republic of China, 2015), universities have widely strengthened innovation and entrepreneurship education (Chang et al., 2022; Huang et al., 2021; Shao et al., 2024). A meta-analysis by Guo et al. (2025) encompassing 29 studies and 109 effect sizes demonstrated that creativity training programs significantly enhance university students' creative performance. These educational initiatives may provide university students with creativity training opportunities that support their creativity development (Hu, 2018; Qian, 2018).

Second, increased autonomy among university students may facilitate creativity development. Compared to K-12 education, China universities offer greater flexibility in curriculum design, instructional methods, and assessment systems (Pascarella and Terenzini, 2005), providing students with more autonomous learning space. Cullen and Oppenheimer (2024) found that university students with greater learning autonomy demonstrated higher levels of classroom engagement and learning outcomes. Liu et al. (2023) employed a correlational research design to study 193 middle school students participating in an autonomous 3D design course, finding a significant positive correlation between autonomous learning behaviors and creative performance. According to Deci and Ryan's self-determination theory, autonomy represents one of the basic psychological needs, and satisfying these needs promotes intrinsic motivation and creative performance (Deci and Ryan, 2013). Greater autonomy during the university stage may provide favorable conditions for creativity development and may be relevant to the somewhat higher scores in this group.

#### 4.2.2 Grade-related differences in imagination scores

Contrary to popular belief (e.g., Zheng, 2013), this study revealed that grade level demonstrated a significant moderating effect on the imagination dimension (*Q*_between_ = 8.45, *p* < 0.05), with lower mean scores observed in the higher-grade groups. In contrast, grade-level effects were not significant for the three dimensions of risk-taking, curiosity, and challenge. This finding is largely consistent with Li et al. (2005). Li surveyed 595 middle school students using the same scale and found a significant grade-level decline in the imagination dimension while other dimensions showed no significant differences. However, the present findings are inconsistent with Engelhard and Monsaas (1988) which reported that curiosity declines with grade level.

Physiological factors may also be relevant to the grade-related differences in imagination scores. Increasing homework time may leave students with fewer opportunities for rest (Zhai and Fan, 2021). Previous research has also discussed possible links among rest, default mode network activity, and imagination (Luo et al., 2024; Bartoli et al., 2024). Taken together, heavier learning demands and fewer opportunities for rest may be associated with reduced DMN activity, which may in turn be relevant to imagination scores, a dimension of creative tendency.

### 4.3 Insignificant effect of gender

This study found that gender did not demonstrate a significant moderating effect on creative tendency. This result is consistent with the majority of existing research findings (Abraham, 2016; Baer and Kaufman, 2008; Cheung and Lau, 2010). This phenomenon may be related to the effective implementation of gender equality policies in China education system. China gender equality education policies have created equal opportunities for the creativity development of both male and female students. The Program for the Development of Chinese Women (2021–2030) explicitly proposes strategic goals to fully implement gender equality principles and promote equal access to education at all levels for both genders (State Council of the People's Republic of China, 2021). The China Compulsory Education Law (revised in 2018) and the China Education Law (revised in 2015) legally guarantee equal educational rights for male and female students (National People's Congress of the People's Republic of China, 2015, 2018). The implementation of these policies may have supported more equal opportunities for male and female students in curriculum design, instructional methods, educational resource allocation, and assessment standards, providing a possible foundation for the development of creative tendency. In this increasingly equitable educational context, the present analysis did not find a statistically significant gender difference in creative tendency.

### 4.4 Insignificant effect of hometown location

This study found that the moderating effect of hometown locations (urban vs. rural) was not significant. This result is consistent with some previous research (Dai and Xu, 2010; Jiao, 2023). Two aspects of the educational context may help explain this result. First, the Chinese government has long implemented strict policies for urban-rural educational equality, and the gap in educational financial investment between urban and rural areas has narrowed significantly (State Council of the People's Republic of China, 2015, 2018). Second, urban and rural areas follow unified curriculum standards, use identical textbooks, and receive similar teacher training (Ministry of Education of the People's Republic of China, 2022). This greater similarity in educational conditions may be relevant to the small difference in creative tendency scores between urban and rural groups.

### 4.5 Insignificant effects of publications

This study found that the moderating effect of publication type was not significant. Two factors may be relevant to this result. First, all studies included in the analysis underwent the same quality assessment, which may have reduced some differences in methodological quality across studies. Second, the quality control system for dissertations has become increasingly robust in recent years. For example, the 2014 “Opinions on Strengthening the Construction of Quality Assurance and Supervision Systems for Degree and Graduate Education” explicitly mandated strengthening dissertation review mechanisms (Academic Degrees Committee of the State Council, and Ministry of Education of the People's Republic of China, 2014). These measures may have improved the overall quality of dissertations and narrowed some differences between dissertations and journal articles, which may be relevant to the non-significant difference across publication types.

### 4.6 Theoretical and educational implications

#### 4.6.1 The theoretical implications

From a theoretical perspective, three points deserve emphasis. First, this study extends previous review research. Previous studies have not systematically and quantitatively synthesized creative tendency among mainland Chinese students, and considerable disagreement remains. By synthesizing the existing evidence through meta-analysis, this study estimated the pooled scores for the Total Score and its four dimensions among mainland Chinese students, thereby providing an overall reference for future research.

Second, this study extends research on the relationship between grade level and creative tendency. Previous studies have generally focused on a single educational stage and have produced inconsistent findings (Cai and Zhu, 2007; Li et al., 2005; Li and Hu, 2016). The present study covered multiple educational stages, including elementary school, middle school, high school, and university. Grade level was associated with differences in the Total Score and the Imaginative dimension, but no significant differences were found for the Risky, Curiosity, or Challenging dimensions. These findings indicate that grade-related differences in creative tendency are dimension-specific and that the Imaginative dimension may be key to understanding such differences.

Third, this study deepens our understanding of the factors associated with creative tendency among Chinese students. Previous research has viewed creativity as culturally situated (Kaufman and Sternberg, 2006) and examined how Confucian values and cultural perceptions are related to Chinese creativity (Niu, 2012; Wong and Niu, 2013). Other studies have focused on the educational context of Chinese students, including exam-oriented education and broader school influences (Ke and Liang, 2023; Niu and Sternberg, 2003). Building on these perspectives, we suggest that future research examine more specific factors, including curriculum content, classroom instruction and management, teachers' instructional behavior, and students' learning processes, including homework. Integrating these factors into a multilevel theoretical framework may help explain differences in creative tendency across student groups and dimensions.

#### 4.6.2 Educational implications

Grade level significantly moderated the total creative tendency score and the imagination dimension, with lower scores observed in the high-school group. These findings suggest that educational activities supporting imagination and creative exploration may deserve particular attention during secondary education.

First, schools should systematically implement creativity cultivation curricula. The lower scores observed in the high-school group suggest that primary and secondary schools could consider incorporating more opportunities for creative exploration into existing curricula (Beghetto, 2013, 2019; Beghetto and Kaufman, 2014; Cotter et al., 2022; Fairweather and Cramond, 2010; Renzulli, 2017). For example, the *Learning and Thinking Activity Curriculum* designed by Hu and colleagues has demonstrated data-supported effectiveness in enhancing creative tendency (Hu and Jia, 2012). At the higher education level, the university group had somewhat higher scores than the high-school group, which may be related to opportunities for creativity-related activities at university. To this end, universities can actively offer interdisciplinary creativity courses (Qian, 2018; Sternberg and Williams, 2001; Sternberg, 2019). For example, Tsinghua University's x-lab and its course “Innovation and Entrepreneurship: Silicon Valley Insights” offer interdisciplinary opportunities for creative exploration (Qian, 2018).

Second, teachers may support students' creative tendency through thoughtfully designed homework. Research indicates that homework creativity is significantly positively correlated with creative tendency (Fan et al., 2022). This suggests that well-designed homework may support students' creative tendency. Specifically, teachers can approach this from two aspects. Teachers can use creative instructional directions when assigning homework. For example, teachers can ask students to offer unique insights or explore multiple solutions (Fan et al., 2022; Soh, 2000). Teachers can also assign different homework to different students. Yu (2025) suggested that innovative homework formats, such as task-based, practice-based, and project-based assignments, may support students' creative tendency. Xie (2021) reported that non-written mathematics homework was associated with less negative outcomes for creative tendency among lower-grade elementary students than repetitive written homework.

Third, students should actively cultivate their own creative tendencies. Creativity is inherently highly autonomous (Karwowski and Beghetto, 2019; Mumford et al., 1991; Pang, 2022). In a creative writing experiment, Amabile (1985) studied 72 participants and found that when the extrinsic motivation group was asked to consider external motivations for writing, their poetry creativity declined significantly. However, when the intrinsic motivation group was guided to focus on the pleasure and challenge of writing itself, creativity was maintained. Therefore, students cab cultivate their own creative tendencies through autonomous learning.

Fourth, instructional assessment should increase the weight of creative tendency. The “be creative” instruction effect demonstrates that explicitly requiring creativity before participants engage in creative activities significantly enhances their creative performance (Niu and Sternberg, 2003). This indicates that clear creativity expectations can effectively guide students' creative tendency. Based on this finding, teachers can assess whether students propose new perspectives, viewpoints, conclusions, or methods as a separate part in homework evaluation (Zhang, 2023), or as a special bonus component, making students aware that excellent homework depends not only on answer correctness but also on thought novelty and uniqueness (Pang, 2009). Consistently including creativity in evaluation may encourage students to value original ideas and become more willing to explore them (Pang, 2022).

## 5 Limitations and issues for further investigation

This study has several limitations. First, no nationally representative norm or empirically established cutoff is available for interpreting WCAP scores among mainland Chinese students. The pooled score therefore cannot determine whether their creative tendency is low, moderate, or high.

Second, the representativeness of the urban–rural analysis was limited. The 29 independent samples included 44,464 participants, accounting for approximately 29.7% of all participants in the review.

Third, the included studies were cross-sectional and spanned more than two decades. The grade-level findings therefore reflect differences between samples and may include cohort effects rather than within-person changes.

Fourth, the moderator analyses examined grade level, gender, urban–rural location, and other factors separately. They could not determine how factors at different levels interact to influence creative tendency.

Correspondingly, four issues warrant further investigation.

First, future research should establish nationally representative WCAP norms and clarify the substantive meaning of different score levels. This may also help explain the numerical difference between the pooled score from mainland China and the reference score from the Taiwan region of China (Lin and Wang, 1994).

Second, future studies should include more samples from regions with different levels of socioeconomic development to examine urban–rural and regional differences more comprehensively.

Third, longitudinal studies should follow the same students across educational stages to distinguish developmental change from cohort and sample-composition effects and to examine why grade-group differences were concentrated in imagination.

Fourth, future multilevel research should investigate how socioeconomic conditions, curriculum and assessment, classroom management, teacher behavior, homework, and opportunities for creative activities jointly shape students' creative tendency.

## 6 Conclusion

This meta-analysis found an overall WCAP creative tendency score of 2.158 among mainland Chinese students. Grade level was a significant moderator: mean scores decreased successively from the elementary-school group to the high-school group, while the mean score in the university group was close to that in the middle-school group. A significant grade-level difference was also observed for the Imaginative dimension. Gender, hometown location, and publication type showed no significant moderating effects. Because the evidence was largely cross-sectional, this pattern describes differences between grade groups rather than changes within the same students. The limited evidence for hometown-location comparisons and the possibility of interactions among factors also warrant further study.

## Data Availability

The datasets presented in this study can be found in online repositories. The names of the repository/repositories and accession number(s) can be found in the article/Supplementary material.

## Supplementary material

Supplementary Table S1 Study features extracted from the primary studies.
