# Supplementary material for: Creative tendency of Chinese students: a meta-analysis, 1999–2025
Source: Front Psychol. 2026 Oct 20;17:1957163. doi: 10.3389/fpsyg.2026.1957163 (PMC13727631; doi:10.3389/fpsyg.2026.1957163)
Supplement: Supplementary file 1 [file Supplementary_file_1.DOCX]

* Those listed here are the 211 primary studies included in the current meta analysis.

An, H. (2023). Research on information technology teaching for cultivating junior high school students' innovative ability under the concept of maker education (Master's thesis). Minnan Normal University. [https://doi.org/10.27726/d.cnki.gzzsf.2023.000494](https://doi.org/10.27726/d.cnki.gzzsf.2023.000494)

An, M. (2011). Cultivation activities and experiments of junior high school students' creativity (Master’s thesis). Nanjing Normal University. [https://kns.cnki.net/kcms2/article/abstract?v=YtMUyApXHwaarCAWY2noVjEvEGbFvqZQX_J3LCd_xp0X82qkdTJv81tecLQf0HQ36h0IrG7X1mm6nnXjjAexpVApaxOExwDsyVNYj_7K1-sU-l6qLYh05ZYcU2WmP2YgiHHCRDFKsq9xbfmv6q0LTUFZ8FvWVMB1iErDXPi8M_g30AF6BX8O6A==&uniplatform=NZKPT&language=CHS](https://kns.cnki.net/kcms2/article/abstract?v=YtMUyApXHwaarCAWY2noVjEvEGbFvqZQX_J3LCd_xp0X82qkdTJv81tecLQf0HQ36h0IrG7X1mm6nnXjjAexpVApaxOExwDsyVNYj_7K1-sU-l6qLYh05ZYcU2WmP2YgiHHCRDFKsq9xbfmv6q0LTUFZ8FvWVMB1iErDXPi8M_g30AF6BX8O6A==&uniplatform=NZKPT&language=CHS)

Cai, C. (2018). A correlational study on English proficiency and creativity in Chinese-English sequential bilinguals: Based on the threshold theory. Journal of Guangdong University of Foreign Studies, 29(2), 96–102.

Cai, J. Y., Xiao, T. R., & Bai, Y. F. (2001). An experimental study on cultivating students' creativity through subject teaching. Journal of Henan Institute of Education (Philosophy and Social Sciences Edition), (01), 117-120.

Cai, X. Y. (2017). Research on middle school students' creativity cultivation strategies based on drama teaching (Master's thesis). Central China Normal University.

Cai, X. Y., & Zhu, Y. J. (2007). A study on the correlation among creative tendency, intelligence, and academic achievement of primary and middle school students. Psychological Development and Education, (02), 36-41. [https://doi.org/10.16187/j.cnki.issn1001-4918.2007.02.007](https://doi.org/10.16187/j.cnki.issn1001-4918.2007.02.007)

Cao, Y. J. (2021). The influence of university teachers' classroom teaching strategies on college students' creativity (Master's thesis). Shihezi University. [https://doi.org/10.27332/d.cnki.gshzu.2021.000189](https://doi.org/10.27332/d.cnki.gshzu.2021.000189)

Chai, S. X., & Han, S. F. (2008). Investigation of creative tendency among nursing undergraduates. Chinese Nursing Research, (02), 114-116.

Chen, D. J., Liang, G. D., & Wang, Y. H. (2020). The impact of creative personality on college students' innovative self-efficacy: The mediating role of achievement motivation. China Journal of Health Psychology, 28(8), 1272-1277. [https://doi.org/10.13342/j.cnki.cjhp.2020.08.034](https://doi.org/10.13342/j.cnki.cjhp.2020.08.034)

Chen, D. T. (2018). A study on the relationship between college students’ self-efficacy and creative tendency. *Journal of Science & Education (Electronic Edition)*, 1(1), 10–12.

Chen, D., & Zhang, J. M. (2021). An empirical survey on the development of creativity among normal students in higher vocational colleges: Taking Chuannan Preschool Education College as an example. Educational Observation, 10(38), 30-32. [https://doi.org/10.16070/j.cnki.cn45-1388/g4s.2021.38.010](https://doi.org/10.16070/j.cnki.cn45-1388/g4s.2021.38.010)

Chen, J. (2019). *The impact of "reading and drawing diagrams" teaching in primary school labor and technical courses on the creativity of senior-grade students* (Master’s thesis, Shanghai Normal University). Retrieved from https://kns.cnki.net/kcms2/article/abstract?v=VYuoLtjwl8OtyWbazSkX1jZEgXP7j2fpGD_mJUeWrTmrUilaeVJJc4H7racq1lSIwUtOuZ7aV5rRrLYy1SQQLgPMQkR-6DhhDbdBXg1VKYQt13wYo5EoiTewKUKxiUikqfVAibUYI4XNwf3TUfMl-mA06lkuLE6zh2AzYN_bSe_EjOjVU0S0Mw==

Chen, J. X., & Zhu, X. F. (2010). A study on the relationship among college students' achievement motivation, personality characteristics, and creativity tendency. Journal of Wuhan Polytechnic, 9(6), 96-99.

Chen, M., & Wang, Q. (2021). A study on the relationship between creativity tendency and learning burnout among students in higher vocational normal colleges. Journal of Hebei Energy Institute of Vocation and Technology, 21(2), 52-54.

Chen, N. (2020). Research on factors influencing innovation ability of doctoral students in universities (Master's thesis). Nanjing Agricultural University. [https://doi.org/10.27244/d.cnki.gnjnu.2020.000515](https://doi.org/10.27244/d.cnki.gnjnu.2020.000515)

Chen, X. R. (2013). An experimental study on the “Learning to Think” activity curriculum for reducing academic burden and cultivating innovative quality in primary school students (Master’s thesis). Shaanxi Normal University. [https://kns.cnki.net/kcms2/article/abstract?v=YtMUyApXHwY3jO_TJ-mU5o9-IV51aP3xIFv7AzAzj5r-w8sZs_bMneaZW-2RX_wZzM0XD90CBGL1FXmmM-wEW8hacvS2QZdSN1JNj4Sq994wR9AlZvBoaKg0YQFIG4qfi4bqkCd93vmyhHeekvoefr35SUmleT0sxr_Vk2b02XpHrsFoednmHw==&uniplatform=NZKPT&language=CHS](https://kns.cnki.net/kcms2/article/abstract?v=YtMUyApXHwY3jO_TJ-mU5o9-IV51aP3xIFv7AzAzj5r-w8sZs_bMneaZW-2RX_wZzM0XD90CBGL1FXmmM-wEW8hacvS2QZdSN1JNj4Sq994wR9AlZvBoaKg0YQFIG4qfi4bqkCd93vmyhHeekvoefr35SUmleT0sxr_Vk2b02XpHrsFoednmHw==&uniplatform=NZKPT&language=CHS)

Chen, Y. H. (2020). A study on the relationship among school environment, creative self-efficacy, and creativity of design students (Master's thesis). Zhejiang A&F University. [https://doi.org/10.27756/d.cnki.gzjlx.2020.000271](https://doi.org/10.27756/d.cnki.gzjlx.2020.000271)

Chen, Y. M. (2014). Research on the impact of applying cooperative learning in information technology teaching on students’ creativity. Journal of Heilongjiang College of Education, 33(12), 44-46.

Chen, Y. Y. (2022). The relationship between locus of control and creativity (Master's thesis). Southwest University. [https://doi.org/10.27684/d.cnki.gxndx.2022.004238](https://doi.org/10.27684/d.cnki.gxndx.2022.004238)

Chen, Y., Wei, X., Yu, M. J., & Wang, H. R. (2011). A correlational study on the cultivation of creative personality in primary school students and family environment. Journal of Traffic Medicine, 25(4), 348-351.

Chen, Z. F. (2020). The relationship between creative tendency and self-regulated learning in upper-grade primary school students (Master's thesis). Guangzhou University. [https://doi.org/10.27040/d.cnki.ggzdu.2020.000708](https://doi.org/10.27040/d.cnki.ggzdu.2020.000708)

Cheng, D. L. (2023). The relationship between psychological resilience and creative tendency in middle school students (Master's thesis). Bohai University. [https://doi.org/10.27190/d.cnki.gjzsc.2023.000001](https://doi.org/10.27190/d.cnki.gjzsc.2023.000001)

Chu, X. (2024). Research on cultivating creative thinking ability in high school biology based on PBL teaching model (Master's thesis). Yangzhou University.https://doi.org/10.27441/d.cnki.gyzdu.2024.002642

Dai, B., & Xu, X. L. (2010). General self-efficacy and its relationship with creative personality in college students. China Journal of Health Psychology, 18(7), 881-883. [https://doi.org/10.13342/j.cnki.cjhp.2010.07.053](https://doi.org/10.13342/j.cnki.cjhp.2010.07.053)

Dai, Y., Chen, S., Mao, F., & Xu, J. (2024). Determinants of creativity in migrant versus urban children: A case study in China. *Children*, 11(7), 802.

Deng, J. (2013). Research on the cultivation of primary school students' innovative ability through robot classroom teaching (Master's thesis). Sichuan Normal University. [https://kns.cnki.net/kcms2/article/abstract?v=YtMUyApXHwb0gROYXAc2-k_eQ3lEjYnMpaPT5FjKfz-JyVLHKiSOUfEn20Ppm2fq_gO-jBsC1tGoIx8DRF5H9oMuN5agcX1EefAnc6ML-WTTuPrSrjX325cYQ9vIMKi1sTO0fcnv56ehl3-6KxHhBMID_uNF9KQ3dtMAHHESyFkVccRYQGcCbw==&uniplatform=NZKPT&language=CHS](https://kns.cnki.net/kcms2/article/abstract?v=YtMUyApXHwb0gROYXAc2-k_eQ3lEjYnMpaPT5FjKfz-JyVLHKiSOUfEn20Ppm2fq_gO-jBsC1tGoIx8DRF5H9oMuN5agcX1EefAnc6ML-WTTuPrSrjX325cYQ9vIMKi1sTO0fcnv56ehl3-6KxHhBMID_uNF9KQ3dtMAHHESyFkVccRYQGcCbw==&uniplatform=NZKPT&language=CHS)

Du, Y. F. (2009). A study on the development of creativity in primary school students. Shanghai Research on Education, (3), 36–38. https://doi.org/10.16194/j.cnki.31-1059/g4.2009.03.016

Du, Y. F., & Niu, F. P. (2013). A study on the influence of creative personality on primary school students' academic achievement. Education Exploration, (02), 133-135.

Du, Y. J. (2022). Design and practical research of robot unit theme teaching in junior high school oriented to creativity cultivation (Master's thesis). Central China Normal University. [https://doi.org/10.27159/d.cnki.ghzsu.2022.002603](https://doi.org/10.27159/d.cnki.ghzsu.2022.002603)

Fang, H. (2013). A study on the level of creativity tendency and its relationship with psychological traits among nursing undergraduates (Master's thesis). Shandong University. [https://kns.cnki.net/kcms2/article/abstract?v=YtMUyApXHwaaDN7Nlq7ToQ62PNqyvH6epH4WNAWpS0awX4UF82dPJT275exmcyBNFaxJ1D4ixp4YLEYlIRkIFPW_Gq-30LjX3SQ0N-H9qBfRXCKyIgNEoOXcI1pBJO2ZrgDKNxNKtpqgvlZP5NyZw4fYE55coMqLru4_qdCQXxnL-_Z7UHnDoA==&uniplatform=NZKPT&language=CHS](https://kns.cnki.net/kcms2/article/abstract?v=YtMUyApXHwaaDN7Nlq7ToQ62PNqyvH6epH4WNAWpS0awX4UF82dPJT275exmcyBNFaxJ1D4ixp4YLEYlIRkIFPW_Gq-30LjX3SQ0N-H9qBfRXCKyIgNEoOXcI1pBJO2ZrgDKNxNKtpqgvlZP5NyZw4fYE55coMqLru4_qdCQXxnL-_Z7UHnDoA==&uniplatform=NZKPT&language=CHS)

Feng, X. E. (2021). Design and practice of hands-on production assignments in junior high school physics (Master's thesis). Nanjing Normal University. [https://doi.org/10.27245/d.cnki.gnjsu.2021.001043](https://doi.org/10.27245/d.cnki.gnjsu.2021.001043)

Fu, B. Z. (2013). An experimental study on the impact of goal orientation intervention in vocational college basketball elective courses on creative personality (Master's thesis). Hebei Normal University. [https://kns.cnki.net/kcms2/article/abstract?v=YtMUyApXHwa_iWq-SgMfD8TF9NEC5kYbCyv95pGBML30g7fncMphIQZa9u4iK-jp6t0BiMprPatJpiOqyAbdysgiDBY_tFUiM3er7gj0FNoWmcwIcmQofo3QIl6mHgPTsIg0yn_OR1VsMKYlvnJNJnprlby2cXpv58R0bcMZ749t_eGldtAT4g==&uniplatform=NZKPT&language=CHS](https://kns.cnki.net/kcms2/article/abstract?v=YtMUyApXHwa_iWq-SgMfD8TF9NEC5kYbCyv95pGBML30g7fncMphIQZa9u4iK-jp6t0BiMprPatJpiOqyAbdysgiDBY_tFUiM3er7gj0FNoWmcwIcmQofo3QIl6mHgPTsIg0yn_OR1VsMKYlvnJNJnprlby2cXpv58R0bcMZ749t_eGldtAT4g==&uniplatform=NZKPT&language=CHS)

Fu, R. E. (2023). Practical research on cultivating students' creative thinking through the ATDE teaching model in high school biology (Master's thesis). Hainan Normal University. [https://doi.org/10.27719/d.cnki.ghnsf.2023.000091](https://doi.org/10.27719/d.cnki.ghnsf.2023.000091)

Gai, G. Y. (2021). Research on the cultivation of creativity in junior high school students from the perspective of high-efficacy positive personality education (Master's thesis). Harbin Normal University. [https://doi.org/10.27064/d.cnki.ghasu.2021.000348](https://doi.org/10.27064/d.cnki.ghasu.2021.000348)

Gao, B. Y. (2023). The impact of anxiety on adolescents' creativity (Master's thesis). Sichuan Normal University. [https://doi.org/10.27347/d.cnki.gssdu.2023.000080](https://doi.org/10.27347/d.cnki.gssdu.2023.000080)

**Gao, S., & Zeng, H. (2012).** Investigation and analysis of the current situation of college students' creativity tendency. China Electric Power Education, (10), 138–139+153.

Gou, R. (2021). Learning intervention research on graphical programming oriented to creative thinking cultivation (Master's thesis). Northeast Normal University. [https://doi.org/10.27011/d.cnki.gdbsu.2021.001370](https://doi.org/10.27011/d.cnki.gdbsu.2021.001370)

Group Research. (1999). An experimental study on cultivating students' creativity in natural science teaching. Journal of Luoyang Teachers College, (02), 129-135+139. [https://doi.org/10.16594/j.cnki.41-1302/g4.1999.02.048](https://doi.org/10.16594/j.cnki.41-1302/g4.1999.02.048)

Guo, D. M., Wu, Y. H., & Ding, H. H. (2018). A survey on the development status of creativity tendency among students in higher vocational colleges. Education and Vocation, (24), 107-112. [https://doi.org/10.13615/j.cnki.1004-3985.2018.24.022](https://doi.org/10.13615/j.cnki.1004-3985.2018.24.022)

Guo, E. R. (2017). An empirical analysis of creative tendency among students in private universities. Higher Education of Sciences, (02), 59-63+99.

Guo, Y. J. (2018). The development and relationship between creative tendency and achievement motivation of preschool education majors in higher vocational colleges (Master’s thesis). Zhejiang University. [https://kns.cnki.net/kcms2/article/abstract?v=YtMUyApXHwZNKA3fANF-maGrBX02E4H6mLZr87VFa2HHa1TVRdvYaaXF12hFfh0EqcQcYBQ-whPiM5kossqHmLYHAvm5pCLvMCioZEPGjaIDOzACB7wY1fxTmrv6wKl06YOpk2aMOSBoCmApSjp5GmDkEBJayqdS3AiS9GxNhUnyC4bbA-4hJw==&uniplatform=NZKPT&language=CHS](https://kns.cnki.net/kcms2/article/abstract?v=YtMUyApXHwZNKA3fANF-maGrBX02E4H6mLZr87VFa2HHa1TVRdvYaaXF12hFfh0EqcQcYBQ-whPiM5kossqHmLYHAvm5pCLvMCioZEPGjaIDOzACB7wY1fxTmrv6wKl06YOpk2aMOSBoCmApSjp5GmDkEBJayqdS3AiS9GxNhUnyC4bbA-4hJw==&uniplatform=NZKPT&language=CHS)

Han, H. Y. (2012). A study on the relationship among personality traits, achievement motivation, and creative tendency in high school students (Master's thesis). Shanxi Normal University. [https://kns.cnki.net/kcms2/article/abstract?v=YtMUyApXHwaJrW5QrWcalGEv0LQIQRGdqLFoBiCgTKJ5ya4zFTtHNs9LltL_3agr0G7geibCF775FKrvmV4DYVtvqaR8qyEk5qpW3WBgR1dK1E9JFqP3HYIUhN9G7qtZbwD8vl6F2KHAh7GK_K6OiHW7xlsAn_JgZcD6ix43HhUcFK8A9ta7mA==&uniplatform=NZKPT&language=CHS](https://kns.cnki.net/kcms2/article/abstract?v=YtMUyApXHwaJrW5QrWcalGEv0LQIQRGdqLFoBiCgTKJ5ya4zFTtHNs9LltL_3agr0G7geibCF775FKrvmV4DYVtvqaR8qyEk5qpW3WBgR1dK1E9JFqP3HYIUhN9G7qtZbwD8vl6F2KHAh7GK_K6OiHW7xlsAn_JgZcD6ix43HhUcFK8A9ta7mA==&uniplatform=NZKPT&language=CHS)

He, Y. N. (2019). Creativity, innovation, and economic growth (Master's thesis). Wuhan University. [https://doi.org/10.27379/d.cnki.gwhdu.2019.001063](https://doi.org/10.27379/d.cnki.gwhdu.2019.001063)

Henan Province Puyang City Second Experimental Primary School Research Group. (2000). An experimental study on cultivating students' creativity in subject teaching. Journal of Tianjin Academy of Educational Science, (03), 30-35. [https://doi.org/10.16137/j.cnki.cn12-1303/g4.2000.03.010](https://doi.org/10.16137/j.cnki.cn12-1303/g4.2000.03.010)

Hu, W. P., Zhao, X. M., Jia, P. Y., & Chen, Y. H. (2017). The effect of the "Learning to Think" online activity on primary school students' creativity: The moderating role of cognitive style. Psychological Development and Education, 33(3), 257–264. [https://doi.org/10.16187/j.cnki.issn1001-4918.2017.03.01](https://doi.org/10.16187/j.cnki.issn1001-4918.2017.03.01)

Huang, G. N. (2024). Design of Scratch curriculum learning activities in primary school aimed at cultivating innovative ability (Master's thesis). Hainan Normal University. [https://doi.org/10.27719/d.cnki.ghnsf.2024.000641](https://doi.org/10.27719/d.cnki.ghnsf.2024.000641)

Huang, H. L. (2018). Research on the relationship among achievement motivation, mental health, and creativity tendency of nursing master's students (Master's thesis). Yanbian University. [https://kns.cnki.net/kcms2/article/abstract?v=YtMUyApXHwYjH5go8dLbLlH_uI9RWgpTN59LYF0dFCNnVhoAfPm0qOxAzxapSr1T6zQjY06FbqwINzbzxyvZ2G-1c07LXPusfal3WRTnAJgsfGOCu6GBOL1mzfAUYD8hndr2JYEvXhtenp7ZI3P2RU3VduIKUVJ4GoYlBnf09Hk3f5n_h3tXxA==&uniplatform=NZKPT&language=CHS](https://kns.cnki.net/kcms2/article/abstract?v=YtMUyApXHwYjH5go8dLbLlH_uI9RWgpTN59LYF0dFCNnVhoAfPm0qOxAzxapSr1T6zQjY06FbqwINzbzxyvZ2G-1c07LXPusfal3WRTnAJgsfGOCu6GBOL1mzfAUYD8hndr2JYEvXhtenp7ZI3P2RU3VduIKUVJ4GoYlBnf09Hk3f5n_h3tXxA==&uniplatform=NZKPT&language=CHS)

Huang, J. N. (2023). Research on project-based teaching strategies of primary school information technology curriculum oriented to creative thinking cultivation (Master's thesis). Guangxi Normal University. [https://doi.org/10.27036/d.cnki.ggxsu.2023.000635](https://doi.org/10.27036/d.cnki.ggxsu.2023.000635)

Huang, W. Y. (2010). Applied research on inquiry teaching in junior high school information technology for cultivating students' innovative ability (Master's thesis). Shandong Normal University. [https://kns.cnki.net/kcms2/article/abstract?v=YtMUyApXHwaoRAsUhPgbdzenqwgY9mYPJ0exxCXFiKrJ2cPCnTjmRvxqlzUXWCnuLiji4mcDIs59xJ95OxqUtK70BA8sCa1rNa9CDCthxxId6M4k5d08JTm7KZpkvZH-EdOJHmvJVDj-Yuzr62bYHaRK3uhR4MbQBK8Mj9DPRlWj-Pr-KcFX6A==&uniplatform=NZKPT&language=CHS](https://kns.cnki.net/kcms2/article/abstract?v=YtMUyApXHwaoRAsUhPgbdzenqwgY9mYPJ0exxCXFiKrJ2cPCnTjmRvxqlzUXWCnuLiji4mcDIs59xJ95OxqUtK70BA8sCa1rNa9CDCthxxId6M4k5d08JTm7KZpkvZH-EdOJHmvJVDj-Yuzr62bYHaRK3uhR4MbQBK8Mj9DPRlWj-Pr-KcFX6A==&uniplatform=NZKPT&language=CHS)

Huang, X. (2022). Research on the application of BOPPPS teaching model in primary school graphical programming teaching from the perspective of creative thinking cultivation (Master's thesis). Henan University. [https://doi.org/10.27114/d.cnki.ghnau.2022.001747](https://doi.org/10.27114/d.cnki.ghnau.2022.001747)

Ji, G. J. (2019). Research on knowledge self-construction for the development of creativity in junior high school students (Doctoral dissertation). Shanghai Normal University. [https://doi.org/10.27312/d.cnki.gshsu.2019.000182](https://doi.org/10.27312/d.cnki.gshsu.2019.000182)

Ji, H. X., Yu, S. X., & Shen, Y. (2019). A survey on the current status of creativity tendency among college students in an agricultural university. Youth and Society.

Jia, C. L. (2009). A correlational study on learning styles and creativity tendency of nursing master's students in Beijing and Shanxi (Master's thesis). Shanxi Medical University. [https://kns.cnki.net/kcms2/article/abstract?v=YtMUyApXHwa3cKMbbcIkp7iRNT8tldD8IFpxY544fAb8GvJPltV6XHtDEZwiqMWhBAyTsJF_-bGG81J07Ug5RR2ZECgBEXdm8K80pKjH0u1TvR5Rxc97_83nfcCC_jUmJaklXXJOwkUa5m4KKiUMQO-w0FKq7tzNVs0YnsMKE3JZTd3hw1ufwg==&uniplatform=NZKPT&language=CHS](https://kns.cnki.net/kcms2/article/abstract?v=YtMUyApXHwa3cKMbbcIkp7iRNT8tldD8IFpxY544fAb8GvJPltV6XHtDEZwiqMWhBAyTsJF_-bGG81J07Ug5RR2ZECgBEXdm8K80pKjH0u1TvR5Rxc97_83nfcCC_jUmJaklXXJOwkUa5m4KKiUMQO-w0FKq7tzNVs0YnsMKE3JZTd3hw1ufwg==&uniplatform=NZKPT&language=CHS)

Jia, X. J. (2017). The cognitive neural mechanisms and plasticity of children's creativity (Doctoral dissertation). Shaanxi Normal University. [https://kns.cnki.net/kcms2/article/abstract?v=YtMUyApXHwZI2B_jWa1_P0RCloe1e4UaHKCjzAvNeC_lgjbEuDCqi9qEd0SlaJB9hDDxwuFidPT19f7760RmsTirSS5-1RRq2htpbWWtX36t95zCEzW_Grvm3gVR3UcgP8Vnupa0qWW3wrD-Owvf2fYguR5ujzhle03wh9HjbpJ1oqU16Ih6ueeKx8Bw3Eiz&uniplatform=NZKPT&language=CHS](https://kns.cnki.net/kcms2/article/abstract?v=YtMUyApXHwZI2B_jWa1_P0RCloe1e4UaHKCjzAvNeC_lgjbEuDCqi9qEd0SlaJB9hDDxwuFidPT19f7760RmsTirSS5-1RRq2htpbWWtX36t95zCEzW_Grvm3gVR3UcgP8Vnupa0qWW3wrD-Owvf2fYguR5ujzhle03wh9HjbpJ1oqU16Ih6ueeKx8Bw3Eiz&uniplatform=NZKPT&language=CHS)

Jiang, Y. (2016). A study on the impact of creative thinking teaching intervention in physical education classes on the creativity of secondary vocational school students. Journal of Taiyuan Urban Vocational College, (01), 105-107. [https://doi.org/10.16227/j.cnki.tycs.2016.0048](https://doi.org/10.16227/j.cnki.tycs.2016.0048)

Jiang, Y. (2021). Research on the application effect of instructional design for "Primary School Robotics" oriented to creativity cultivation (Master's thesis). Yunnan Normal University. [https://doi.org/10.27459/d.cnki.gynfc.2021.001119](https://doi.org/10.27459/d.cnki.gynfc.2021.001119)

Jiao, L. Y. (2023). A study on the survey of creativity tendency and self-efficacy among female students in higher vocational colleges. Journal of Jiangxi Vocational and Technical College of Electricity, 36(1), 163–165.

Kaifeng Yishi Fuxiao Research Group. (1999). An experimental study on cultivating students' creativity in mathematics teaching. Journal of Ningbo University (Educational Science Edition), (04), 32-36.

Kong, X. W. (2016). A study on creativity of students in the science experimental class at China University of Petroleum (East China) (Master's thesis). China University of Petroleum (East China). [https://kns.cnki.net/kcms2/article/abstract?v=YtMUyApXHwZ8ZKblBbIgpIkZwdwDtHrwfizOliDHa7vcpECVpEloj4Lh1CV0zm0FeXpnUl6UTgPazPAR_0PXP_uhCJrjnBtbV_sUCoJ7UORFUawE8TftJKGZ42u-RI7foDeTYJoUxzi-opAnGuTtgWoXY3ZEGGTmM0PKVbwYK-p2jhEeOPt0hQ==&uniplatform=NZKPT&language=CHS](https://kns.cnki.net/kcms2/article/abstract?v=YtMUyApXHwZ8ZKblBbIgpIkZwdwDtHrwfizOliDHa7vcpECVpEloj4Lh1CV0zm0FeXpnUl6UTgPazPAR_0PXP_uhCJrjnBtbV_sUCoJ7UORFUawE8TftJKGZ42u-RI7foDeTYJoUxzi-opAnGuTtgWoXY3ZEGGTmM0PKVbwYK-p2jhEeOPt0hQ==&uniplatform=NZKPT&language=CHS)

Lai, J. F. (2019). The impact of running aerobic exercise on creative thinking and its mechanism exploration (Master's thesis). Southwest University. [https://kns.cnki.net/kcms2/article/abstract?v=YtMUyApXHwYshTf5FAPlE1b4a_ykTNg-qLDEz6O4ihT7KQSQfd2ktR0oWkAw9h_3Yt8aU9ibOdUCsZx9vQiTPE4XdUYMW3ogEMWSZBO36fzGr3qVQi5uobsciaqHkfBhBTgp14--hq8ZbsmblgMnZjaHtEdqTF4VrIMahzZiBgu0N1pH1T8IvA==&uniplatform=NZKPT&language=CHS](https://kns.cnki.net/kcms2/article/abstract?v=YtMUyApXHwYshTf5FAPlE1b4a_ykTNg-qLDEz6O4ihT7KQSQfd2ktR0oWkAw9h_3Yt8aU9ibOdUCsZx9vQiTPE4XdUYMW3ogEMWSZBO36fzGr3qVQi5uobsciaqHkfBhBTgp14--hq8ZbsmblgMnZjaHtEdqTF4VrIMahzZiBgu0N1pH1T8IvA==&uniplatform=NZKPT&language=CHS)

Lan, J. J., Li, M., Wei, X., Du, J., & Li, Y. (2019). The relationship between parenting styles and pupils' creativity: The mediating role of learning adaptability. Journal of Guizhou Education University, *35*(5), 79–84. <https://doi.org/10.13391/j.cnki.issn.1674-7798.2019.05.013>

Li, D. Y. (2021). Research on the application of the CPS model supported by mind maps in junior high school information technology teaching (Master's thesis). Tianjin Normal University. [https://doi.org/10.27363/d.cnki.gtsfu.2021.000306](https://doi.org/10.27363/d.cnki.gtsfu.2021.000306)

Li, F. G. (2009). A correlational study on creativity, fatty acids, and parenting styles in children with ADHD (Master's thesis). Dalian Medical University. [https://kns.cnki.net/kcms2/article/abstract?v=YtMUyApXHwbDeBDMyx1m7fq4xTxBMp70_e4MoGcYV0K9i2MCytKSfgV4i2DuK2qYxOeOwrqby9Jd_jteuux98bJZfIiobbs_WxU9l9v7jZFiAd4YfTV0vmq7pgwQxKqQ68yRx5ZL05LHA2H-pLevxNqnmGEtUr-JLUy8KDbA5V6lk-2rZn3pNw==&uniplatform=NZKPT&language=CHS](https://kns.cnki.net/kcms2/article/abstract?v=YtMUyApXHwbDeBDMyx1m7fq4xTxBMp70_e4MoGcYV0K9i2MCytKSfgV4i2DuK2qYxOeOwrqby9Jd_jteuux98bJZfIiobbs_WxU9l9v7jZFiAd4YfTV0vmq7pgwQxKqQ68yRx5ZL05LHA2H-pLevxNqnmGEtUr-JLUy8KDbA5V6lk-2rZn3pNw==&uniplatform=NZKPT&language=CHS)

Li, Q. (2015). Developmental characteristics, influencing factors, and intervention study of creative thinking in senior high school students (Master’s thesis). University of Jinan. https://doi.org/10.7666/d.D01121690

Li, Q. (2015). The developmental characteristics, influencing factors and intervention of creative thinking in high school students (Master's thesis). University of Jinan. [https://kns.cnki.net/kcms2/article/abstract?v=YtMUyApXHwY3JZ8DLZ8k56Mkfqu2Yod9B3lRaGiL8WeJBXEL2gsi90DSka14p5xVCO0y1mkw7dlHR9_UzPb4cXXOggycoI3CYELLqWZlxySPNDNnR5oXBxUSynYh0X4aS35kn5CmNrdHZFVivI3FMq9pQvW-q4qIeyuRFRutAzLtXFquWJW1IA==&uniplatform=NZKPT&language=CHS](https://kns.cnki.net/kcms2/article/abstract?v=YtMUyApXHwY3JZ8DLZ8k56Mkfqu2Yod9B3lRaGiL8WeJBXEL2gsi90DSka14p5xVCO0y1mkw7dlHR9_UzPb4cXXOggycoI3CYELLqWZlxySPNDNnR5oXBxUSynYh0X4aS35kn5CmNrdHZFVivI3FMq9pQvW-q4qIeyuRFRutAzLtXFquWJW1IA==&uniplatform=NZKPT&language=CHS)

Li, Q. F., & Hu, W. S. (2016). Research on the development of college students' creativity tendency: Based on a survey of J University of Technology in Beijing. Journal of Beijing University of Technology (Social Sciences Edition), 16(2), 71–80.

Li, W. (2009). A study on the influence of computer games on the creative tendencies of primary and secondary school students. *Modern Educational Technology*, *19(7),* 73–77.

Li, X. P., Zhang, Q. L., & He, H. B. (2005). A preliminary test on the development of creative tendency in middle school students. Journal of Southwest China Normal University (Humanities and Social Sciences Edition), (06), 65-68. [https://doi.org/10.13718/j.cnki.xdsk.2005.06.014](https://doi.org/10.13718/j.cnki.xdsk.2005.06.014)

Li, Y. F. (2021). A correlational study on creative personality and mathematics problem-posing ability of tenth-grade students (Master's thesis). Yunnan Normal University. [https://doi.org/10.27459/d.cnki.gynfc.2021.000947](https://doi.org/10.27459/d.cnki.gynfc.2021.000947)

Li, Y. L., Cao, F. L., Dong, F. H., Kong, Z., & Zhu, Y. H. (2012). A study on the relationship between creativity tendency and psychological traits among nursing undergraduates. *Journal of Nursing Science: Surgery Edition*， 27(5), 77–79.

Liang, C. (2024). A study on the teaching practice of the ATDE instructional model for cultivating students' creative thinking (Master's thesis). Yangzhou University. [https://doi.org/10.27441/d.cnki.gyzdu.2024.001175](https://doi.org/10.27441/d.cnki.gyzdu.2024.001175)

Liang, C. X., & Shen, H. (2021). Research on the impact of creativity on entrepreneurial intention: A comparison between urban and rural college students. Science and Technology Management Research, 41(9), 77–83.

Liang, F. (2014). A study on the relationship between emotional intelligence and creativity (Master's thesis). Southwest University. [https://kns.cnki.net/kcms2/article/abstract?v=YtMUyApXHwa4JDer4ffDNqj5O6spxX1DcysGjonPhp2O-zX76l6P2u4lDnFPKTuSYQKHFqlM7hZ40N0oQvpyu7pGE70-8uJDOAasUGvcKpS_WGJW2DF749UJ2QNER-4goLmnSLJ4uvZP-_9IIEsA_funum89G_Spca4dmOcp9dCHYtk7fJR8_g==&uniplatform=NZKPT&language=CHS](https://kns.cnki.net/kcms2/article/abstract?v=YtMUyApXHwa4JDer4ffDNqj5O6spxX1DcysGjonPhp2O-zX76l6P2u4lDnFPKTuSYQKHFqlM7hZ40N0oQvpyu7pGE70-8uJDOAasUGvcKpS_WGJW2DF749UJ2QNER-4goLmnSLJ4uvZP-_9IIEsA_funum89G_Spca4dmOcp9dCHYtk7fJR8_g==&uniplatform=NZKPT&language=CHS)

Liang, Q. Y. (2021). Design and implementation of Scratch teaching model oriented to creative thinking cultivation (Master's thesis). Ningbo University. [https://doi.org/10.27256/d.cnki.gnbou.2021.000056](https://doi.org/10.27256/d.cnki.gnbou.2021.000056)

Liang, Y. J. (2019). Research on STEAM teaching mode for cultivating innovative ability in primary and secondary schools (Master's thesis). Guangzhou University. [https://kns.cnki.net/kcms2/article/abstract?v=YtMUyApXHwYQDnvoG2WPIKqyMR3so_tuCpyjG-l3W510_GGoO24UtcTEdeT_VIHIvDK1yJo0xgDW7js9EJmOuSDPZ0a9zU08EEYg_ZCliHdrlOJ1YoBKEhvkgyVC2TKtFaSuPWJOdvN1bQeMSYCnlJ4VAqeC9v07o9409kj_41y32rvZOxJNVw==&uniplatform=NZKPT&language=CHS](https://kns.cnki.net/kcms2/article/abstract?v=YtMUyApXHwYQDnvoG2WPIKqyMR3so_tuCpyjG-l3W510_GGoO24UtcTEdeT_VIHIvDK1yJo0xgDW7js9EJmOuSDPZ0a9zU08EEYg_ZCliHdrlOJ1YoBKEhvkgyVC2TKtFaSuPWJOdvN1bQeMSYCnlJ4VAqeC9v07o9409kj_41y32rvZOxJNVw==&uniplatform=NZKPT&language=CHS)

Lin, B. (2006). Inquiry teaching in junior high school biology and the cultivation of students' creative personality (Master's thesis). Fujian Normal University. [https://kns.cnki.net/kcms2/article/abstract?v=YtMUyApXHwY2WcKSIroMhZNQU20FezhGtrCDY1r6AsQhed_-fXQQJp_BN2WdklrAFURkEAkYPvbnt5m2ea8ZX5_gVNXpMTAm4X44NEPY5gy-BlEOYbtgyfk0Evku8tQ-cjXifcqaE6iWX6ZZpuv3hKFW9V8Uiikfsvdb8EGMlE-7EM5WcTn-GQ==&uniplatform=NZKPT&language=CHS](https://kns.cnki.net/kcms2/article/abstract?v=YtMUyApXHwY2WcKSIroMhZNQU20FezhGtrCDY1r6AsQhed_-fXQQJp_BN2WdklrAFURkEAkYPvbnt5m2ea8ZX5_gVNXpMTAm4X44NEPY5gy-BlEOYbtgyfk0Evku8tQ-cjXifcqaE6iWX6ZZpuv3hKFW9V8Uiikfsvdb8EGMlE-7EM5WcTn-GQ==&uniplatform=NZKPT&language=CHS)

Liu, C. Y., Zhang, H. M., Zhang, L., Shi, J., Shi, G. Y., & Tao, R. (2013). The influence of parenting styles on creativity tendency and self-efficacy in middle school students. China Journal of Health Psychology, 21(4), 589-591. [https://doi.org/10.13342/j.cnki.cjhp.2013.04.013](https://doi.org/10.13342/j.cnki.cjhp.2013.04.013)

Liu, C. Y., Zhang, H. M., Zhang, Y., Li, H., Xue, Z. X., Gao, J. C., & Tao, R. (2014). A comparative study on creativity tendency and self-efficacy of junior high school students with internet addiction. China Journal of Health Psychology, 22(2), 232–234. [https://doi.org/10.13342/j.cnki.cjhp.2014.02.032](https://doi.org/10.13342/j.cnki.cjhp.2014.02.032)

Liu, D. (2013). A study on the relationship among mental health, self-consciousness, and creativity of junior high school students (Master's thesis). Qinghai Normal University. [https://kns.cnki.net/kcms2/article/abstract?v=YtMUyApXHwb-oZ04-ne2roZoObnaN8nAXw2zXkFsR5R-lolLTpSNYbZOMGcWjNogAB4iYWnr3tA1zb_aem-CK6Cie2lAY0zxI4c2leOglxnINHlx7Uc-IFgB5UvWTyJ-OrEdpeziipZCE5vuHxA3TINlKSK4fUug7RAjETE9iaRpN3Ku2ros_g==&uniplatform=NZKPT&language=CHS](https://kns.cnki.net/kcms2/article/abstract?v=YtMUyApXHwb-oZ04-ne2roZoObnaN8nAXw2zXkFsR5R-lolLTpSNYbZOMGcWjNogAB4iYWnr3tA1zb_aem-CK6Cie2lAY0zxI4c2leOglxnINHlx7Uc-IFgB5UvWTyJ-OrEdpeziipZCE5vuHxA3TINlKSK4fUug7RAjETE9iaRpN3Ku2ros_g==&uniplatform=NZKPT&language=CHS)

Liu, D. (2014). A study on the differences in creative personality among industrial design students in Hangzhou universities (Master's thesis). Zhejiang University of Technology. [https://kns.cnki.net/kcms2/article/abstract?v=YtMUyApXHwYgmkUgZ1bFTb8fgVBUt1B4hLjl50Nk13iMzVDi7FW0_SnHSqa0HI7lWo_Yw8PrjhT3EV8BL9QEwk64oxJmfHLxPYIsuV410q5krujzVI9OznHkr1zd4hQj-UdE3RXBdCTcK2z2qTrLEflYEkG-htmfhdP7Cj72RYOo1tw8KtMrlA==&uniplatform=NZKPT&language=CHS](https://kns.cnki.net/kcms2/article/abstract?v=YtMUyApXHwYgmkUgZ1bFTb8fgVBUt1B4hLjl50Nk13iMzVDi7FW0_SnHSqa0HI7lWo_Yw8PrjhT3EV8BL9QEwk64oxJmfHLxPYIsuV410q5krujzVI9OznHkr1zd4hQj-UdE3RXBdCTcK2z2qTrLEflYEkG-htmfhdP7Cj72RYOo1tw8KtMrlA==&uniplatform=NZKPT&language=CHS)

Liu, F. C. (2020). Development and practical research of school-based programming curriculum for primary school oriented to creative thinking cultivation (Master's thesis). Qufu Normal University. [https://doi.org/10.27267/d.cnki.gqfsu.2020.000886](https://doi.org/10.27267/d.cnki.gqfsu.2020.000886)

Liu, H. (2023). Research on strategies for cultivating creativity in primary school students from the perspective of smart education (Master's thesis). Harbin Normal University. [https://doi.org/10.27064/d.cnki.ghasu.2023.001313](https://doi.org/10.27064/d.cnki.ghasu.2023.001313)

Liu, L. (2007). Research on effective ways to improve students' innovative ability in high school politics class (Master's thesis). Tianjin Normal University. [https://kns.cnki.net/kcms2/article/abstract?v=YtMUyApXHwYMsTitu76hTuKT7b0JI3BTePs4QQYdTARqcO5hu3TBnmPnbT4xR7m-pNj_9xWlU10TfT-6ZcHA1u6SkdLXQrDgi7h2dbuHtvaoEP0GMk8jO6qgeC_MW9Xs2kzFx9KfEtw0XlZl1KZY-ih6Xs8sETfLZqKDER65aio-QhZe96IGqg==&uniplatform=NZKPT&language=CHS](https://kns.cnki.net/kcms2/article/abstract?v=YtMUyApXHwYMsTitu76hTuKT7b0JI3BTePs4QQYdTARqcO5hu3TBnmPnbT4xR7m-pNj_9xWlU10TfT-6ZcHA1u6SkdLXQrDgi7h2dbuHtvaoEP0GMk8jO6qgeC_MW9Xs2kzFx9KfEtw0XlZl1KZY-ih6Xs8sETfLZqKDER65aio-QhZe96IGqg==&uniplatform=NZKPT&language=CHS)

Liu, L. (2010). Investigation and analysis of the creativity status of students in a Beijing secondary vocational school (Master’s thesis, Beijing University of Chinese Medicine). China Master’s Theses Full-text Database. [https://kns.cnki.net/kcms2/article/abstract?v=YtMUyApXHwZy4kqSZcdvxR3Mdv6UCGm8duLl4T7mzc6rRx4ggWOHqUVSXOJ1D2w1bs52ob98dQyLPUYm1yswKh4mLMTDv6O0iOBfjmfy5BlYXML3uS7XWYFUu_XD8k6QMG26TQOb1Svaosz2i_1ku0hkTu_Du3xB8ScDk49BZE0HXb5lUgzEtA==&uniplatform=NZKPT&language=CHS](https://kns.cnki.net/kcms2/article/abstract?v=YtMUyApXHwZy4kqSZcdvxR3Mdv6UCGm8duLl4T7mzc6rRx4ggWOHqUVSXOJ1D2w1bs52ob98dQyLPUYm1yswKh4mLMTDv6O0iOBfjmfy5BlYXML3uS7XWYFUu_XD8k6QMG26TQOb1Svaosz2i_1ku0hkTu_Du3xB8ScDk49BZE0HXb5lUgzEtA==&uniplatform=NZKPT&language=CHS)

Liu, M. H. (2020). A correlational and intervention study on self-control, creativity, and academic achievement in adolescents (Master's thesis). Bohai University. [https://doi.org/10.27190/d.cnki.gjzsc.2020.000123](https://doi.org/10.27190/d.cnki.gjzsc.2020.000123)

Liu, M. Y., & Zhang, H. (2017). The current situation and differences of creative tendency in adolescents. Innovation and Entrepreneurship Education, 8(2), 116-119.

Liu, P. (2008). A study on the correlation between creativity tendency and learning style of nursing secondary vocational school students (Master's thesis). Shanxi Medical University. [https://kns.cnki.net/kcms2/article/abstract?v=YtMUyApXHwbN0gUz7xVkiyMpPqdQunIHx-HvNcAxvs9x1dJ7bqCOJYgmqNmDZsOHVIRWOSKvYbP4v_Cw89_ZCtpvtNJKn7XrIrg6M4ZpIlaAQBcPhXqWUdLRskPCrF1WVaL5TKNYG5pGsOylJc9UYSs_7RLQUZXjjDyx1UrMVtlFqcFJgGPGUQ==&uniplatform=NZKPT&language=CHS](https://kns.cnki.net/kcms2/article/abstract?v=YtMUyApXHwbN0gUz7xVkiyMpPqdQunIHx-HvNcAxvs9x1dJ7bqCOJYgmqNmDZsOHVIRWOSKvYbP4v_Cw89_ZCtpvtNJKn7XrIrg6M4ZpIlaAQBcPhXqWUdLRskPCrF1WVaL5TKNYG5pGsOylJc9UYSs_7RLQUZXjjDyx1UrMVtlFqcFJgGPGUQ==&uniplatform=NZKPT&language=CHS)

Liu, S. Q. (2020). Investigation and cultivation of middle school students' creative thinking under STEM education (Master's thesis). Hunan Normal University. [https://doi.org/10.27137/d.cnki.ghusu.2020.000874](https://doi.org/10.27137/d.cnki.ghusu.2020.000874)

Liu, W. S. (2023). Design and practice research of graphical programming learning activities in primary school oriented to creative thinking cultivation (Master's thesis). Northwest Normal University. [https://doi.org/10.27410/d.cnki.gxbfu.2023.001081](https://doi.org/10.27410/d.cnki.gxbfu.2023.001081)

Liu, X. (2011). *A correlational study on creativity, self-efficacy, and mathematics achievement among high school students* (Master's thesis). Henan University. https://kns.cnki.net/kcms2/article/abstract?v=YtMUyApXHwZ-HePZ36ZtAu2rtNyKAtW9Ws7Q2hZtcXC1Cjb7Verxukg9F9LqlReGy6Y5bEPrTY14jQJa7i46qh7qOAydGVtSXoAGBuQlgpqLs7NLQ9OtwHnyZUPFqklZCv3Eud-FwPo-fodQSeX7-e8jqDOILX4z5Mej7JPxbFDnMkfUYdiJkw==&uniplatform=NZKPT&language=CHS

Liu, X. H., & Shan, Y. (2013). Practice of interactive teaching in cultivating creativity of nursing undergraduates. Health Vocational Education, 31(14), 109-110.

Liu, X. L. (2016). A study on the relationship between creative tendency and personality traits in high school students. Shanghai Journal of Educational Research, (04), 34-36. [https://doi.org/10.16194/j.cnki.31-1059/g4.2016.04.009](https://doi.org/10.16194/j.cnki.31-1059/g4.2016.04.009)

Liu, X. L., Liu, L., Qiu, Y. X., Jin, Y., & Zhou, J. (2016). Reliability and validity test of the Williams Creativity Assessment Packet. Basic Education, 13(3), 51-58.

Liu, Y. (2020). Research on the cultivation of college students' creativity in knowledge system learning (Master's thesis). Shanghai Normal University. [https://doi.org/10.27312/d.cnki.gshsu.2020.000601](https://doi.org/10.27312/d.cnki.gshsu.2020.000601)

Liu, Y. X. (2023). Research on the enhancement of creativity in junior high school students (Master's thesis). Zhejiang Normal University. [https://doi.org/10.27464/d.cnki.gzsfu.2023.002106](https://doi.org/10.27464/d.cnki.gzsfu.2023.002106)

Liu, Y. Y., Yang, D. H., & Li, X. L. (2011). A correlational study on locus of control and creativity tendency among senior three high school students. In Proceedings of the 2011 International Conference on Future Computer Science and Application (FCSA 2011 V4) (pp. 242–245). School of Education, China West Normal University.

Liu, Z. C. ./(2020). Research on the construction of Scratch curriculum teaching mode for cultivating innovative ability in primary and secondary schools (Master's thesis). Liaoning Normal University. [https://doi.org/1-0.27212/d.cnki.glnsu.2020.000190](https://doi.org/10.27212/d.cnki.glnsu.2020.000190)

Lou, S. J., Chou, Y. C., Shih, R. C., & Chung, C. C. (2017). A study of creativity in CaC2 steamship-derived STEM project-based learning. *Eurasia Journal of Mathematics, Science and Technology Education*, 13(6), 2387-2404.

Lu, S. Y., Lo, C. C., & Syu, J. Y. (2022). Project-based learning oriented STEAM: The case of micro–bit paper-cutting lamp. *International Journal of Technology and Design Education*, 32(5), 2553-2575.

Luo, M. (2020). Research on the impact of STEM curriculum on the innovative ability and engineering thinking of fourth-grade students (Master's thesis). Central China Normal University. [https://doi.org/10.27159/d.cnki.ghzsu.2020.004082](https://doi.org/10.27159/d.cnki.ghzsu.2020.004082)

Luo, X. L. (2010). The developmental characteristics and interrelationship of college students' creativity and mental health. In Twenty years of "heart" exploration: Research on theory and methods of university mental health education (pp. 481–484). Beijing Normal University Psychological Counseling and Research Center.

Lv Y. (2016). A study on the factors influencing college students' innovative ability from the perspective of basic education stage. Technology and Innovation Management, 37(6), 693-697. [https://doi.org/10.14090/j.cnki.jscx.2016.0619](https://doi.org/10.14090/j.cnki.jscx.2016.0619)

Lv, J. (2016). *Developmental characteristics of middle school students’ creative thinking and its relationship with creative personality and academic achievement (Master’s thesis).* University of Jinan. https://kns.cnki.net/kcms2/article/abstract?v=iO0L29morZmEyC23QNa9jZGgIn5E73AKNAm_2_TnDr5lC3dkRRlRnRygzg9pKWQh2A2m4S4gYMM6EpeSJ8SPf2ZlKwLqGeunSxsjLg-5EAm3Eld3AY3l9BUmKuYmgLFi4YqIHVrUmX2f1woSumUNxglH_oX-WVK7UTi99ic6R7dMWQKapO4X1Q==&uniplatform=NZKPT&language=CHS

Lv, W. W. (2024). A practical study on cultivating students’ creative thinking in senior high school biology under the TFU teaching model (Master’s thesis). Yan’an University. https://doi.org/10.27438/d.cnki.gyadu.2024.000972

Ma, R. R. (2024). The influence of creative classroom environment on creative tendency in high school students (Master's thesis). Yunnan Normal University. [https://doi.org/10.27459/d.cnki.gynfc.2024.001162](https://doi.org/10.27459/d.cnki.gynfc.2024.001162)

Meng, L. (2021). Research on the design of robot course learning activities based on activity theory (Master's thesis). Inner Mongolia Normal University. [https://doi.org/10.27230/d.cnki.gnmsu.2021.000335](https://doi.org/10.27230/d.cnki.gnmsu.2021.000335)

Meng, Z. W. (2021). Research on the cultivation of students' creativity through junior high school Mixly curriculum under the concept of maker education (Master's thesis). Harbin Normal University. [https://doi.org/10.27064/d.cnki.ghasu.2021.000937](https://doi.org/10.27064/d.cnki.ghasu.2021.000937)

Mu, X. J. (2016). An empirical study on the influence of digital educational games on the development of creative thinking in middle school students (Master's thesis). Shaanxi Normal University. [https://kns.cnki.net/kcms2/article/abstract?v=YtMUyApXHway0UtgmXaYQSZQ4DmMO9RnjioHy5Ec4WQhIw0BiB7gBNk7je2A2u1dEjjBKj81q-w2zeNUWpovC88s6pamU08FckgcT4EKPpcMgGo0TKwAEnF2Q9dds7ZXOJvquMMJjE37POq3Jr-YdlJ8b7iK6Hbcwvv2zCY4FbLvpJY8Z74cfw==&uniplatform=NZKPT&language=CHS](https://kns.cnki.net/kcms2/article/abstract?v=YtMUyApXHway0UtgmXaYQSZQ4DmMO9RnjioHy5Ec4WQhIw0BiB7gBNk7je2A2u1dEjjBKj81q-w2zeNUWpovC88s6pamU08FckgcT4EKPpcMgGo0TKwAEnF2Q9dds7ZXOJvquMMJjE37POq3Jr-YdlJ8b7iK6Hbcwvv2zCY4FbLvpJY8Z74cfw==&uniplatform=NZKPT&language=CHS)

Ni, Y. Y. (2022). An action research on the impact of the CDIO-PBL teaching model on high school students' creative thinking (Master's thesis). Hangzhou Normal University. [https://doi.org/10.27076/d.cnki.ghzsc.2022.000748](https://doi.org/10.27076/d.cnki.ghzsc.2022.000748)

Nie, Y. G., & Zheng, X. (2005). A study on the developmental characteristics of creative personality in children and adolescents. Psychological Science, (02), 356-361. [https://doi.org/10.16719/j.cnki.1671-6981.2005.02.024](https://doi.org/10.16719/j.cnki.1671-6981.2005.02.024)

Niu, H. Y. (2019). Investigation and intervention study on creative personality, entrepreneurial self-efficacy, and entrepreneurial intention of secondary vocational school students (Master's thesis). Hebei Normal University. [https://doi.org/10.27110/d.cnki.ghsfu.2019.000098](https://doi.org/10.27110/d.cnki.ghsfu.2019.000098)

Niu, L. L. (2021). Design of learning activities supported by the EDIPT design thinking model to foster students' innovative abilities (Master's thesis). Shandong Normal University. [https://doi.org/10.27280/d.cnki.gsdsu.2021.001146](https://doi.org/10.27280/d.cnki.gsdsu.2021.001146)

Niu, X. Y. (2022). Group sandplay intervention and curriculum design for creative tendency in junior high school students (Master's thesis). Mudanjiang Normal University. [https://doi.org/10.27757/d.cnki.gmdjs.2022.000196](https://doi.org/10.27757/d.cnki.gmdjs.2022.000196)

Ouyang, L. (2016). A study on the influence of creative personality and entrepreneurship education on college students' entrepreneurial intention (Master's thesis). Changsha University of Science & Technology. [https://kns.cnki.net/kcms2/article/abstract?v=YtMUyApXHwZC_e-Y_Hq_vNPkTxIoJ-lOOhjoZXBdgvVmw08ZqdkJljELST0IYTD-bmZR0SR61YeQYxt0EonNrhattMl44FsVFF2pJBaNCyYnpkvdktXlwtsGvGvCJ-rGJR1IkSLdS1R7QnpnUMtFUUU659tYIbM3gr30ax45veu-fqKJKkzLuw==&uniplatform=NZKPT&language=CHS](https://kns.cnki.net/kcms2/article/abstract?v=YtMUyApXHwZC_e-Y_Hq_vNPkTxIoJ-lOOhjoZXBdgvVmw08ZqdkJljELST0IYTD-bmZR0SR61YeQYxt0EonNrhattMl44FsVFF2pJBaNCyYnpkvdktXlwtsGvGvCJ-rGJR1IkSLdS1R7QnpnUMtFUUU659tYIbM3gr30ax45veu-fqKJKkzLuw==&uniplatform=NZKPT&language=CHS)

Pan, Y. B., Xu, X. Y., Wu, X. Y., Yan, H. X., & Gu, Y. (2016). Practical research on creativity cultivation of five-year higher vocational nursing students. Science & Technology Information, 14(26), 174-177. [https://doi.org/10.16661/j.cnki.1672-3791.2016.26.174](https://doi.org/10.16661/j.cnki.1672-3791.2016.26.174)

Qian, L. L. (2013). A study on the relationship among superstitious psychology, creative tendency, and critical thinking tendency in adolescents (Master's thesis). Nanjing University. [https://kns.cnki.net/kcms2/article/abstract?v=YtMUyApXHwZ1jwMVjQ_hS5hVPlJU4NFuqVXBiKhQD4mlYWI4ovyqEuF3Pr6osrTGlkRAh2rA9FoPVPov4A03s2-DxDirxRMYmOZDTiqp1sK1TY79AdeFT9EGqBz8x2rUcvcfkikHq1YvvX54kuiA4IHFw_kd5qGA-iud7VHFyQh5bNm9D_kxOQ==&uniplatform=NZKPT&language=CHS](https://kns.cnki.net/kcms2/article/abstract?v=YtMUyApXHwZ1jwMVjQ_hS5hVPlJU4NFuqVXBiKhQD4mlYWI4ovyqEuF3Pr6osrTGlkRAh2rA9FoPVPov4A03s2-DxDirxRMYmOZDTiqp1sK1TY79AdeFT9EGqBz8x2rUcvcfkikHq1YvvX54kuiA4IHFw_kd5qGA-iud7VHFyQh5bNm9D_kxOQ==&uniplatform=NZKPT&language=CHS)

Qian, S. Y. (2007). A study on the relationship between cognitive style and creative tendency. Journal of Huainan Normal University, (02), 114-116.

Qian, X. (2009). Research on cultivating students' innovative ability using the STS education model in junior high school physics teaching (Master's thesis). Soochow University. [https://kns.cnki.net/kcms2/article/abstract?v=YtMUyApXHwbzA40e5IZixRWXxtTB5jqwNQGhOYG8TFS85NafLe8pNTEzFP_ntJCqrIqsfiQX99fIdzW5lbCa9rAQNjD57th-CtRay6Xz2XR6t7GDy0UepfzdS9vxPO9PWG0-9YjhSTi_Kq2nO6f-QhEmUjWFCIhFDIf0gFmsUC_ym_sqlnKeNQ==&uniplatform=NZKPT&language=CHS](https://kns.cnki.net/kcms2/article/abstract?v=YtMUyApXHwbzA40e5IZixRWXxtTB5jqwNQGhOYG8TFS85NafLe8pNTEzFP_ntJCqrIqsfiQX99fIdzW5lbCa9rAQNjD57th-CtRay6Xz2XR6t7GDy0UepfzdS9vxPO9PWG0-9YjhSTi_Kq2nO6f-QhEmUjWFCIhFDIf0gFmsUC_ym_sqlnKeNQ==&uniplatform=NZKPT&language=CHS)

Qin, Y. Q. (2019). Research on the relationship between parenting styles, creative self-efficacy, and creative tendency among college students (Master's thesis). Nanchang University. [https://kns.cnki.net/kcms2/article/abstract?v=YtMUyApXHwb1psXkohUQy_KGdxIoRLoQN9Mp5rm-8TSaXJ5ateWye_v_HXHNovp8_DFOs5jd2aND1I19OkYRrUVE_xLflfFj_XgJmfZghDYstVnWB-hRG1hUhow4FyaqPxLPc4uAAvVuQFvf9wUJpt95WTghPu0135sqdMnAdQuZTIaKVE7Kxw==&uniplatform=NZKPT&language=CHS](https://kns.cnki.net/kcms2/article/abstract?v=YtMUyApXHwb1psXkohUQy_KGdxIoRLoQN9Mp5rm-8TSaXJ5ateWye_v_HXHNovp8_DFOs5jd2aND1I19OkYRrUVE_xLflfFj_XgJmfZghDYstVnWB-hRG1hUhow4FyaqPxLPc4uAAvVuQFvf9wUJpt95WTghPu0135sqdMnAdQuZTIaKVE7Kxw==&uniplatform=NZKPT&language=CHS)

Qiu, C. (2020). Research on maker teaching with 3D printing for cultivating creative thinking (Master's thesis). Shaanxi Normal University. [https://doi.org/10.27292/d.cnki.gsxfu.2020.001709](https://doi.org/10.27292/d.cnki.gsxfu.2020.001709)

Ren, F., Liu, Y., Wang, Y. X., & Chen, G. X. (2020). The relationship between working memory capacity and the continued influence effect of misinformation: The mediating role of creativity. *Psychological Techniques and Applications,* *8*(10), 604–610. https://doi.org/10.16842/j.cnki.issn2095-5588.2020.10.004

Shen, X. F. (2024). Research on the impact of primary school art education projects under the concept of STEAM on students' creative thinking ability and creative tendency. Art Education Research, (21), 84–88.

Sheng, S. X. (2018). *A behavioral study on the cultivation of creativity among rural children integrated into elementary school mathematics classroom teaching* (Master’s thesis, Minnan Normal University). Retrieved from https://kns.cnki.net/kcms2/article/abstract?v=VYuoLtjwl8P3Z4Jdx9uYaWRCRfGK23byHD9ZT8hfakx4ui4CumXeQd45ovOe8_ITRcTNaGBzg_2oHUBjB2_wsHZvWAeWsiKjFPWuFZ8xue-eL-4TzsXsKtHxwDHHD20Bl0uCmlzT-7bVC-xV1y_E9LSMiVr76GMBHw8rkLBtmsm5HqyHMcYRtQ==

Shi, B. G., & Xu, J. J. (2009). The predictive role of family environment on college students' creative tendency. Journal of Capital Normal University (Social Sciences Edition), (05), 71-77.

Shi, W. W. (2006). The relationship among motivation arousal types, motivation regulation methods, and creativity (Master's thesis). Shandong Normal University. [https://kns.cnki.net/kcms2/article/abstract?v=YtMUyApXHwagvyy5VPlYEj8HXWgDY4dF_s-KM7GS3PAvBm91I8KDj4Am51QUAkRDwq2IykjTk51gMHj1ywaJkcNlvAX17OGSB3zgv3wZbXBwCE9KfsaGdQTIffcPwlbO6V26WgSGVDENCLaEMeVla7FfzC0zv0oBq_WpljfWadnv6BxcFsGH-w==&uniplatform=NZKPT&language=CHS](https://kns.cnki.net/kcms2/article/abstract?v=YtMUyApXHwagvyy5VPlYEj8HXWgDY4dF_s-KM7GS3PAvBm91I8KDj4Am51QUAkRDwq2IykjTk51gMHj1ywaJkcNlvAX17OGSB3zgv3wZbXBwCE9KfsaGdQTIffcPwlbO6V26WgSGVDENCLaEMeVla7FfzC0zv0oBq_WpljfWadnv6BxcFsGH-w==&uniplatform=NZKPT&language=CHS)

Song, Z. Y., Zhu, H. Y., & Zhang, F. (2005). A study on personality characteristics of college students with different creative tendencies. China Journal of Health Psychology, (04), 241–244. [https://doi.org/10.13342/j.cnki.cjhp.2005.04.001](https://doi.org/10.13342/j.cnki.cjhp.2005.04.001)

Su, H. (2024). Practical research on cultivating creative thinking in junior high school students using the history of biological science (Master's thesis). Yan’an University. [https://doi.org/10.27438/d.cnki.gyadu.2024.000983](https://doi.org/10.27438/d.cnki.gyadu.2024.000983)

Su, Y. (2010). A study on the correlation between social anxiety and creative tendency among 223 primary school students. Chinese Journal of School Doctor, 24(5), 348–350.

Sun, F. (2021). Test and strategy research of creative thinking ability in primary school students from a psychological perspective (Master's thesis). Qinghai Normal University. [https://doi.org/10.27778/d.cnki.gqhzy.2021.000520](https://doi.org/10.27778/d.cnki.gqhzy.2021.000520)

Sun, S. J. (2020). A study on the relationship between self-construal and creativity in junior high school students: The effect of creative self-efficacy (Master's thesis). Southwest University. [https://doi.org/10.27684/d.cnki.gxndx.2020.004375](https://doi.org/10.27684/d.cnki.gxndx.2020.004375)

Tan, J. (2024). A practical study on cultivating creative thinking through the CPS model in high school biology teaching (Master's thesis). Minnan Normal University. [https://doi.org/10.27726/d.cnki.gzzsf.2024.000572](https://doi.org/10.27726/d.cnki.gzzsf.2024.000572)

Tang, Y. (2024). The relationship between creative personality and scientific literacy in primary school students and its educational countermeasures (Master's thesis). Southwest University. [https://doi.org/10.27684/d.cnki.gxndx.2024.000143](https://doi.org/10.27684/d.cnki.gxndx.2024.000143)

Teng, H. (2014). Research on the relationship among entrepreneurial intention, creative personality, and social support of college students in Chongqing (Master's thesis). Southwest University. [https://kns.cnki.net/kcms2/article/abstract?v=YtMUyApXHwYYlcgqmPefdYgZDbjmaCUgDzVrFU1dO7magUyp_DqliVy883rcz03fKWUqxVBA9PzRo11mi60EvZJq0sBHwVXeemBA2NORppi9KB2Bw20UZcSd_6otoIcWPHuzc4O4W_DgIuo9Ki94v-9dbi9ShbvW_9OfM3jf-YwotIDAHKA5Zw==&uniplatform=NZKPT&language=CHS](https://kns.cnki.net/kcms2/article/abstract?v=YtMUyApXHwYYlcgqmPefdYgZDbjmaCUgDzVrFU1dO7magUyp_DqliVy883rcz03fKWUqxVBA9PzRo11mi60EvZJq0sBHwVXeemBA2NORppi9KB2Bw20UZcSd_6otoIcWPHuzc4O4W_DgIuo9Ki94v-9dbi9ShbvW_9OfM3jf-YwotIDAHKA5Zw==&uniplatform=NZKPT&language=CHS)

Tian, Q. (2022). *A study on the relationship between exercise habits, creativity, mental health and academic achievement of middle school students (Master’s thesis)*. Southwest University. https://doi.org/10.27684/d.cnki.gxndx.2022.003036

Tong, H. (2023). The impact of high-intensity interval training on creative thinking and its underlying mechanisms (Master's thesis). Southwest University. [https://doi.org/10.27684/d.cnki.gxndx.2023.002974](https://doi.org/10.27684/d.cnki.gxndx.2023.002974)

Tong, X. F. (2019). Research on the cultivation of college students' creativity from the perspective of mindfulness theory (Master's thesis). Northeast Petroleum University. [https://doi.org/10.26995/d.cnki.gdqsc.2019.000250](https://doi.org/10.26995/d.cnki.gdqsc.2019.000250)

Wang, C. M., & Shu, D. M. (2013). A study on scientific creativity and creative personality of vocational school students. Journal of Nantong University (Social Sciences Edition), 29(5), 130-135.

Wang, C. M., & Wang, M. (2014). The development status and countermeasures of creative personality among students in local universities: A case study of Nantong University. University Education Management, 8(2), 112-116. [https://doi.org/10.13316/j.cnki.jhem.2014.02.022](https://doi.org/10.13316/j.cnki.jhem.2014.02.022)

Wang, H. Q., Kuang, Z. H., Wang, Q. S., & Ju, L. K. (2005). Investigation and analysis of the overall status of college students' innovative ability. Journal of Higher Education, (09), 88-93.

Wang, J. (2021). Intervention study on promoting mental health level and academic achievement of middle school students through creativity (Master's thesis). Southwest University. [https://doi.org/10.27684/d.cnki.gxndx.2021.000842](https://doi.org/10.27684/d.cnki.gxndx.2021.000842)

Wang, L. Y., Li, X. P., & Zhang, J. X. (2006). A study on the characteristics of creative tendency among college students. Health Medicine Research and Practice in Higher Institutions, (03), 14-17.

Wang, L., Liu, H. G., Hou, R. X., & Yang, H. Y. (2012). A study on the relationship between college students’ learning motivation and the internal factors of creativity tendency. *Journal of Yangzhou Polytechnic College, 16(2),* 58–62. <https://doi.org/10.15954/j.cnki.cn32-1529/g4.2012.02.014>

Wang, P. (2020). A study on the relationship among college students' physical education self-directed learning ability, cognitive need, and creativity. Talent and Wisdom, (20), 64-65.

Wang, S. Y. (2008). A study on the relationship among metacognition, creative personality, and creative thinking in college students (Master's thesis). Northwest University. [https://kns.cnki.net/kcms2/article/abstract?v=YtMUyApXHwbHEoJDD070S5MOvI4pxrNfrkiAU9fJ30dokExaRauPvtlm3VPNe-d4TYPsg3Wo4IlI3Sa_8zt3qi0beur8bCqfs3ciP2_fOaxq16i4qpsmvh1n7INheAcuIGnUnwNcaGnMRCGl_xBUPpg4dh155SXzoyI6xKkVKMRj1ydy5xC5Hw==&uniplatform=NZKPT&language=CHS](https://kns.cnki.net/kcms2/article/abstract?v=YtMUyApXHwbHEoJDD070S5MOvI4pxrNfrkiAU9fJ30dokExaRauPvtlm3VPNe-d4TYPsg3Wo4IlI3Sa_8zt3qi0beur8bCqfs3ciP2_fOaxq16i4qpsmvh1n7INheAcuIGnUnwNcaGnMRCGl_xBUPpg4dh155SXzoyI6xKkVKMRj1ydy5xC5Hw==&uniplatform=NZKPT&language=CHS)

Wang, T. Q., & Pang, W. H. (2023). Current situation and optimization strategies of creativity tendency among master's students. Heilongjiang Science, 14(3), 77-81.

Wang, X. L., Zhang, J. H., Chu, Y. X., & Liu, G. R. (2009). The relationship among family environment, creative self-efficacy, and creativity in primary school children. Psychological Exploration, 29(5), 46-50+66.

Wang, X. T. (2010). Practice and exploration of cultivating middle school students' technological innovation ability through science and technology project guidance (Master's thesis). Northeast Normal University. [https://kns.cnki.net/kcms2/article/abstract?v=YtMUyApXHwZQmbKLoV7mTJ2sX7zzCVgi6iDr1TMHWzt1FouhkayrkrNJCdxTF3ryDhvtESpcMy1KHyfLGvayzCK0dc_Nrt87PWUWPeTuL2CZPgbrsY1KmRiE8pjPwmtq5YssjVDdrs7d6jiTb8EzhizSs7vFCy3zp4Gdsa7gpmQvxa5XODpiCA==&uniplatform=NZKPT&language=CHS](https://kns.cnki.net/kcms2/article/abstract?v=YtMUyApXHwZQmbKLoV7mTJ2sX7zzCVgi6iDr1TMHWzt1FouhkayrkrNJCdxTF3ryDhvtESpcMy1KHyfLGvayzCK0dc_Nrt87PWUWPeTuL2CZPgbrsY1KmRiE8pjPwmtq5YssjVDdrs7d6jiTb8EzhizSs7vFCy3zp4Gdsa7gpmQvxa5XODpiCA==&uniplatform=NZKPT&language=CHS)

Wang, X. X. (2012). Research on the application of Scratch in middle school programming teaching under network environment (Master's thesis). Southwest University. [https://kns.cnki.net/kcms2/article/abstract?v=YtMUyApXHwYoTAnauQ8gZi1YtaahHcB7RGkGvbd0WeS8eVeTPg7PDm9ZiVUK4UxjAzvuLvEM3YiCQLcXr0ALnCKRvTmIA260Z6kMwoEkq3mpzm3zIZOch_QAYCq0cwYK45BaA0PXFIRfbffvqkoAN7qKr8S-XI8oUIfb5b_8AOKf6CFsFFEfRg==&uniplatform=NZKPT&language=CHS](https://kns.cnki.net/kcms2/article/abstract?v=YtMUyApXHwYoTAnauQ8gZi1YtaahHcB7RGkGvbd0WeS8eVeTPg7PDm9ZiVUK4UxjAzvuLvEM3YiCQLcXr0ALnCKRvTmIA260Z6kMwoEkq3mpzm3zIZOch_QAYCq0cwYK45BaA0PXFIRfbffvqkoAN7qKr8S-XI8oUIfb5b_8AOKf6CFsFFEfRg==&uniplatform=NZKPT&language=CHS)

Wang, X. Z., & Zhu, L. Y. (2017). Analysis of college students' high creativity tendency: Based on a survey of nine universities in Guangzhou Higher Education Mega Center. Innovation Science and Technology, (04), 30-34. [https://doi.org/10.19345/j.cnki.1671-0037.2017.04.009](https://doi.org/10.19345/j.cnki.1671-0037.2017.04.009)

Wang, Y. (2001). An investigation on the relationship between cognitive style and creativity in primary school children. Journal of Zhejiang Ocean University (Humanities Sciences), (04), 82-85.

Wang, Y. (2009). Research on the development, related abilities and influencing factors of informal reasoning ability in middle school students (Master's thesis). Liaoning Normal University. [https://kns.cnki.net/kcms2/article/abstract?v=lSOmZDqoX8P5Ulbwkqnbjli28AKWfTAPDu7RkBHlH0vKmixowVSLiOw4UbfUFEoeSMGl2UCWa3aRoDaCVuicM7v2wIfngdC84107sJo-G4vBJeBMxMKXJIBAOc6BQlt-xpRrnF_q57v6dxYgsg7-AHyzlvLgoFetTvrD1cIK8RAbkszX1hfnlA==&uniplatform=NZKPT&language=CHS](https://kns.cnki.net/kcms2/article/abstract?v=lSOmZDqoX8P5Ulbwkqnbjli28AKWfTAPDu7RkBHlH0vKmixowVSLiOw4UbfUFEoeSMGl2UCWa3aRoDaCVuicM7v2wIfngdC84107sJo-G4vBJeBMxMKXJIBAOc6BQlt-xpRrnF_q57v6dxYgsg7-AHyzlvLgoFetTvrD1cIK8RAbkszX1hfnlA==&uniplatform=NZKPT&language=CHS)

Wang, Y. D. (2023). Construction and practice research of PBL teaching model oriented to creative thinking cultivation (Master's thesis). East China Normal University. [https://doi.org/10.27149/d.cnki.ghdsu.2023.002646](https://doi.org/10.27149/d.cnki.ghdsu.2023.002646)

Wang, Y. L. (2024). Action research on cultivating creative thinking under the concept of STEAM (Master's thesis). Minnan Normal University. [https://doi.org/10.27726/d.cnki.gzzsf.2024.000409](https://doi.org/10.27726/d.cnki.gzzsf.2024.000409)

Wang, Y. Y. (2020). Research on the developmental characteristics and influencing factors of creativity in middle and upper primary school students (Master's thesis). Yanbian University. [https://doi.org/10.27439/d.cnki.gybdu.2020.000605](https://doi.org/10.27439/d.cnki.gybdu.2020.000605)

Wang, Z. Y. (2009). Investigation, analysis and development research on creative personality of college students in China (Master's thesis). Lanzhou University. [https://kns.cnki.net/kcms2/article/abstract?v=YtMUyApXHwaIv2s3KtSPu2TI2lYy_dKhgNwTl_I0WdbuKiNuaZ7-mpocJSSrjQx7d6gYMe1z5bodCktq9nAyYbjJMbWbe3Kq1WDnFhW-LamIRj2T0glL1KbWP720vvSKyA1oTHDgGoum59KuuXanyLozM-yuoPMwI7WBT1e4M8ZDJ3RzutC5sw==&uniplatform=NZKPT&language=CHS](https://kns.cnki.net/kcms2/article/abstract?v=YtMUyApXHwaIv2s3KtSPu2TI2lYy_dKhgNwTl_I0WdbuKiNuaZ7-mpocJSSrjQx7d6gYMe1z5bodCktq9nAyYbjJMbWbe3Kq1WDnFhW-LamIRj2T0glL1KbWP720vvSKyA1oTHDgGoum59KuuXanyLozM-yuoPMwI7WBT1e4M8ZDJ3RzutC5sw==&uniplatform=NZKPT&language=CHS)

Wei, J. (2022). Applied research on teaching "blank-leaving" strategy for cultivating primary school students' creative thinking (Master's thesis). Northwest Normal University. [https://doi.org/10.27410/d.cnki.gxbfu.2022.001284](https://doi.org/10.27410/d.cnki.gxbfu.2022.001284)

Wu, P., Qi, R., Guo, L., Shen, C. N., & Zhang, Z. (2022). Investigation on creativity cultivation among medical students. China Educational Technology & Equipment, (15), 17-20+37.

Wu, S. (2018). *Development and practice of primary school micro-film school-based curriculum based on cultivation of creative thinking* (Master’s thesis, Shanghai Normal University). Retrieved from https://kns.cnki.net/kcms2/article/abstract?v=VYuoLtjwl8OxGVWQ-1QkabuqpW-Y-p4lS-kltFR_3LApC_Y9KfNV_72WO8aRfHuJux1KKGGDMyNPNYEL_5IxUZ2zJvr71zFjyM2xA7NmF8IfWKe7cD7fAzpcxZIt4aA0PlEc9CRTAwS0UACDjrFoyfzkr8BlHxCNXBlJUc5ODGlS1RhtYsBd6w==

Wu, W. D. (2009). A study on the development of creative tendency from primary to junior middle school students. Journal of Shijiazhuang University, 11(3), 81-85. [https://doi.org/10.13573/j.cnki.sjzxyxb.2009.03.013](https://doi.org/10.13573/j.cnki.sjzxyxb.2009.03.013)

Wu, W. D., & Zhang, X. F. (2006). A study on students' creativity during the transition from primary to middle school. Journal of Dezhou University, (03), 58-60.

Wu, W. R. (2023). Design and practical research of junior high school art curriculum based on STEAM concept to cultivate creativity (Master's thesis). Northwest Minzu University. [https://doi.org/10.27408/d.cnki.gxmzc.2023.000544](https://doi.org/10.27408/d.cnki.gxmzc.2023.000544)

Xiao, Z. H. (2017). The relationship between meta-emotion and creative tendency of left-behind junior high school students: The mediating role of innovative self-efficacy (Master's thesis). Sichuan Normal University. [https://kns.cnki.net/kcms2/article/abstract?v=YtMUyApXHwZiASilBVy9YenvlBvnDa00OizkGkMSwR6rAlwVgNnbGshfaeRxyIdh1F_nUJb5LMLy47tnQJg6TpoplHhYb8fZT8Ra8K5yUzqCnOGn_zEDDf11ZyO0YtVhVF5f8afEtUbs_zrXHoZNLfzzhPJP7ysg67-cMlPnsQU6wfjB6qyI7w==&uniplatform=NZKPT&language=CHS](https://kns.cnki.net/kcms2/article/abstract?v=YtMUyApXHwZiASilBVy9YenvlBvnDa00OizkGkMSwR6rAlwVgNnbGshfaeRxyIdh1F_nUJb5LMLy47tnQJg6TpoplHhYb8fZT8Ra8K5yUzqCnOGn_zEDDf11ZyO0YtVhVF5f8afEtUbs_zrXHoZNLfzzhPJP7ysg67-cMlPnsQU6wfjB6qyI7w==&uniplatform=NZKPT&language=CHS)

Xie, L. P. (2021). An experimental study on the impact of non-written mathematics homework on the creative tendency of lower-grade students (Master's thesis). Bohai University. [https://doi.org/10.27190/d.cnki.gjzsc.2021.000729](https://doi.org/10.27190/d.cnki.gjzsc.2021.000729)

Xiong, X. P. (2021). An empirical study on the impact of biology-themed teaching based on science fiction films on creativity (Master's thesis). Hangzhou Normal University. [https://doi.org/10.27076/d.cnki.ghzsc.2021.000534](https://doi.org/10.27076/d.cnki.ghzsc.2021.000534)

Xu, J. C. (2014). Analysis of the current situation and countermeasures for cultivating innovative ability of higher vocational college students. Industry and Technology Forum, (23), 88-91.

Xu, W. W., Yang, G., Zhu, M. R., Chen, J. T., & Li, W. F. (2019). The relationship between psychological resilience and creativity in college students: The mediating role of cognitive flexibility. China Journal of Health Psychology, 27(12), 1885–1890. [https://doi.org/10.13342/j.cnki.cjhp.2019.12.034](https://doi.org/10.13342/j.cnki.cjhp.2019.12.034)

Xu, Y. X. (2017). Does mindfulness promote creativity? — The different effects of observation and concentration (Master's thesis). Southwest University. [https://kns.cnki.net/kcms2/article/abstract?v=YtMUyApXHwYwbeBXZCSgdmwBMxC2zNt034Oi965oV8Qs8ssJ4PbIJYaKc6hbOhcgsGZM9bYUqGVC2bWnzpq2GhKDeGQ_A1a40rE9EIxlrzjZDQyUAWofp1sqttVofMdvehLgNkNKZSB5z5wOhIZTNXBVo1_wiW6VtokH_xwkw9yaUJq01wm5nQ==&uniplatform=NZKPT&language=CHS](https://kns.cnki.net/kcms2/article/abstract?v=YtMUyApXHwYwbeBXZCSgdmwBMxC2zNt034Oi965oV8Qs8ssJ4PbIJYaKc6hbOhcgsGZM9bYUqGVC2bWnzpq2GhKDeGQ_A1a40rE9EIxlrzjZDQyUAWofp1sqttVofMdvehLgNkNKZSB5z5wOhIZTNXBVo1_wiW6VtokH_xwkw9yaUJq01wm5nQ==&uniplatform=NZKPT&language=CHS)

Xu, Z. L. (2023). A study on the influence of creative personality and mathematics learning engagement on mathematical modeling ability in high school students (Master's thesis). Yunnan Normal University. [https://doi.org/10.27459/d.cnki.gynfc.2023.000883](https://doi.org/10.27459/d.cnki.gynfc.2023.000883)

Xue, L. Y. (2020). A study on the impact of teachers' transformational leadership style on the creative tendency of junior high school students (Master's thesis). Shanghai Jiao Tong University. [https://doi.org/10.27307/d.cnki.gsjtu.2020.003891](https://doi.org/10.27307/d.cnki.gsjtu.2020.003891)

Yan, J. (2024). A study on the influence of integrated situational teaching on students' creative thinking in high school biology (Master's thesis). Guizhou Normal University. [https://doi.org/10.27048/d.cnki.ggzsu.2024.000053](https://doi.org/10.27048/d.cnki.ggzsu.2024.000053)

Yang, L. (2020). Analysis of the current situation and influencing factors of creativity tendency among medical academic master's degree candidates (Master's thesis). Dalian Medical University. [https://doi.org/10.26994/d.cnki.gdlyu.2020.000271](https://doi.org/10.26994/d.cnki.gdlyu.2020.000271)

Yang, M. Q. (2022). Research on the mediating role of cognitive style between aesthetic ability and innovative ability (Master's thesis). Qufu Normal University. [https://doi.org/10.27267/d.cnki.gqfsu.2022.000665](https://doi.org/10.27267/d.cnki.gqfsu.2022.000665)

Yang, N. (2016). The brain mechanism of graphical creativity and its relationship with personality traits: An fNIRS study (Master's thesis). Tianjin Normal University. [https://kns.cnki.net/kcms2/article/abstract?v=YtMUyApXHwZvCv-UMOhFRCcxO9nLdUVKS_oUTJHNWMlm4zzsVfm_sx3Z4mpjlS_KIguNFTTSiqzuvwFpWqgFQZjIMIYUHxW3kV3waXClxJEka3oDEKrTBdW7ueodEUZ5StOVqwJv8B78NpDO-nKwN-oqvGOfbQUnqPG_JpFgTxUgXU476pLJSA==&uniplatform=NZKPT&language=CHS](https://kns.cnki.net/kcms2/article/abstract?v=YtMUyApXHwZvCv-UMOhFRCcxO9nLdUVKS_oUTJHNWMlm4zzsVfm_sx3Z4mpjlS_KIguNFTTSiqzuvwFpWqgFQZjIMIYUHxW3kV3waXClxJEka3oDEKrTBdW7ueodEUZ5StOVqwJv8B78NpDO-nKwN-oqvGOfbQUnqPG_JpFgTxUgXU476pLJSA==&uniplatform=NZKPT&language=CHS)

Yang, X. J., Li, D. Y., & Sun, X. M. (2011). Creative personality of upper-grade primary school children and its relationship with family environment. Modern Education Science, (12), 107-109. [https://doi.org/10.13980/j.cnki.xdjykx.gjyj.2011.12.046](https://doi.org/10.13980/j.cnki.xdjykx.gjyj.2011.12.046)

Yang, X., Lei, K. C., & Chen, J. J. (2013). The latest survey on the development of creativity among Shanghai adolescents. Contemporary Youth Studies, (05), 5-12.

Ye, Q. (2015). The relationship between mind wandering and creative problem solving (Master's thesis). Zhejiang Normal University. [https://kns.cnki.net/kcms2/article/abstract?v=YtMUyApXHwZcUwj5BgxEDEjbYc0mJUKWjrBAtwXBTHFGn1cODYUyO2IHDbEn02RumIcFt9Fz9j-hIICdgIlFu2TUvXJPjA6QIDY_M9CZNl8NXkn8sJjR2z8fIv9Stn92RJ2H6gIOrrVEh6o89B2OObgNRHMmdOsZgfQRcA71KyaHF9pQ59vCbw==&uniplatform=NZKPT&language=CHS](https://kns.cnki.net/kcms2/article/abstract?v=YtMUyApXHwZcUwj5BgxEDEjbYc0mJUKWjrBAtwXBTHFGn1cODYUyO2IHDbEn02RumIcFt9Fz9j-hIICdgIlFu2TUvXJPjA6QIDY_M9CZNl8NXkn8sJjR2z8fIv9Stn92RJ2H6gIOrrVEh6o89B2OObgNRHMmdOsZgfQRcA71KyaHF9pQ59vCbw==&uniplatform=NZKPT&language=CHS)

Yi, S. H., Liang, M., & Huang, D. X. (2014). A study on the creativity tendency of forensic medicine undergraduates. Basic Medical Education, 16(6), 484-486. [https://doi.org/10.13754/j.issn2095-1450.2014.06.28](https://doi.org/10.13754/j.issn2095-1450.2014.06.28)

Yin, Y. L. (2020). A study on the mediating role of flow in the relationship between personality traits and creativity in high school students (Master's thesis). Qufu Normal University. [https://doi.org/10.27267/d.cnki.gqfsu.2020.001046](https://doi.org/10.27267/d.cnki.gqfsu.2020.001046)

You, H. Y. (2007). A study on the relationship among mental health, self-consciousness, and creativity in junior high school students (Master's thesis). Fujian Normal University. [https://kns.cnki.net/kcms2/article/abstract?v=lSOmZDqoX8PpzDoeGxr18iZs8had0bdTA5fQp-wMKRgn419kemP8NYkGAKJIc9CptAcDYeS7B9FeVp2Vphpl3Q_I4pjJ221vmbOBHme6waH0kpdXzRWf2__ja5KV_GZcTAE4YN3BgH8TN8tYV-wLbymq5EmUPvFdAP4OsKgnIF2vxlZ_ingg8g==&uniplatform=NZKPT&language=CHS](https://kns.cnki.net/kcms2/article/abstract?v=lSOmZDqoX8PpzDoeGxr18iZs8had0bdTA5fQp-wMKRgn419kemP8NYkGAKJIc9CptAcDYeS7B9FeVp2Vphpl3Q_I4pjJ221vmbOBHme6waH0kpdXzRWf2__ja5KV_GZcTAE4YN3BgH8TN8tYV-wLbymq5EmUPvFdAP4OsKgnIF2vxlZ_ingg8g==&uniplatform=NZKPT&language=CHS)

Yu, H. (2016). A study on the relationship among adolescent peer attachment, self-worth, and creative tendency (Master's thesis). Southeast University. [https://kns.cnki.net/kcms2/article/abstract?v=YtMUyApXHwbWoTTeuSU-iwClTg6s8ZnF2bqFyRSfJD37wKtou1ZVNW1GFTI218Z75mHpuR2ixDE8rUNpCOnRTC7ZIUewJ5WxfTJ-a8qzgWF-Pm6GopoiEeEaA13_tMcCcdJkklqwjyIzyu0PC-fn2YAyYpMUkMHhX4S-QAPvZLeqEAaVIdtUlA==&uniplatform=NZKPT&language=CHS](https://kns.cnki.net/kcms2/article/abstract?v=YtMUyApXHwbWoTTeuSU-iwClTg6s8ZnF2bqFyRSfJD37wKtou1ZVNW1GFTI218Z75mHpuR2ixDE8rUNpCOnRTC7ZIUewJ5WxfTJ-a8qzgWF-Pm6GopoiEeEaA13_tMcCcdJkklqwjyIzyu0PC-fn2YAyYpMUkMHhX4S-QAPvZLeqEAaVIdtUlA==&uniplatform=NZKPT&language=CHS)

Yu, J. Y. (2009a). The application of neural networks in the research on middle school students’ scientific creativity. *In Proceedings of the 3rd China Intelligent Computing Conference (pp. 39–41)*. Department of Psychology, Nanjing Normal University.

Yu, J. Y. (2009b). The application of neural networks in the study of scientific creativity of middle school students. In *Proceedings of the 3rd China Conference on Intelligent Computing* (pp. 39–41). Department of Psychology, Nanjing Normal University.

Yu, J. Y., & Qian, J. X. (2010). Analysis of creative personality characteristics of junior high school students using SOM. In Proceedings of the 4th China Conference on Intelligent Computing (pp. 200–202). Department of Psychology, Nanjing Normal University; College of International Education, Nanjing Normal University.

Zeng, Z. Q. (2015). *The relationship between executive function and creativity in 9-year-old children* (Master's thesis). Beijing Forestry University. [https://kns.cnki.net/kcms2/article/abstract?v=YtMUyApXHwav4xcXToG9c7TxuqrS1IAaYFLKisEpJ9a00vMcehGgBjg_uJuTCt8MdV7fHSyy9PLX6A7411stjKwmVPOTMMRihSBJzdAwJYz5p6XJWs5hsfIMYJF_t_g5IgavssdTHdjYsuZHhX2DuGmIVdcQnTlFKoPwwZTPKGukwVJ1He5Fmw==&uniplatform=NZKPT&language=CHS](https://kns.cnki.net/kcms2/article/abstract?v=YtMUyApXHwav4xcXToG9c7TxuqrS1IAaYFLKisEpJ9a00vMcehGgBjg_uJuTCt8MdV7fHSyy9PLX6A7411stjKwmVPOTMMRihSBJzdAwJYz5p6XJWs5hsfIMYJF_t_g5IgavssdTHdjYsuZHhX2DuGmIVdcQnTlFKoPwwZTPKGukwVJ1He5Fmw==&uniplatform=NZKPT&language=CHS)

Zhai, J. M. (2019). Instructional design and implementation based on Scratch to promote the development of innovative ability in junior high school students (Master's thesis). Ningbo University. [https://doi.org/10.27256/d.cnki.gnbou.2019.000337](https://doi.org/10.27256/d.cnki.gnbou.2019.000337)

Zhang, C. Z., Wang, H., Hu, J. Y., Zhou, Y. H., Jiang, M., Yan, Y. E., & Zhang, L. (2008). The role of exploratory experiments in cultivating college students' creativity. China Higher Medical Education, (06), 11-12.

Zhang, H. J., Wang, L., & Zhang, M. (2018). The relationship among creative cognitive style, creative personality, and creative thinking. Studies of Psychology and Behavior, 16(1), 51-57

Zhang, H. M., Liu, C. Y., Li, H., Dai, Y. M., Jia, X. F., & Tao, R. (2014). Creative tendency of junior high school students with internet addiction and its correlation with parenting styles. Chinese General Practice, 17(10), 1176–1179.

Zhang, J. P. (2004). A study on the relationship among general self-efficacy, need for cognition, and creativity in middle school students (Master’s thesis, Shanxi University). Retrieved from https://kns.cnki.net/kcms2/article/abstract?v=VYuoLtjwl8O-ROp1-2PSyPCxgYT6QKFVt8rESY_8EZKXnVbQx1Sxn2UM9oWC8Bd4rC-3elmgdE7PRHz53ZbQQSomTYWzefQyw196jZuCy10-Jv13Jdx0TBXn65sz2x4SJB3JuTQDjmPW4i9Hd8RUG18I6DPlzxmMALsnKSGyEzTZnT0-All9qg==

Zhang, L. J., Cao, J. J., & Zeng, T. D. (2012). Investigation on creative thinking of college students. China Journal of Health Psychology, 20(9), 1419–1421. [https://doi.org/10.13342/j.cnki.cjhp.2012.09.032](https://doi.org/10.13342/j.cnki.cjhp.2012.09.032)

Zhang, M. M. (2017). *A study on the correlation between self-consciousness and creativity of left-behind children in grades 4-6: Taking three primary schools in Hubei Province as examples* (Master's thesis). Hubei Normal University.

Zhang, M. R. (2020). Research on the impact of participation in innovation and entrepreneurship education on college students' creativity (Master's thesis). Changsha University of Science & Technology. [https://doi.org/10.26985/d.cnki.gcsjc.2020.000439](https://doi.org/10.26985/d.cnki.gcsjc.2020.000439)

Zhang, W. J., Luo, Y. F., & Zhang, M. (2022). A study on the relationship among achievement motivation, sense of security, and creativity tendency of college students. Journal of Huaiyin Teachers College (Natural Science Edition), 21(1), 42–47. [https://doi.org/10.16119/j.cnki.issn1671-6876.2022.01.015](https://doi.org/10.16119/j.cnki.issn1671-6876.2022.01.015)

Zhang, X. X. (2021). A study on the influence of rope skipping on the creativity of primary school students (Master's thesis). Southwest University. [https://doi.org/10.27684/d.cnki.gxndx.2021.004488](https://doi.org/10.27684/d.cnki.gxndx.2021.004488)

Zhang, Y., & Xu, H. W. (2011). An empirical study on creativity tendency of master's students and its educational interpretation. China Higher Education Research, (05), 41-44. [https://doi.org/10.16298/j.cnki.1004-3667.2011.05.015](https://doi.org/10.16298/j.cnki.1004-3667.2011.05.015)

Zhang, Y., Wang, C. Y., He, Y. J., & Liu, J. J. (2023). College students' creativity and entrepreneurial intention: The moderating effect of self-efficacy and entrepreneurship education. Science and Education Wenhui, (16), 1–6. [https://doi.org/10.16871/j.cnki.kjwh.2023.16.001](https://doi.org/10.16871/j.cnki.kjwh.2023.16.001)

Zhang,Y., Zhang L., Liang, J., Duan, J., Ji, L., Meng, W., Wang, S., Huang, Q., Lan, X., Kong, P. (1999). Experimental study on cultivating creativity in natural teaching. *Journal of Luoyang Teachers College*, (2), 129-135, 139. https://doi.org/10.16594/j.cnki.41-1302/g4.1999.02.048

Zhao, L. Y. (2020). A case study on the construction of a high school mathematics learning environment aimed at fostering creativity (Doctoral dissertation). East China Normal University. [https://doi.org/10.27149/d.cnki.ghdsu.2020.000028](https://doi.org/10.27149/d.cnki.ghdsu.2020.000028)

Zhao, X. M. (2016). Research on the cultivation of creativity in primary school students (Master's thesis). Shaanxi Normal University. [https://kns.cnki.net/kcms2/article/abstract?v=lSOmZDqoX8MYuLiFywNvMO2AYzQ-9CFcqYRQKHCTk8PNejmi1FNGDt4X40iJOEkpVwiVoWxnj8JK3ToZiJw-XlZ19D--fGUdgZvaq7-5WVhO2xnJ4-syFYnGxxTYCdxVugZM73blt085wP5ysSq6ETW0Z8UQKSVZiuPn3Q200YOyQUBYFxMeDQ==&uniplatform=NZKPT&language=CHS](https://kns.cnki.net/kcms2/article/abstract?v=lSOmZDqoX8MYuLiFywNvMO2AYzQ-9CFcqYRQKHCTk8PNejmi1FNGDt4X40iJOEkpVwiVoWxnj8JK3ToZiJw-XlZ19D--fGUdgZvaq7-5WVhO2xnJ4-syFYnGxxTYCdxVugZM73blt085wP5ysSq6ETW0Z8UQKSVZiuPn3Q200YOyQUBYFxMeDQ==&uniplatform=NZKPT&language=CHS)

Zhao, Z. Q. (2016). The learning effects of the "Analects" recitation class for children and its impact on creativity (Master's thesis). Shaanxi Normal University. [https://kns.cnki.net/kcms2/article/abstract?v=YtMUyApXHwanh8UIDd3dsAtQHPrldzuK_5JJIXrFHGiGolLbOcJCP7-scmu4f7c44L0TocWajoZPxcsBOv0oK-iyqmuuX2BZ7sZU2vzh2mzn7kkKwxCGJ01BkZ4mh1ewzAkS2JnmO0m2MfbEsFfyw2y24xywSBgVxlHxAAz0ul2ULqAS6BB6nw==&uniplatform=NZKPT&language=CHS](https://kns.cnki.net/kcms2/article/abstract?v=YtMUyApXHwanh8UIDd3dsAtQHPrldzuK_5JJIXrFHGiGolLbOcJCP7-scmu4f7c44L0TocWajoZPxcsBOv0oK-iyqmuuX2BZ7sZU2vzh2mzn7kkKwxCGJ01BkZ4mh1ewzAkS2JnmO0m2MfbEsFfyw2y24xywSBgVxlHxAAz0ul2ULqAS6BB6nw==&uniplatform=NZKPT&language=CHS)

Zheng, X. (2014). Research on anchored instruction model and the development of students' innovative ability (Master's thesis). Nanjing Normal University. [https://kns.cnki.net/kcms2/article/abstract?v=YtMUyApXHwaYG3btINPRUSuKSFPdeNM1MWHkoGtEeAWm9uMI1E8bRXadHBuy6kypcwNDo3kJjaiSaQ2UD6n8bGtG3MKm23YUNQyh5SKGKQbEn-xrV_cmtfkAbA2T2dXbGtII9kXNlx7kZHkSI-BGT7ZjrDIQKNqkbniEfBPyZm1C8-gtzYcOyg==&uniplatform=NZKPT&language=CHS](https://kns.cnki.net/kcms2/article/abstract?v=YtMUyApXHwaYG3btINPRUSuKSFPdeNM1MWHkoGtEeAWm9uMI1E8bRXadHBuy6kypcwNDo3kJjaiSaQ2UD6n8bGtG3MKm23YUNQyh5SKGKQbEn-xrV_cmtfkAbA2T2dXbGtII9kXNlx7kZHkSI-BGT7ZjrDIQKNqkbniEfBPyZm1C8-gtzYcOyg==&uniplatform=NZKPT&language=CHS)

Zheng, X. K., & Zhang, L. H. (2022). The relationship between thinking styles and creative tendency of college volleyball majors: The mediating role of learning strategies. In Abstract compilation of the 12th National Sports Science Conference: Poster presentation (Sports Psychology Branch) (pp. 244–245). Shenyang Sport University; College of Sport Training, Shenyang Sport University. [https://doi.org/10.26914/c.cnkihy.2022.009445](https://doi.org/10.26914/c.cnkihy.2022.009445)

Zheng, X. Y., Wang, Q. Y., Zhang, X., & Wang, T. Y. (2013). Research on the creative development of college students: Based on a survey of students at Beijing University of Chemical Technology. Tsinghua Journal of Education, 34(4), 121-124. [https://doi.org/10.14138/j.1001-4519.2013.04.015](https://doi.org/10.14138/j.1001-4519.2013.04.015)

Zhong, L. (2020). An experimental exploration of integrating STEAM concepts into science classrooms for cultivating students' creativity (Master's thesis). Hangzhou Normal University. [https://doi.org/10.27076/d.cnki.ghzsc.2020.000332](https://doi.org/10.27076/d.cnki.ghzsc.2020.000332)

Zhou, L. F. (2021). A study on the impact of divergent sports teaching on college students' creativity. Sports Science Research, 25(2), 81-87. [https://doi.org/10.19715/j.tiyukexueyanjiu.2021.02.015](https://doi.org/10.19715/j.tiyukexueyanjiu.2021.02.015)

Zhu, B. T. (2021). Research on the practice of primary school information technology unit teaching under the concept of STEM (Master's thesis). Inner Mongolia Normal University. [https://doi.org/10.27230/d.cnki.gnmsu.2021.000529](https://doi.org/10.27230/d.cnki.gnmsu.2021.000529)

Zhu, H. X., & Song, D. R. (2016). A study on the relationship between cognitive style and creative tendency of college students. Journal of Chifeng University (Natural Science Edition), 32(24), 69-70. [https://doi.org/10.13398/j.cnki.issn1673-260x.2016.24.026](https://doi.org/10.13398/j.cnki.issn1673-260x.2016.24.026)

Zhu, L. Y., & Wang, X. Z. (2017). The influence of classroom teaching and extracurricular learning on college students' creativity tendency: An empirical study based on nine universities in Guangdong. Education and Teaching Research, 31(3), 5-10. [https://doi.org/10.13627/j.cnki.cdjy.2017.03.002](https://doi.org/10.13627/j.cnki.cdjy.2017.03.002)

Zhu, X. H. (2001). A study on the relationship between children’s learning motivation types and creative tendency. *Journal of Nanjing Normal University (Social Science Edition)*, (6), 90–95.

Zhu, Y., & Yang, L. (2017). The influence of knowledge structure on college students’ creative tendency: An empirical study based on nine universities in Guangdong. *Journal of Guangdong University of Foreign Studies*, 28(1), 123–128.

Zou, L. Q., Zhao, Y., Zhao, J. X., Zhu, M. M., & Liu, Y. (2019). The influence of situational teaching method combined with PDCA cycle on nursing students’ creativity: An empirical study based on experimental teaching of fundamental nursing. *Research on Communication Power*, 3(9), 206.
