# Supplementary material for: Creative tendency of Chinese students: a meta-analysis, 1999–2025
Source: Front Psychol. 2026 Oct 20;17:1957163. doi: 10.3389/fpsyg.2026.1957163 (PMC13727631; doi:10.3389/fpsyg.2026.1957163)
Supplement: Supplementary file 2 [file Table_1.DOCX]

**Table S1 study features extracted from primary studies**

| **Study ID** | **Publication year** | **Valid N** | **Female  %** | **Reliability** | **Validity** | **Hometown location** | **Grades** | **Scoring  method** |
| --- | --- | --- | --- | --- | --- | --- | --- | --- |
| An, 2011 | 2011 | 108 | NA | NA | NA | NA | CS | 3 |
| An, 2023 | 2023 | 52 | NA | 0.804 | 0.825 | NA | CS | 5 |
| Cai, 2017 | 2017 | 200 | 43.00% | NA | NA | NA | PSS | 3 |
| Cai, 2018 | 2018 | 236 | NA | NA | NA | NA | CS | 5 |
| Cai, Xiao, Bai, 2001 | 2001 | 63 | 55.56% | NA | NA | NA | PSS | NA |
| Cai, Zhu, 2007 | 2007 | 524 | NA | NA | NA | NA | HSS | 3 |
| Cao, 2021 | 2021 | 967 | 43.23% | NA | NA | NA | CS | 3 |
| Chai, Han, 2008 | 2008 | 200 | 93.50% | NA | NA | NA | MSS | 5 |
| Chen, 2013 | 2013 | 178 | 60.11% | 0.401-0.708 | NA | NA | PSS | 3 |
| Chen, 2014 | 2014 | 48 | NA | NA | NA | NA | CS | NA |
| Chen, 2016 | 2016 | 139 | 46.76% | NA | NA | NA | MSS | 3 |
| Chen, 2020 | 2019 | 293 | 53.24% | 0.919-0.992 | NA | NA | MSS, HSS | 3 |
| Chen, 2022 | 2022 | 47 | NA | 0.49-0.81 | NA | NA | PSS | 3 |
| Chen, Liang, Wang, 2020 | 2020 | 420 | 45.48% | NA | NA | NA | CS | 3 |
| Chen, N. 2020 | 2020 | 530 | 47.55% | NA | NA | NA | CS | 5 |
| Chen, Wang, 2021 | 2021 | 2238 | 76.68% | 0.86 | NA | Town, Rural | HSS, HSS | 3 |
| Chen, Wei, Yu, Wang, 2011 | 2011 | 250 | 47.60% | NA | NA | NA | PSS | 3 |
| Chen, Y. 2020 | 2020 | 995 | 49.55% | NA | NA | NA | PSS | 3 |
| Chen, Z. 2020 | 2020 | 247 | 60.73% | NA | NA | Town, Rural | CS | 3 |
| Chen, Zhang, 2021 | 2021 | 196 | 56.12% | 0.82 | NA | NA | CS, CS | 3 |
| Chen, Zhu, 2010 | 2010 | 469 | 62.47% | NA | NA | NA | CS | 3 |
| Cheng, 2023 | 2023 | 811 | 52.03% | NA | NA | Town, Rural | MSS, HSS | 3 |
| Chu, 2024 | 2024 | 30 | NA | NA | NA | NA | MSS | 3 |
| Dai, Chen, Mao, Xu, 2024 | 2024 | 427 | NA | NA | NA | NA | PSS | 3 |
| Dai, Xu, 2010 | 2010 | 200 | 44.00% | NA | NA | Town, Rural | CS | 3 |
| Deng, 2013 | 2013 | 34 | NA | NA | NA | NA | MSS | 3 |
| Du, 2022 | 2022 | 40 | NA | NA | NA | NA | HSS | 3 |
| Du, Niu, 2013 | 2013 | 230 | 56.52% | NA | NA | NA | CS | 3 |
| Du, 2009 | 2005 | 449 | 52.34% | NA | NA | NA | CS | 3 |
| Fang, 2013 | 2013 | 490 | 92.86% | NA | NA | Town, Rural | CS | 3 |

**Table S1 study features extracted from primary studies (continued)**

| **Study ID** | **Publication year** | **Valid N** | **Female  %** | **Reliability** | **Validity** | **Hometown location** | **Grades** | **Scoring  method** |
| --- | --- | --- | --- | --- | --- | --- | --- | --- |
| Feng, 2021 | 2021 | 25 | 56.00% | NA | NA | NA | MSS | 3 |
| Fu, 2013 | 2013 | 60 | NA | NA | NA | NA | CS, CS | 3 |
| Fu, 2023 | 2023 | 48 | NA | NA | NA | NA | CS | 3 |
| Gai, 2021 | 2021 | 236 | 52.97% | NA | NA | NA | CS | 3 |
| Gao, 2023 | 2023 | 700 | 51.29% | NA | NA | Town, Rural | PSS, MSS, MSS | 3 |
| Gao, Zeng, 2012 | 2012 | 1139 | 46.80% | NA | NA | NA | CS | 3 |
| Gou, 2021 | 2021 | 32 | NA | NA | NA | NA | PSS | 5 |
| Guo, E. 2017 | 2017 | 43574 | 32.56% | NA | NA | Town, Rural | GS, GS, GS | 5 |
| Guo, Wu, Ding, 2018 | 2018 | 1146 | 62.74% | NA | NA | NA | NA | 3 |
| Guo, 2018 | 2017 | 294 | 97.96% | 0.80-0.84 | NA | NA | PSS | 3 |
| Han, 2012 | 2012 | 374 | 50.00% | 0.809-0.849 | NA | NA | HSS | 3 |
| He, 2000 | 2000 | 48 | 43.75% | NA | NA | NA | PSS | NA |
| He, 2019 | 2019 | 2428 | 51.36% | 0.8772 | NA | Town, Rural | CS | NA |
| Hu, Zhao, Jia, Chen, 2017 | 2017 | 30 | NA | 0.49-0.891 | NA | NA | HSS | 3 |
| Huang, 2010 | 2010 | 44 | 52.27% | NA | NA | NA | CS | 3 |
| Huang, 2018 | 2018 | 320 | 95.00% | 0.79 | NA | Town, Rural | HSS | 3 |
| Huang, 2022 | 2022 | 42 | 47.62% | NA | NA | NA | CS | 3 |
| Huang, 2023 | 2023 | 83 | 54.22% | NA | NA | NA | CS, CS | 3 |
| Huang, 2024 | 2024 | 52 | NA | NA | NA | NA | MSS | 3 |
| Ji, 2019 | 2019 | 969 | NA | 0.88 | NA | NA | PSS, MSS | 5 |
| Ji, Yu, Shen, 2019 | 2019 | 414 | 56.76% | 0.401-0.877 | NA | NA | PSS | NA |
| Jia, 2009 | 2009 | 92 | NA | 0.809-0.849 | NA | NA | MSS, MSS, MSS | 5 |
| Jia, 2017 | 2017 | 50 | 54.00% | NA | NA | NA | PSS | 3 |
| Jiang, 2016 | 2016 | 40 | 50.00% | 0.83 | NA | NA | PSS | 3 |
| Jiang, 2021 | 2021 | 20 | NA | NA | NA | NA | MSS | 3 |
| Jiao, 2023 | 2023 | 192 | NA | NA | NA | NA | CS, CS | 3 |
| Kai, 1999 | 1999 | 105 | 52.38% | NA | NA | NA | PSS | NA |
| Kong, 2016 | 2016 | 178 | NA | NA | NA | Town, Rural | HSS | 3 |
| Lai, 2019 | 2019 | 30 | NA | NA | NA | NA | MSS, MSS | 3 |
| Lan, Li, Wei, Du, Li, 2019 | 2019 | 105 | 41.90% | 0.82 | NA | NA | MSS, HSS | 3 |
| Li, 2007 | 2007 | 818 | 49.51% | NA | NA | NA | MSS | NA |
| Li, 2015 | 2015 | 2985 | 49.88% | NA | NA | NA | HSS | 3 |

**Table S1 study features extracted from primary studies (continued)**

| **Study ID** | **Publication year** | **Valid N** | **Female  %** | **Reliability** | **Validity** | **Hometown location** | **Grades** | **Scoring  method** |
| --- | --- | --- | --- | --- | --- | --- | --- | --- |
| Li, Cao, Dong, Kong, Zhu, 2012 | 2012 | 465 | 79.78% | NA | NA | NA | CS, CS | 3 |
| Li, F.2009 | 2009 | 60 | 10.00% | NA | NA | NA | NA | 3 |
| Li, He, 2023 | 2023 | 300 | 82.67% | NA | NA | NA | NA | 3 |
| Li, Hu, 2016 | 2016 | 8257 | NA | NA | NA | Town, Rural | CS | 3 |
| Li, W.2009 | 2009 | 431 | 57.77% | NA | NA | NA | NA | NA |
| Li, Zhang, He, 2005 | 2005 | 595 | 52.27% | NA | NA | NA | NA | 3 |
| Li,D. 2021 | 2021 | 35 | NA | NA | NA | NA | MSS | 5 |
| Li,Y. 2021 | 2021 | 186 | NA | NA | NA | NA | CS | 3 |
| Liang, 2014 | 2014 | 123 | 56.91% | 0.879 | NA | NA | MSS | 3 |
| Liang, 2019 | 2019 | 20 | NA | NA | NA | NA | PSS, MSS | 3 |
| Liang, 2024 | 2024 | 66 | 45.45% | 0.525-0.884 | NA | NA | HSS | 3 |
| Liang, C. 2021 | 2021 | 5248 | NA | NA | NA | NA | MSS, HSS | NA |
| Liang, Q.2021 | 2021 | 64 | NA | NA | NA | NA | PSS | 3 |
| Lin, 2006 | 2006 | 48 | NA | NA | NA | NA | MSS | 3 |
| Liu, 2007 | 2007 | 50 | NA | NA | NA | NA | CS | 3 |
| Liu, 2008 | 2008 | 500 | NA | NA | NA | NA | PSS | 5 |
| Liu, 2010 | 2010 | 216 | 74.54% | NA | NA | NA | CS | 3 |
| Liu, 2011 | 2011 | 276 | 54.71% | NA | NA | NA | HSS, HSS | 3 |
| Liu, 2013 | 2013 | 193 | 56.99% | 0.714-0.758 | NA | NA | MSS | 3 |
| Liu, 2014 | 2014 | 435 | 53.56% | NA | NA | NA | CS, CS | 3 |
| Liu, 2016 | 2016 | 150 | 50.67% | 0.549-0.670 | NA | NA | CS | 3 |
| Liu, F.2020 | 2020 | 1216 | 51.56% | 0.819 | NA | NA | CS | 3 |
| Liu, H.2023 | 2023 | 569 | 49.38% | NA | NA | Town, Rural | PSS, HSS | 3 |
| Liu, Liu, Qiu, Jin, Zhou, 2016 | 2016 | 120 | 62.50% | NA | NA | NA | PSS, MSS, HSS | 3 |
| Liu, M. 2020 | 2020 | 43 | NA | 0.85 | NA | NA | HSS, HSS,HSS | 3 |
| Liu, S. 2020 | 2020 | 160 | 50.00% | 0.864 | NA | NA | PSS | 3 |
| Liu, Shan, 2013 | 2013 | 92 | NA | NA | NA | NA | GS | 5 |
| Liu, W.2023 | 2023 | 41 | 43.90% | NA | NA | NA | PSS | 3 |
| Liu, Y. 2020 | 2020 | 20 | NA | NA | NA | NA | CS | 3 |
| Liu, Y.2023 | 2023 | 87 | NA | NA | NA | NA | MSS | 3 |
| Liu, Yang, Li, 2011 | 2011 | 187 | 50.27% | 0.81-0.85 | NA | NA | MSS | 3 |
| Liu, Z. 2020 | 2020 | 276 | NA | NA | NA | NA | PSS, MSS | NA |

**Table S1 study features extracted from primary studies (continued)**

| **Study ID** | **Publication year** | **Valid N** | **Female  %** | **Reliability** | **Validity** | **Hometown location** | **Grades** | **Scoring  method** |
| --- | --- | --- | --- | --- | --- | --- | --- | --- |
| Liu, Zhang, 2017 | 2017 | 1650 | 52.36% | 0.882 | NA | Town, Rural | MSS, HSS | NA |
| Liu, Zhang, Shi, Tao, 2013 | 2013 | 306 | 54.58% | NA | NA | NA | CS | 3 |
| Liu, Zhang, Zhang, Li, Xue, Gao, Tao, Li, 2014 | 2014 | 61 | NA | NA | NA | NA | NA | 3 |
| Lou, Chou, Shih, Chung, 2017 | 2017 | 60 | NA | NA | NA | NA | PSS | 3 |
| Lu, Lo, Syu, 2021 | 2021 | 21 | 57.14% | NA | NA | NA | CS | 3 |
| Luo, 2010 | 2010 | 1008 | NA | NA | NA | NA | CS | 3 |
| Luo, 2020 | 2020 | 62 | NA | 0.814 | NA | NA | NA | 3 |
| Lv, 2024 | 2024 | 54 | NA | NA | NA | NA | HSS | 3 |
| Lv, J. 2016 | 2016 | 823 | 48.36% | NA | NA | NA | CS, CS | 3 |
| Lv, Y. 2016 | 2016 | 759 | 56.65% | NA | NA | NA | HSS | 5 |
| Ma, 2024 | 2024 | 948 | 62.03% | NA | NA | Town, Rural | HSS | 3 |
| Meng, L. 2021 | 2021 | 12 | NA | NA | NA | NA | PSS | 3 |
| Meng, Z. 2021 | 2021 | 48 | 50.00% | NA | NA | NA | MSS | 3 |
| Mu, 2016 | 2016 | 35 | NA | NA | NA | NA | CS | 3 |
| Ni, 2020 | 2020 | 39 | 120.51% | NA | NA | NA | HSS | 5 |
| Nie, Zheng, 2005 | 2005 | 3727 | 48.97% | NA | NA | NA | MSS | 3 |
| Niu, 2018 | 2018 | 325 | 32.31% | 0.59 | NA | Town, Rural | HSS | 5 |
| Niu, 2021 | 2021 | 30 | NA | 0.834 | NA | NA | PSS | 3 |
| Niu, 2022 | 2022 | 420 | 55.00% | NA | NA | NA | MSS | 3 |
| Ou, 2016 | 2016 | 648 | 48.61% | NA | NA | NA | MSS | 5 |
| Pan, Xu, Wu, Yan, Gu, 2016 | 2016 | 400 | NA | NA | NA | NA | NA | 3 |
| Qian, 2007 | 2007 | 187 | 34.22% | NA | NA | NA | MSS, HSS | 3 |
| Qian, 2009 | 2009 | 44 | NA | NA | NA | NA | MSS | 3 |
| Qian, 2013 | 2013 | 686 | 50.44% | 0.873 | NA | NA | MSS, HSS | 3 |
| Qin, 2018 | 2018 | 794 | NA | NA | NA | NA | CS | 3 |
| Qiu, 2020 | 2020 | 24 | 20.83% | 0.53-0.88 | NA | NA | PSS | 3 |
| Ren, Liu, Wang, Chen, 2020 | 2020 | 70 | 61.43% | 0.79 | NA | NA | PSS | 3 |
| Shen, 2024 | 2024 | 142 | NA | 0.96 | NA | NA | PSS | 3 |
| Sheng, 2018 | 2018 | 197 | 47.21% | 0.82-0.86 | 0.81-0.86 | NA | PSS | 3 |
| Shi, 2006 | 2006 | 334 | 49.70% | NA | NA | NA | CS, CS | 5 |

**Table S1 study features extracted from primary studies (continued)**

| **Study ID** | **Publication year** | **Valid N** | **Female  %** | **Reliability** | **Validity** | **Hometown location** | **Grades** | **Scoring  method** |
| --- | --- | --- | --- | --- | --- | --- | --- | --- |
| Shi, Xu, 2009 | 2009 | 346 | 69.65% | 0.834 | 0.79 | NA | CS | 3 |
| Song, Zhu, Zhang, 2005 | 2005 | 87 | NA | 0.79 | NA | NA | CS | 3 |
| Su, 2010 | 2010 | 223 | 48.43% | 0．49～0．81 | NA | NA | NA | 3 |
| Su, 2024 | 2024 | 44 | NA | NA | NA | NA | MSS | 3 |
| Sun, 2020 | 2020 | 324 | 45.37% | 0.53-0.88 | NA | Town, Rural | MSS | 3 |
| Sun, 2021 | 2021 | 257 | 49.81% | NA | NA | NA | CS | 3 |
| Tan, 2024 | 2024 | 390 | 49.74% | NA | NA | NA | MSS | 3 |
| Tang, 2024 | 2024 | 398 | 49.25% | NA | NA | NA | PSS | 3 |
| Tian, 2022 | 2022 | 946 | NA | 0.868 | 0.882 | NA | MSS, HSS | 3 |
| Teng, 2014 | 2014 | 785 | 64.71% | 0.867 | NA | Town, Rural | MSS | 5 |
| Tong, 2019 | 2019 | 273 | 38.10% | NA | NA | NA | HSS | 7 |
| Tong, Zhong, Yang, Liu, Yang, Zhao, Hou, 2025 | 2025 | 950 | NA | NA | NA | NA | NA | 3 |
| Wang, 2001 | 2001 | 160 | 50.00% | NA | NA | NA | HSS, HSS, HSS | 3 |
| Wang, 2010 | 2010 | 52 | NA | NA | NA | NA | NA | NA |
| Wang, 2012 | 2012 | 45 | NA | NA | NA | NA | MSS | 3 |
| Wang, 2021 | 2021 | 1109 | NA | NA | NA | NA | CS,GS | 3 |
| Wang, 2023 | 2023 | 40 | NA | NA | NA | NA | HSS | 3 |
| Wang, 2024 | 2024 | 44 | NA | 0.751 | 0.753 | NA | NA | 3 |
| Wang, Kuang, Wang, Ju, 2005 | 2005 | 407 | NA | NA | NA | NA | CS | 3 |
| Wang, Li, Zhang, 2006 | 2006 | 222 | 49.55% | NA | NA | NA | HSS, HSS | 3 |
| Wang, Liu, Hou, Yang, 2012 | 2012 | 240 | 44.17% | NA | NA | NA | PSS | 3 |
| Wang, P. 2020 | 2020 | 500 | 46.80% | NA | NA | NA | CS | 5 |
| Wang, Pang, 2023 | 2023 | 82 | 56.10% | 0.887 | 0.808 | NA | CS, CS | 5 |
| Wang, S. 2008 | 2008 | 324 | 43.83% | NA | NA | NA | CS | 3 |
| Wang, Shu, 2012 | 2012 | 873 | NA | 0.72 | NA | NA | HSS, HSS, HSS | 3 |
| Wang, Wang, 2014 | 2014 | 443 | 59.14% | NA | NA | NA | PSS | 3 |
| Wang, X. 2008 | 2008 | 473 | 60.47% | 0.809-0.849 | NA | NA | PSS | 5 |
| Wang, Y. 2009 | 2009 | 341 | 50.73% | NA | NA | NA | MSS, MSS | 3 |
| Wang, Y. 2020 | 2020 | 642 | 52.18% | NA | NA | NA | HSS | 5 |

**Table S1 study features extracted from primary studies (continued)**

| **Study ID** | **Publication year** | **Valid N** | **Female  %** | **Reliability** | **Validity** | **Hometown location** | **Grades** | **Scoring  method** |
| --- | --- | --- | --- | --- | --- | --- | --- | --- |
| Wang, Z. 2009 | 2009 | 950 | 49.47% | NA | NA | NA | PSS | 3 |
| Wu, 2018 | 2018 | 737 | 47.76% | NA | NA | NA | PSS | 3 |
| Wei, 2022 | 2022 | 42 | NA | 0.867 | NA | NA | CS, MSS, HSS | 3 |
| Wu, 2018 | 2018 | 737 | 47.76% | NA | NA | NA | PSS | 3 |
| Wu, 2023 | 2023 | 29 | NA | NA | NA | NA | NA | 3 |
| Wu, Qi, Guo, Shen, Zhang, 2022 | 2022 | 68 | 197.06% | NA | NA | NA | PSS | NA |
| Wu, Zhang, 2006 | 2006 | 125 | 49.60% | NA | NA | NA | PSS, MSS | NA |
| Wu, Zhang, 2009 | 2009 | 355 | 50.14% | NA | NA | NA | HSS | 3 |
| Xiao, 2017 | 2017 | 385 | 47.01% | NA | NA | NA | MSS, MSS, MSS, MSS | 3 |
| Xie, 2021 | 2021 | 33 | 54.55% | 0.765-0.810 | NA | NA | CS | 3 |
| Xiong, 2021 | 2021 | 35 | NA | 0.873 | NA | NA | CS | 5 |
| Xu, 2014 | 2014 | 261 | 29.12% | NA | NA | NA | HSS | 3 |
| Xu, 2017 | 2017 | 208 | 54.33% | NA | NA | NA | CS | 3 |
| Xu, 2023 | 2023 | 340 | 53.53% | NA | NA | NA | HSS, HSS, HSS | 3 |
| Xu, Yang, Zhu, Chen, Li, 2019 | 2019 | 364 | 53.57% | NA | NA | NA | CS | 3 |
| Xue, 2020 | 2020 | 127 | 61.42% | NA | NA | NA | CS | 3 |
| Yan, 2024 | 2024 | 32 | NA | NA | NA | NA | HSS | 3 |
| Yang, 2011 | 2011 | 239 | 40.17% | NA | NA | NA | PSS | 3 |
| Yang, 2013 | 2013 | 24 | NA | NA | NA | NA | PSS | 3 |
| Yang, 2020 | 2020 | 373 | 76.94% | NA | NA | NA | PSS | 3 |
| Yang, 2022 | 2022 | 440 | 67.73% | NA | NA | NA | PSS, HSS | 5 |
| Yang, Lei, Chen, 2013 | 2013 | 1203 | 52.70% | NA | NA | NA | MSS | 5 |
| Ye, 2015 | 2015 | 83 | 75.90% | NA | NA | NA | CS | 3 |
| Yi, Liang, Huang, 2014 | 2014 | 246 | 45.53% | NA | NA | NA | PSS | 5 |
| Yin, 2020 | 2020 | 401 | 45.14% | NA | NA | NA | CS | NA |
| You, 2007 | 2007 | 658 | 42.86% | NA | NA | NA | MSS | 3 |
| Yu, 2009a | 2009 | 476 | NA | NA | NA | NA | MSS | NA |
| Yu, 2009b | 2009 | 400 | NA | 0.809-0.849 | NA | NA | HSS | 5 |
| Yu, 2010 | 2010 | 311 | 54.02% | NA | NA | NA | MSS | NA |
| Yu, 2016 | 2016 | 522 | 54.60% | 0.40-0.87 | NA | NA | PSS, MSS, MSS | 3 |

**Table S1 study features extracted from primary studies (continued)**

| **Study ID** | **Publication year** | **Valid N** | **Female  %** | **Reliability** | **Validity** | **Hometown location** | **Grades** | **Scoring  method** |
| --- | --- | --- | --- | --- | --- | --- | --- | --- |
| Zeng, 2015 | 2015 | 286 | 63.64% | NA | NA | NA | PSS | 3 |
| Zhai, 2019 | 2019 | 47 | NA | NA | NA | NA | MSS | 3 |
| Zhang, 2004 | 2004 | 469 | 55.22% | NA | NA | NA | PSS, MSS, HSS | 5 |
| Zhang, 2011 | 2011 | 237 | NA | NA | NA | NA | CS | 3 |
| Zhang, 2017 | 2017 | 200 | 80.50% | 0.735 | NA | NA | PSS | 5 |
| Zhang, 2020 | 2020 | 16635 | NA | NA | NA | NA | CS | 5 |
| Zhang, 2021 | 2021 | 130 | NA | NA | NA | NA | CS | 3 |
| Zhang, Cao, Zeng, 2012 | 2012 | 550 | 48.73% | NA | NA | NA | HSS | 3 |
| Zhang, Liu, Li, Dai, Jia, Tao, 2014 | 2014 | 74 | NA | NA | NA | NA | GS | 3 |
| Zhang, Luo, Zhang, 2022 | 2022 | 523 | 47.42% | NA | NA | NA | CS | 3 |
| Zhang, Wang, He, Liu, 2023 | 2023 | 152 | 48.68% | 0.892 | 0.728 | NA | CS | 5 |
| Zhang, Wang, Hu, Zhou, Jiang, Yan, Zhang, 2008 | 2008 | 55 | 58.18% | NA | NA | NA | MSS, HSS | 3 |
| Zhang, Wang, Zhang, 2018 | 2018 | 315 | 47.30% | 0.74-0.89 | NA | NA | PSS | 3 |
| Zhang, Zhang, Liang et al., 1999 | 1999 | 52 | 50.00% | NA | NA | NA | PSS | NA |
| Zhao, 2016 | 2016 | 29 | NA | NA | NA | NA | PSS | 3 |
| Zhao, 2016 | 2016 | 34 | 35.29% | NA | NA | NA | PSS | 3 |
| Zhao, 2020 | 2020 | 160 | NA | NA | NA | NA | PSS | 3 |
| Zheng, 2014 | 2014 | 47 | NA | NA | NA | NA | NA | 3 |
| Zheng, 2021 | 2021 | 424 | 45.99% | 0.79 | NA | NA | NA | 3 |
| Zheng, Wang, Zhang, Wang, 2013 | 2013 | 454 | 34.80% | NA | NA | NA | MSS, HSS | 3 |
| Zhong, 2020 | 2020 | 47 | NA | 0.819 | NA | NA | PSS | 3 |
| Zhou, 2021 | 2021 | 32 | 50.00% | NA | NA | NA | CS | 3 |
| Zhu, 2001 | 2001 | 368 | 46.20% | NA | NA | NA | PSS | 3 |
| Zhu, 2021 | 2021 | 56 | NA | NA | NA | NA | MSS | 3 |
| Zhu, Song, 2016 | 2016 | 139 | 46.76% | NA | NA | NA | CS | 3 |
| Zhu, Wang, 2017 | 2019 | 1032 | 49.90% | 0.837 | NA | NA | CS | 3 |
| Zhu, Yang, 2017 | 2017 | 1027 | 49.95% | NA | NA | NA | CS | 3 |
| Zou, 2019 | 2019 | 33 | NA | NA | NA | NA | GS, GS, GS | NA |

Notes. All participants investigated are Chinese students, and and measuring tool employed is WCAP, which comprises 50 items across 4 dimensions. PSS = Primary School Students; MSS = Middle School Students; HSS = High School Students; CS = College Students; GS = Graduate School Students; NA=Missing.
